# 1,3,4-Oxadiazole Contained Sesquiterpene Derivatives: Synthesis and Microbiocidal Activity for Plant Disease

**DOI:** 10.3389/fchem.2022.854274

**Published:** 2022-02-22

**Authors:** Ali Dai, Zhiguo Zheng, Lijiao Yu, Yuanqin Huang, Jian Wu

**Affiliations:** State Key Laboratory Breeding Base of Green Pesticide and Agricultural Bioengineering, Key Laboratory of Green Pesticide and Agricultural Bioengineering, Ministry of Education, Guizhou University, Guiyang, China

**Keywords:** sesquiterpene derivatives, 1,3,4-oxadiazole, synthesis, rice bacterial blight, tobacco mosaic virus, biological activity

## Abstract

A series of 1,3,4-oxadiazole contained sesquiterpene derivatives were synthesized, and the activity of the target compounds against *Xanthomonas oryzae* pv. *oryzae* (*Xoo*)*, Xanthomonas axonopodis* pv. *citri* (*Xac*)*,* and tobacco mosaic virus (TMV) were evaluated. The biological activity results showed that the EC_50_ values of compounds **H4**, **H8**, **H11**, **H12**, **H14**, **H16**, and **H19** for *Xac* inhibitory activity were 33.3, 42.7, 56.1, 74.5, 37.8, 43.8, and 38.4 μg/ml, respectively. Compounds **H4**, **H8**, **H15**, **H19**, **H22**, and **H23** had inhibitory effects on *Xoo*, with EC_50_ values of 51.0, 43.3, 43.4, 50.5, 74.6, and 51.4 μg/ml, respectively. In particular, the curative and protective activities of compound **H8** against *Xoo in vivo* were 51.9 and 49.3%, respectively. In addition, the EC_50_ values of the inactivation activity of compounds **H4**, **H5**, **H9**, **H10**, and **H16** against TMV were 69.6, 58.9, 69.4, 43.9, and 60.5 μg/ml, respectively. The results of molecular docking indicated that compound **H10** exhibited a strong affinity for TMV-coat protein, with a binding energy of −8.88 kcal/mol. It may inhibit the self-assembly and replication of TMV particles and have an anti-TMV effect, which supports its potential usefulness as an antiviral agent.

**GRAPHICAL ABSTRACT:**
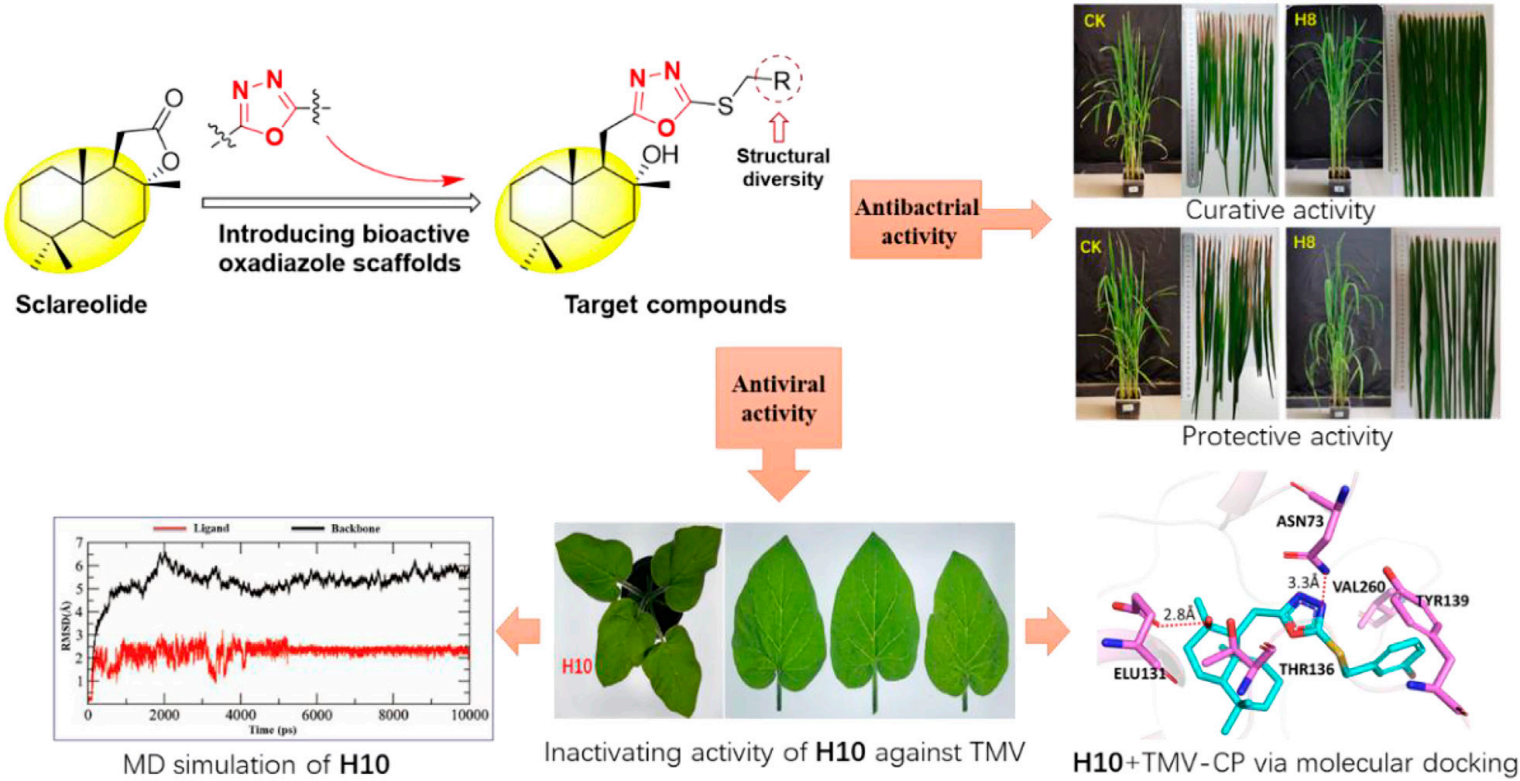


## Introduction

Most plant diseases are caused by biological agents such as bacteria, fungi, viruses, and nematodes, which have adverse impact on the growth and development of plants (Das et al., 2016). Rice bacterial blight caused by *Xanthomonas oryzae* pv. *oryzae (Xoo)* seriously threatens the growth and production of rice by affecting the tillering stage of rice (Wang et al., 2021). Citrus bacterial canker caused by *Xanthomonas axonopodis* pv. *citri (Xac)* reduces the quality and yield of fruits (Graham et al., 2004). Tobacco mosaic virus (TMV) can survive in dry plant debris for up to 100 years, and the associated plant diseases cause economic losses of more than USD 30 billion each year (Wang et al., 2018; Guo et al., 2021). At present, pesticides are the main means of controlling crop diseases and insect pests (Kemmitt et al., 2018). For plant disease, such as *Xoo, Xac* or TMV, although there are traditional medicines (such as Bismerthiazol, Thiodiazole copper, Ningnanmycin and Ribavirin), their effectiveness is limited various forms of disease and insect resistance (Buttimer et al., 2017; Liu et al., 2021). Natural products have special structural characteristics and unique biological activity mechanisms, and they are an important source for discovery of highly effective, safe, and environmentally compatible drugs (Zhang et al., 2018; Zheng and Hua, 2020; Li and Wang, 2021).

Sesquiterpenes are the most common type of terpenoids in terms of the number of compounds and the type of structural skeleton. They have thousands of representative structures and more than 300 different skeletons (Sacchettini and Poulter, 1997; Arroo, 2007). Sesquiterpenes are natural products of terpenoids found in plants, fungi, marine organisms, insects, and microorganisms. They are widely used in agriculture, medicine, perfume, cosmetics, and biofuels (Liu C.-L. et al., 2021; Liu T. et al., 2021; Mai et al., 2021). Sesquiterpenes have a variety of biological activities due to their complex three-dimensional structure, such as antiviral (Shang et al., 2016; Zhao et al., 2017), antibacterial (Duan et al., 2020; Wang et al., 2020), antifungal (Aricu et al., 2016), insecticidal and antifeedant activities (Inocente et al., 2019). In addition, at least some have excellent pharmacological activity, such as artemisinin for anti-malaria (Platon et al., 2021). There may also have anti-inflammatory (Gao et al., 2015), anti-HIV (Liu Y.-P. et al., 2021), and cytotoxic activity (Ryu et al., 2015). Collectively, sesquiterpenes offer a wide potential for research and commercial applications.

Heterocyclic compounds often combine good activity, high selectivity, and low dosage, thus features attractive to new pesticide research (Jin and Zhang_, 2010; Wu et al., 2011; Wu et al., 2013). The presence of nitrogen in the molecule is usually accompanied by the emergence of new compound activities or the enhancement of the original activity characteristics of natural terpenoids (Lungu., 2015). Among them, 1,3,4-oxadiazole is a kind of heterocyclic compound with a variety of biological activities, and its derivatives show antiviral (Gan et al., 2017; He et al., 2021), antibacterial (Vasantha et al., 2019; Yu et al., 2021; Wang S. et al., 2021), antifungal (Wen et al., 2019; Wang X. et al., 2021) and insecticidal activity (Yang et al., 2020) in agricultural applications. Some also proved to be attractive anti-cancer (Kumar et al., 2009), anti-depressant (Ergun et al., 2010), anti-HIV (Parizadeh et al., 2018), and anti-inflammatory (Naseer et al., 2019) medicines. Additionally, the presence of alkyl groups on the oxadiazole nucleus increases their ability to penetrate active sites and enhance their biological activity (Vasantha et al., 2019).

**FIGURE 1:**
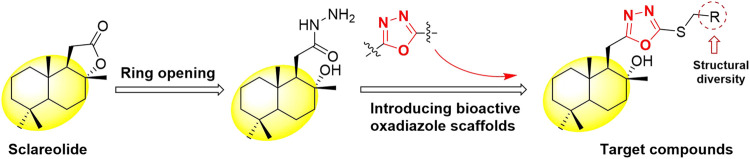
Design strategy of the target compounds.

In view of the above findings, as one of the most active research fields in natural product chemistry, sesquiterpenes can be derived from their skeletons to obtain active different compounds. In this study, using the principle of active substructure splicing, sclareolide was used as the lead compound and the active fragment of oxadiazole was introduced (Figure 1). A series of 1,3,4-oxadiazole contained sesquiterpene derivatives were synthesized and their biological activities were evaluated.

## Results and Discussion

### Antibacterial Activity *in Vitro*

The *in vitro* antibacterial activity of synthetic compounds **H1**–**H23** against *Xoo* and *Xa*c was tested by the turbidity method (Zhang et al., 2021). The preliminary biological activity results are shown in Table 1. The inhibitory activities of compounds **H4**, **H8**, **H15**, **H19**, and **H22** on *Xoo* were 64.5, 70.2, 69.5, 65.7, and 60.1% at 100 μg/ml, respectively, which were higher than that of thiodiazole copper (56.7%). The inhibitory effects of compounds **H4**, **H8**, **H14**, and **H19** on *Xac* at 100 μg/ml were 73.3, 65.1, 70.5, and 66.6%, respectively, which were better than that of bismerthiazol (64.6%).

**TABLE 1:** *In vitro* antibacterial activity of the target compounds against *Xoo* and *Xac* ^a^.

Compd	*Xoo* Inhibition rate (%)		*Xac* Inhibition rate (%)	
	100 μg/ml	50 μg/ml	100 μg/ml	50 μg/ml
**H1**	46.3 ± 4.6	23.8 ± 3.9	53.2 ± 3.4	45.3 ± 4.5
**H2**	20.9 ± 4.8	14.5 ± 3.4	49.6 ± 4.4	40.4 ± 2.9
**H3**	30.9 ± 3.2	22.7 ± 3.4	41.1 ± 2.7	39.1 ± 0.1
**H4**	64.5 ± 1.2	48.6 ± 2.3	73.3 ± 3.4	55.6 ± 3.1
**H5**	49.5 ± 3.0	22.9 ± 3.0	58.5 ± 1.8	36.1 ± 1.6
**H6**	21.1 ± 4.4	15.6 ± 1.6	46.7 ± 1.6	38.7 ± 4.0
**H7**	34.4 ± 3.2	33.7 ± 2.5	53.6 ± 1.9	34.5 ± 4.6
**H8**	70.2 ± 4.9	52.2 ± 1.1	65.1 ± 4.1	44.5 ± 3.1
**H9**	40.6 ± 3.4	25.5 ± 0.6	37.2 ± 4.3	24.1 ± 2.7
**H10**	16.6 ± 4.1	14.8 ± 0.6	47.7 ± 4.1	43.3 ± 3.9
**H11**	43.2 ± 3.2	19.0 ± 3.0	62.4 ± 3.2	45.9 ± 3.6
**H12**	24.6 ± 4.7	22.4 ± 3.4	58.7 ± 4.7	36.3 ± 1.5
**H13**	38.0 ± 1.1	22.5 ± 4.5	53.5 ± 3.4	35.4 ± 1.6
**H14**	38.0 ± 4.2	26.8 ± 3.2	70.5 ± 3.9	47.2 ± 0.5
**H15**	69.5 ± 4.5	42.3 ± 2.4	44.7 ± 1.1	40.6 ± 4.5
**H16**	28.6 ± 3.6	20.2 ± 1.1	62.8 ± 1.3	43.7 ± 4.9
**H17**	28.1 ± 1.1	25.4 ± 1.6	52.0 ± 1.2	46.5 ± 1.4
**H18**	50.2 ± 2.0	32.4 ± 3.3	50.5 ± 3.8	29.0 ± 2.8
**H19**	65.7 ± 4.7	47.1 ± 3.3	66.6 ± 1.5	48.4 ± 1.8
**H20**	53.1 ± 1.8	42.0 ± 3.9	42.5 ± 2.2	41.8 ± 2.3
**H21**	48.7 ± 4.6	47.8 ± 3.0	35.5 ± 2.8	34.1 ± 2.9
**H22**	60.1 ± 4.6	46.7 ± 2.2	48.4 ± 2.1	32.3 ± 4.4
**H23**	57.3 ± 2.2	38.7 ± 3.1	32.7 ± 3.4	30.9 ± 3.8
**BT** ^b^	73.5 ± 0.7	56.6 ± 4.7	64.6 ± 1.9	51.2 ± 1.4
**TC** ^b^	56.7 ± 3.8	48.5 ± 1.3	76.8 ± 0.7	65.2 ± 2.0

a Average of three replicates.

b The commercial agricultural antibacterial agents bismerthiazol. (BT) and thiodiazole copper (TC) were used as positive control.

The concentration values for 50% of maximal effect (EC_50_) of some compounds are shown in Table 2. The EC_50_ values of compounds **H8** and **H15** against *Xoo* were 43.3 and 43.4 μg/ml, respectively, which were close to bismerthiazol (41.8 μg/ml) and superior to that of thiodiazole copper (61.4 μg/ml). Compounds **H4** and **H14** had an inhibitory effect on *Xac*, with their EC_50_ values being 33.3 and 37.8 μg/ml, respectively, thus better than for bismerthiazol (38.2 μg/ml).

**TABLE 2:** Antibacterial activities of some target compounds against *Xoo* and *Xac in Vitro* ^a^.

Compd	*Xoo*			*Xac*		
	Regression equation	*R* ^2^	EC_50_ (*μg*/ml)	Regression equation	*R* ^2^	EC_50_ (*μg*/ml)
**H4**	y = 1.22x + 2.9	0.99	51.0 ± 3.3	y = 1.15x + 3.2	0.98	33.3 ± 1.0
**H8**	y = 1.20x + 3.0	0.97	43.3 ± 4.3	y = 0.74x + 3.7	0.96	42.7 ± 1.8
**H11**				y = 0.89x + 3.4	0.97	56.1 ± 3.5
**H12**				y = 0.78x + 3.5	0.94	74.5 ± 3.4
**H14**				y = 0.90x + 3.5	0.90	37.8 ± 3.1
**H15**	y = 1.54x + 2.4	0.92	43.4 ± 3.0			
**H16**				y = 0.83x + 3.6	0.98	43.8 ± 3.3
**H19**	y = 1.12x + 3.0	0.98	50.5 ± 4.0	y = 0.90x + 3.5	0.97	38.4 ± 4.6
**H22**	y = 1.00x + 3.1	0.94	74.6 ± 2.2			
**H23**	y = 1.41x + 2.5	0.99	51.4 ± 3.3			
**BT** ^b^	y = 1.63x + 2.3	0.98	41.8 ± 4.1	y = 0.76x + 3.7	0.98	38.2 ± 3.1
**TC** ^b^	y = 1.04x + 3.1	0.99	61.4 ± 1.8	y = 1.07x + 3.4	0.97	25.1 ± 1.9

a Average of three replicates.

b The commercial agricultural antibacterial agents bismerthiazol (BT) and thiodiazole copper (TC) were used as positive control.

### Antibacterial Activity *in Vivo*

**FIGURE 2:**
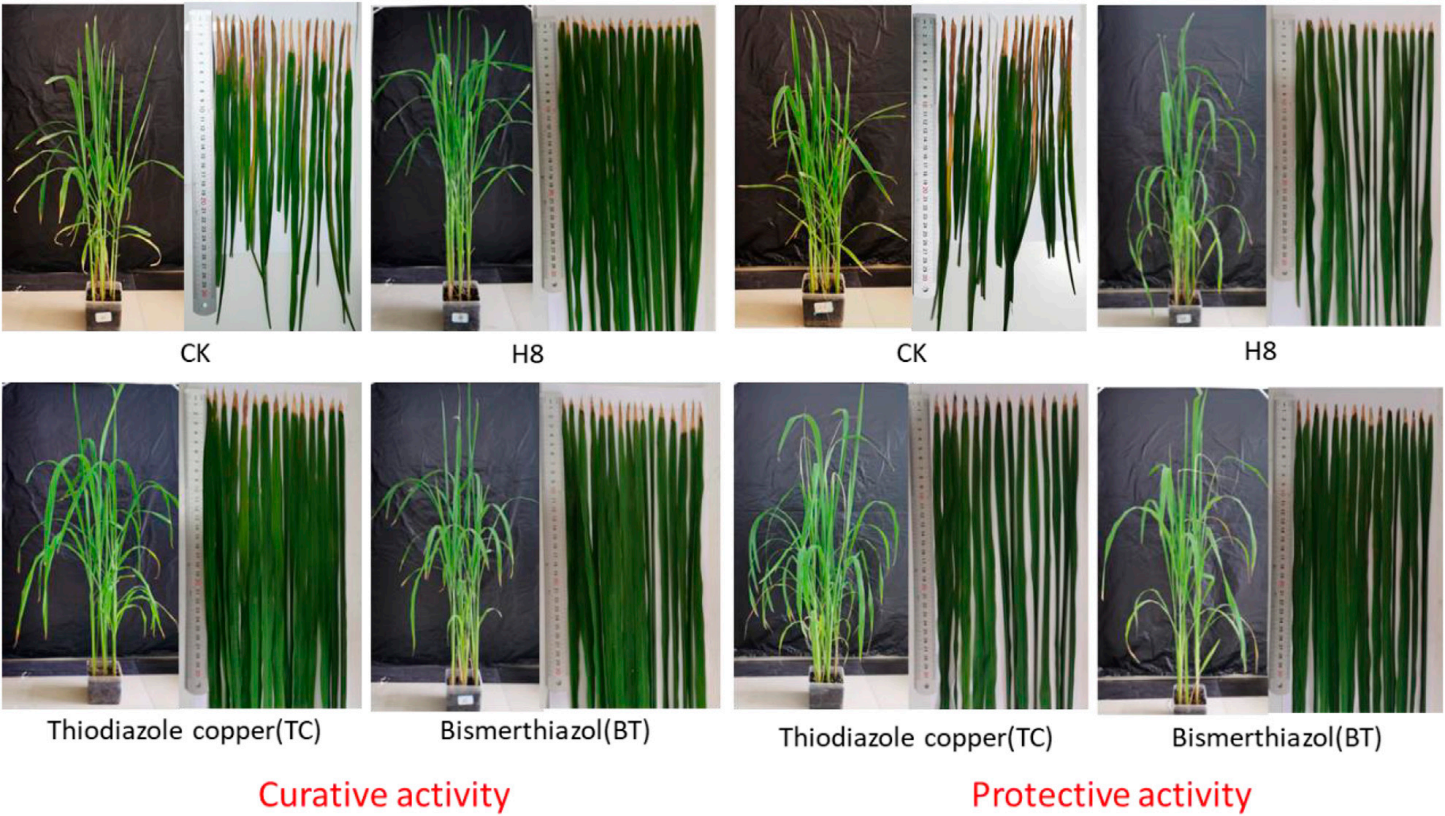
Curative and protective activities of compound **H8** against rice bacterial leaf blight under greenhouse conditions at 200 *μg*/ml, with BT and TC as the positive control agents.

To further verify the control effect of the compound on rice bacterial leaf blight, the *in vivo* antibacterial activity of compound **H8** was determined by the leaf-cutting method at 200 μg/ml (Zhang et al., 2021). The results are shown in Table 3, Table 4; Figure 2. The curative activity of compound **H8** was 51.9%, which was better than that of bismerthiazol (47.1%) and thiodiazole copper (46.1%). Concomitantly, the compound **H8** showed good protective activity of 49.3% compared to bismerthiazol (45.8%) and thiodiazole copper (43.7%).

**TABLE 3:** The curative activity of compound H8 against *Xanthomonas oryzae* pv. *oryzae in Vivo* at 200 μg/ml.

Treatment	14 Days after spraying		
	Morbidity (%)	Disease index (%)	Control efficiency (%)^a^
**H8**	100	41.7C	51.9A
**BT** ^b^	100	45.8B	47.1B
**TC** ^b^	100	46.6B	46.1B
**CK** ^c^	100	86.7A	

a Statistical analysis was conducted by the analysis of variance method under the conditions of equal variances assumed (*p* > 0.05) and equal variances not assumed (*p* < 0.05). Different uppercase letters indicate the values of curative activity with significant difference among different treatment groups at *p* < 0.05.

b Commercial bactericides bismerthiazol (BT) and thiodiazole copper (TC) were used as positive control agents.

c Negative control.

**TABLE 4:** The Protective activity of compound H8 against *Xanthomonas oryzae* pv. *oryzae in Vivo* at 200 μg/ml

Treatment	14 Days after spraying		
	Morbidity (%)	Disease index (%)	Control efficiency (%)^a^
**H8**	100	42.8D	49.3A
**BT** ^b^	100	45.8C	45.8B
**TC** ^b^	100	47.6B	43.7C
**CK** ^c^	100	84.6A	

a Statistical analysis was conducted by the analysis of variance method under the conditions of equal variances assumed (*p* > 0.05) and equal variances not assumed (*p* < 0.05). Different uppercase letters indicate the values of protective activity with significant difference among different treatment groups at *p* < 0.05.

b Commercial bactericides bismerthiazol (BT) and thiodiazole copper (TC) were used as positive control agents.

c Negative control.

### Anti-TMV Activity *in Vivo*

According to the classic literature method (Ren et al., 2020), the activity of the target compound **H1–H23** on TMV was tested. Preliminary bioactivity showed that most of the compounds exhibited a good inhibitory effect on TMV at 500 μg/ml. The results are shown in Table 5. Compared with ribavirin, most compounds had moderate to good activity. The curative activities of compounds **H8**, **H12**, **H16**, and **H19** were 68.3, 63.5, 67.5, and 63.3%, respectively, which were significantly higher than ribavirin (45.4%). Notably, the curative activity of compound **H9** was 77.5%, which was better than ningnanmycin (70.0%). The inactivation potency of compounds **H3**, **H4**, **H5**, **H9**, and **H16** were 81.7, 82.0, 87.5, 82.0, and 87.3%, respectively, which were higher than that of ribavirin (72.3%). It was worth noting that the inactivation potency of compound **H10** was 90.5%, which was slightly better than that of ningnanmycin (90.0%)

**TABLE 5:** Antiviral activities of target compounds against TMV *in Vivo* at 500 μg/mL^a^.

Compd	Curative activity^b^ (%)	Inactivation activity^b^ (%)
**H1**	59.1 ± 2.1	52.3 ± 3.8
**H2**	50.5 ± 4.5	63.2 ± 2.7
**H3**	58.7 ± 4.0	81.7 ± 2.3
**H4**	54.3 ± 2.8	82.0 ± 5.0
**H5**	53.2 ± 3.2	87.5 ± 0.5
**H6**	48.3 ± 5.0	71.0 ± 2.0
**H7**	55.5 ± 4.5	64.3 ± 5.0
**H8**	68.3 ± 4.1	45.7 ± 3.2
**H9**	77.5 ± 0.5	82.0 ± 3.7
**H10**	58.4 ± 1.8	90.5 ± 1.0
**H11**	55.4 ± 1.2	74.8 ± 1.5
**H12**	63.5 ± 2.5	36.3 ± 4.4
**H13**	51.3 ± 2.3	56.0 ± 4.0
**H14**	46.7 ± 1.6	72.5 ± 2.5
**H15**	49.9 ± 4.6	61.7 ± 2.6
**H16**	67.5 ± 4.5	87.3 ± 1.6
**H17**	46.9 ± 0.2	64.5 ± 0.5
**H18**	36.4 ± 0.7	61.5 ± 1.5
**H19**	63.3 ± 4.7	76.0 ± 2.0
**H20**	50.4 ± 1.2	54.5 ± 2.5
**H21**	57.8 ± 3.6	77.0 ± 2.0
**H22**	57.6 ± 0.7	75.0 ± 5.0
**H23**	59.6 ± 0.1	40.3 ± 1.8
**Ribavirin** ^c^	45.4 ± 1.6	72.3 ± 0.5
**Ningnanmycin** ^c^	70.0 ± 3.8	90.0 ± 1.5

a Average of three replicates.

b Concentration of compounds is 500 μg/ml

c Commercial antiviral agent ribavirin and ningnanmycin.

The EC_50_ values of some compounds were further tested, as shown in Table 6. The results indicated that the EC_50_ value of compound **H10** was 43.9 μg/ml, which was better than ningnanmycin (44.8 μg/ml).

**TABLE 6:** EC_50_ of inactivation activity of some target compounds against TMV.

Compd	Regression equation	*R* ^2^	EC_50_ ^a^
**H4**	y = 1.01x + 3.1	0.99	69.6 ± 4.6
**H5**	y = 1.18x + 2.9	0.96	58.9 ± 3.5
**H9**	y = 1.02x + 3.1	0.99	69.4 ± 4.5
**H10**	y = 1.23x + 2.9	0.99	43.9 ± 4.2
**H16**	y = 1.15x + 2.9	0.97	60.5 ± 2.9
**Ningnanmycin** ^b^	y = 1.22x + 2.9	0.99	44.8 ± 2.8

a Average of three replicates.

b Ningnanmycin was used as the control.

### Molecular Docking and MD Simulation

**FIGURE 3:**
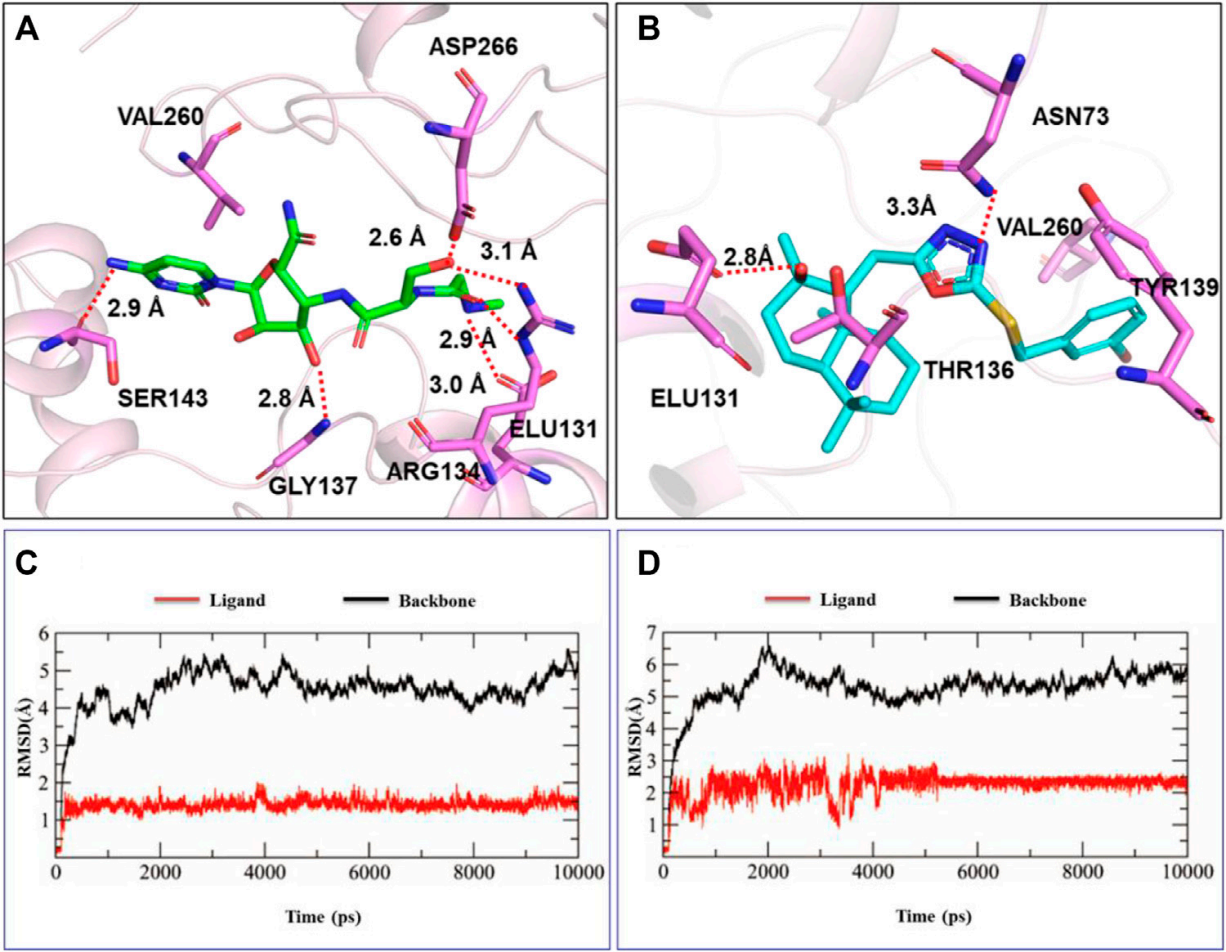
Molecule docking and MD simulation studies: **(A)** molecule docking of ningnanmycin, **(B)** molecule docking of compound **H10**, **(C)** MD simulation of ningnanmycin, **(D)** MD simulation of compound **H10**.

TMV coat protein (TMV-CP) plays an important role in the replication and assembly of plant viruses. Our goal was to investigate the interaction between active target compounds and TMV-CP. The binding method of ligand molecules (compound **H10** and ningnanmycin) and TMV-CP (PDB 97 code: 1EI7) was explored through molecular docking, and the results are shown in Figures 3A,B. Compound **H10** had a strong affinity for TMV-CP, with a binding energy of -8.88 kcal/mol, while that of ningnanmycin was 6.35 kcal/mol. The hydroxyl oxygen atom of compound **H10** formed a strong hydrogen bond with ASN73 and ELU131 (the bond length is 3.1Å and 2.8Å, respectively), and the residue ELU131 can also be seen in ningnanmycin. Compound **H10** had two hydrophobic interactions with amino acid residues TYR139 and THR136 in addition to interacted with VAL260 via hydrophobic bonds like ningnanmycin.

The stability and interaction mode of the ligand molecule and TMV-CP under the simulated conditions were further studied through molecular dynamics (MD) simulation, and the root-mean-square deviation (RMSD) of the atom and its initial position was measured (Figures 3C,D). Due to the significant interaction between the ligand and the binding site, the difference in energy characteristics results in a stable conformation and strong binding. Therefore, the biological activity can be influenced by optimizing the structure of the compound, and the properties of inhibiting TMV can be explored.

### Structure-Activity Relationship Analysis

The preliminary structure-activity relationship showed that the different substituents R of sesquiterpene derivatives had a great influence on *Xoo*, *Xa*c, and TMV. According to Table 1, when there are electron-withdrawing F, Cl or F, Br atoms on the benzene ring at the same time, the activity of the compound against *Xoo* is reduced: **H4** (R = Ph) > **H21** (R = 4-Br-2-F-Ph) > **H1** (R = 2-Cl-5-F-Ph) > **H9** (R = 2-Br-5-F-Ph) > **H16** (R = 2-Br-4-F-Ph) > **H17** (R = 3-Cl-2-F-Ph) > **H6** (R = 2-Cl-4-F-Ph). The position of difluoro substitution on the aromatic ring also had an effect on the activity of *Xac*: **H8** (R = 2,4-di-F-Ph) > **H12** (R = 2,3-di-F-Ph) > **H5** (R = 2,6-di-F-Ph) > **H13** (R = 3,5-di-F-Ph) > **H2** (R = 2,5-di-F-Ph). As shown in Table 5, introduction of different groups at the 4-position of the aromatic ring, altered the compounds’ curative activities against TMV, with the electron-donating group having improved activity over the electron-withdrawing group: H**19** (R = 4-OCH_3_-Ph) > **H23** (R = 4-NO_2_-Ph) > **H3** (R = 4-CF_3_-Ph) > **H22** (R = 4-OCF_3_-Ph) > **H14** (R = 4-Cl-Ph) > **H18** (R = 4-Br-2-F-Ph). The type and position of a single halogen atom on the benzene ring and heterocyclic ring may affect the inactivation potency of the compound: **H10** (R = 3-Br-Ph) > **H11** (R = 4-Cl-Py) > **H14** (R = 4-Cl-Ph) > **H15** (R = 2-Cl-Ph) > **H18** (R = 4-Br-2-F-Ph) > **H20** (R = 5-Cl-thiazol).

## Materials and Methods

### General Information

Melting points (uncorrected) of the synthetic compounds were determined using the XT-4 micro melting point instrument (Beijing Tech Instrument Co., China). All of the reactions were performed using a magnetic stir bar, followed by thin-layer chromatography (TLC) on silica gel GF254 and identified by UV. The ^1^H, ^13^C, and ^19^F nuclear magnetic resonance (NMR) spectra were obtained with AVANCE III HD 400 MHz or 500 MHz (Bruker Corporation, Switzerland) system in CDCl_3_, and used TMS as an internal standard at room temperature. High-resolution mass spectrometer (HRMS) data was conducted using an Orbitrap LC-MS instrument (Q-Exative, Thermo Scientific™, United States). All reagents and solvents were purchased from commercial suppliers and were not subjected to further purification and drying.

### Chemistry

**SCHEME 1:**
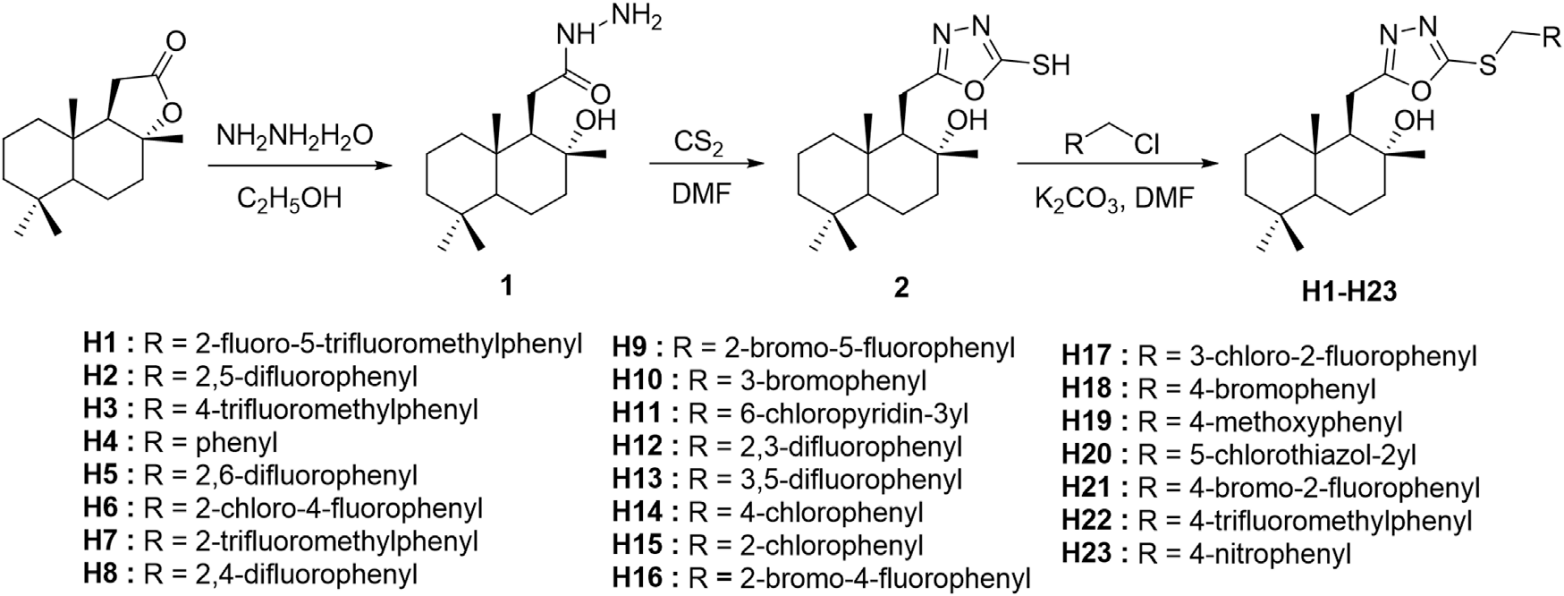
The synthetic route of the target compounds **H1-H23**.

According to the synthetic route shown in Scheme 1, the target compounds **H1–H23** were obtained. The natural product sclareolide was used as raw material to produce hydrazide intermediate **1** by hydrazinolysis reaction with hydrazine hydrate under weakly alkaline conditions. Intermediate **1** continues to form a closed loop with carbon disulfide under reflux to obtain oxadiazole intermediate **2**. Then, under the alkaline condition in the presence of anhydrous potassium carbonate, intermediate **2** reacts with different substituted benzyl halides to synthesize the target compounds **H1–H23**.

### Synthesis

#### General Procedure for the Preparation of the Intermediates 1 and 2

As shown in Scheme 1, the previously published methods were used (Zhang et al., 2013; Mishra et al., 2017). The raw material sclareolide (500 mg, 1 mol) was dissolved in a round bottom flask with EtOH, and hydrazine hydrate (1 ml, 11 mol) was added and stirred at room temperature for 2 h. After the reaction was completed, an appropriate amount of water was added to the system, and the precipitate was collected by filtration to obtain Intermediate **1**. Subsequently, Intermediate **1** (300 mg, 1 mol) was dissolved in DMF and stirred for 30 min, carbon disulfide (743 mg, 5 mol) was slowly added and refluxed for 6–8 h. The reaction mixture was diluted with water and extracted with ethyl acetate. The organic layer was dried over NaSO_4_ and concentrated under vacuum. The residue was purified by silica gel chromatography with petroleum ether/ethyl acetate (8:1) concentrated eluent to obtain Intermediate **2**.

### General Procedures for the Preparation of Target Compounds H1-H23

According to the published method (Wang et al., 2019), Intermediate **2** (200mg, 1 mol) and potassium carbonate (107mg, 1.2 mol) were dissolved in a round bottom flask with DMF and stirred for 30 min. Different substituted benzyl halides were added and reacted at room temperature for 6–7 h. An appropriate amount of water was added to the reaction mixture to filter the residue. The crude product was subjected to column chromatography with petroleum ether/ethyl acetate (5:1) to extract target compounds **H1-H23**.

The structures of synthesized compounds **H1-H23** were confirmed by ^1^H NMR, ^13^C NMR, ^19^F NMR, and HRMS.

**(1*R*,2*R*,8a*S*)-1-((5-((2-fluoro-5-(trifluoromethyl)benzyl**)**thio)-1,3,4-oxadiazol-2-yl)methyl**)**-2,5,5,8a-tetramethyldecahydronaphthalen-2-ol (H1).** Yield 95%; White solid; m. p.75–76°C. ^1^H NMR (400 MHz, CDCl_3_) δ 7.81 (dd, *J* = 6.8, 2.1 Hz, 1H), 7.61–7.54 (m, 1H), 7.19 (t, *J* = 8.9 Hz, 1H), 4.47 (s, 2H), 2.90 (ddd, *J* = 76.1, 16.3, 5.7 Hz, 2H), 1.95–1.89 (m, 2H), 1.74–1.51 (m, 4H), 1.46 (d, *J* = 3.7 Hz, 1H), 1.40–1.30 (m, 4H), 1.21 (s, 3H), 1.00 (dd, *J* = 12.1, 2.2 Hz, 1H), 0.88 (s, 3H), 0.87 (s, 3H), 0.80 (s, 3H). ^13^C NMR (100 MHz, CDCl_3_) δ 170.5, 162.7 (d, *J* = 254.3 Hz), 162.3, 128.8 (d, *J* = 8.0 Hz), 127.4 (d, *J* = 3.7 Hz), 127.3 (d, *J* = 3.7 Hz), 124.6 (d, *J* = 15.6 Hz), 123.5 (d, *J* = 272.0 Hz), 116.2 (d, *J* = 22.7 Hz), 73.2, 59.0, 55.7, 44.5, 41.5, 39.3, 38.8, 33.3, 33.2, 29.5, 23.3, 21.4, 21.1, 20.4, 18.3, 15.1.^19^F NMR (376 MHz, CDCl_3_) δ -61.91, -110.96. HRMS (ESI+) m/z Calcd for C_25_H_33_F_4_SN_2_O_2_ [M + H]^+^ 501.21934; Found 501.21936.

**(1*R*,2*R*,8a*S*)-1-((5-((2,5-difluorobenzyl)thio**)**-1,3,4-oxadiazol-2-yl)methyl**)**-2,5,5,8a-tetramethyldecahydronaphthalen-2-ol (H2).** Yield 63%; White solid; m. p.84–86°C. ^1^H NMR (400 MHz, CDCl_3_) δ 7.26–7.22 (m, 1H), 7.04–7.00 (m, 1H), 6.98–6.95 (m, 1H), 4.40 (s, 2H), 2.91 (ddd, *J* = 76.7, 16.3, 5.6 Hz, 2H), 1.96–1.91 (m, 2H), 1.75–1.67 (m, 2H), 1.54–1.49 (m, 2H), 1.47–1.43 (m, 1H), 1.40–1.30 (m, 4H), 1.21 (s, 3H), 1.00 (dd, *J* = 12.1, 2.2 Hz, 1H), 0.88 (s, 3H), 0.87 (s, 3H), 0.80 (s, 3H). ^13^C NMR (100 MHz, CDCl_3_) δ 170.5, 162.6, 158.3 (d, *J* = 245.5 Hz), 156.8 (d, *J* = 246.6 Hz), 125.0 (dd, *J* = 17.1, 8.1 Hz), 117.7 (dd, *J* = 24.7, 3.6 Hz), 116.6 (dd, *J* = 21.2, 5.5 Hz), 116.3 (dd, *J* = 20.9, 5.4 Hz), 73.2, 59.0, 55.7, 44.5, 41.5, 39.3, 38.8, 33.4, 33.2, 29.7, 23.3, 21.4, 21.1, 20.4, 18.3, 15.1.^19^F NMR (376 MHz, CDCl_3_) δ -118.08, -122.78. HRMS (ESI+) m/z Calcd for C_24_H_33_F_2_SN_2_O_2_ [M + H]^+^ 451.22253; Found 451.22229.

**(1*R*,2*R*,8a*S*)-2,5,5,8a-tetramethyl-1-((5-((4-(trifluoromethyl)benzyl**)**thio)-1,3,4-oxadiazol-2-yl)methyl**)**decahydronaphthalen-2-ol (H3).** Yield 64%; White solid; m. p.81–83°C. ^1^H NMR (400 MHz, CDCl_3_) δ 7.58 (d, *J* = 8.5 Hz, 2H), 7.55 (d, *J* = 8.4 Hz, 2H), 4.44 (s, 2H), 2.89 (ddd, *J* = 75.7, 16.2, 5.7 Hz, 2H), 1.93–1.87 (m, 1H), 1.73–1.58 (m, 4H), 1.44 (ddt, *J* = 10.0, 6.8, 5.2 Hz, 4H), 1.33 (ddd, *J* = 13.5, 6.2, 1.3 Hz, 2H), 1.21 (s, 3H), 0.99 (dd, *J* = 12.1, 2.2 Hz, 1H), 0.88 (s, 3H), 0.86 (s, 3H), 0.80 (s, 3H). ^13^C NMR (100 MHz, CDCl_3_) δ 170.4, 162.5, 140.1 (d, *J* = 1.3 Hz), 130.1 (d, *J* = 32.6 Hz), 129.4, 129.2, 125.6 (d, *J* = 3.8 Hz), 125.6 (d, *J* = 11.2 Hz), 123.9 (d, *J* = 272.3 Hz), 73.2, 59.1, 55.8, 44.5, 41.5, 39.4, 38.8, 36.0, 33.3, 33.2, 23.3, 21.4, 21.1, 20.4, 18.3, 15.1. HRMS (ESI+) m/z Calcd for C_25_H_34_F_3_SN_2_O_2_ [M + H]^+^ 483.22876; Found 483.22870.

**(1*R*,2*R*,8a*S*)-1-((5-(benzylthio)-1,3,4-oxadiazol-2-yl)methyl**)**-2,5,5,8a-tetramethyldecahydronaphthalen-2-ol (H4).** Yield 91%; Pink solid; m. p.78–80°C. ^1^H NMR (400 MHz, CDCl_3_) δ 7.41 (dd, *J* = 8.0, 1.4 Hz, 2H), 7.35–7.30 (m, 3H), 4.42 (s, 2H), 2.90 (ddd, *J* = 77.6, 16.2, 5.6 Hz, 2H), 1.91 (ddd, *J* = 15.1, 9.0, 4.4 Hz, 2H), 1.73–1.64 (m, 1H), 1.59–1.47 (m, 2H), 1.43–1.34 (m, 4H), 1.32–1.23 (m, 2H), 1.20 (s, 3H), 0.99 (dd, *J* = 12.1, 2.2 Hz, 1H), 0.88 (s, 3H), 0.87 (s, 3H), 0.80 (s, 3H). ^13^C NMR (100 MHz, CDCl_3_) δ 170.1, 163.1, 135.7, 129.1, 128.7, 128.0, 73.2, 59.1, 55.7, 44.5, 41.5, 39.3, 38.8, 36.8, 33.3, 33.2, 23.3, 21.4, 21.1, 20.4, 18.3, 15.1. HRMS (ESI+) m/z Calcd for C_24_H_35_SN_2_O_2_ [M + H]^+^ 415.24138; Found 415.24130.

**(1*R*,2*R*,8a*S*)-1-((5-((2,6-difluorobenzyl)thio**)**-1,3,4-oxadiazol-2-yl)methyl**)**-2,5,5,8a-tetramethyldecahydronaphthalen-2-ol (H5).** Yield 84%; Pink solid; m. p.87–88°C. ^1^H NMR (400 MHz, CDCl_3_) δ 7.21–7.16 (m, 1H), 6.88–6.83 (m, 2H), 4.24 (s, 2H), 2.49–2.40 (m, 2H), 2.08–2.04 (m, 1H), 1.89–1.76 (m, 2H), 1.67–1.59 (m, 2H), 1.48–1.39 (m, 4H), 1.38–1.28 (m, 2H), 1.25 (s, 3H), 1.02 (d, *J* = 2.6 Hz, 1H), 0.88 (s, 3H), 0.87 (s, 3H), 0.82 (s, 3H). ^13^C NMR (100 MHz, CDCl_3_) δ 168.3, 161.5 (d, *J* = 250.1 Hz), 161.4 (d, *J* = 250.1 Hz), 154.5, 128.9 (d, *J* = 10.5 Hz), 113.9 (d, *J* = 19.4 Hz), 111.2 (d, *J* = 25.1 Hz), 111.2 (d, *J* = 12.8 Hz), 73.2, 59.1, 56.6, 42.1, 39.4, 38.6, 36.2, 33.3, 33.1, 26.3, 21.9, 20.9, 20.7, 18.1, 15.1.^19^F NMR (376 MHz, CDCl_3_) δ -113.32, -113.49. HRMS (ESI+) m/z Calcd for C_24_H_33_F_2_SN_2_O_2_ [M + H]^+^ 421.22253; Found 421.22253.

**(1*R*,2*R*,8a*S*)-1-((5-((2-chloro-4-fluorobenzyl)thio**)**-1,3,4-oxadiazol-2-yl)methyl**)**-2,5,5,8a-tetramethyldecahydronaphthalen-2-ol (H6).** Yield 84%; White solid; m. p.86–87°C. ^1^H NMR (400 MHz, CDCl_3_) δ 7.51 (dd, *J* = 8.3, 6.3 Hz, 1H), 7.09 (dd, *J* = 8.5, 2.6 Hz, 1H), 6.90 (td, *J* = 8.4, 2.6 Hz, 1H), 4.21 (s, 2H), 2.45 (t, *J* = 9.3 Hz, 2H), 2.08–2.04 (m, 1H), 1.83 (ddd, *J* = 30.2, 10.4, 5.2 Hz, 2H), 1.67–1.58 (m, 2H), 1.47–1.39 (m, 4H), 1.32 (m, 2H), 1.24 (s, 3H), 1.01 (d, *J* = 2.3 Hz, 2H), 0.88 (s, 3H), 0.87 (s, 3H). ^13^C NMR (100 MHz, CDCl_3_) δ 170.4, 162.9, 162.2 (d, *J* = 250.8 Hz), 135.0 (d, *J* = 10.4 Hz), 132.6 (d, *J* = 8.9 Hz), 129.9 (d, *J* = 3.7 Hz), 117.1 (d, *J* = 24.9 Hz), 114.3 (d, *J* = 21.1 Hz), 73.2, 59.0, 55.7, 44.4, 41.5, 39.3, 38.8, 33.8, 33.3, 33.2, 23.3, 21.4, 21.1, 20.4, 18.3, 15.1.^19^F NMR (376 MHz, CDCl_3_) δ -111.21. HRMS (ESI+) m/z Calcd for C_24_H_33_FClSN_2_O_2_ [M + H]^+^ 467.19298; Found 467.19293.

**(1*R*,2*R*,8a*S*)-2,5,5,8a-tetramethyl-1-((5-((2-(trifluoromethyl)benzyl**)**thio)-1,3,4-oxadiazol-2-yl)methyl**)**decahydronaphthalen-2-ol (H7).** Yield 98%; Pink solid; m. p.115–117°C. ^1^H NMR (400 MHz, CDCl_3_) δ 7.67 (d, *J* = 7.6 Hz, 1H), 7.60 (d, *J* = 7.7 Hz, 1H), 7.45 (dd, *J* = 14.3, 6.6 Hz, 1H), 7.37–7.29 (m, 1H), 4.35 (s, 2H), 2.49–2.40 (m, 2H), 2.08–2.03 (m, 1H), 1.95–1.84 (m, 2H), 1.67–1.61 (m, 2H), 1.47–1.39 (m, 4H), 1.37–1.29 (m, 2H), 1.25 (s, 3H), 1.00 (dd, *J* = 12.5, 2.7 Hz, 1H), 0.88 (s, 3H), 0.87 (s, 3H), 0.82 (s, 3H). ^13^C NMR (100 MHz, CDCl_3_) δ 168.6, 154.6, 137.4, 132.2 (d, *J* = 24.9 Hz), 131.9 (d, *J* = 5.0 Hz), 128.2, 127.1, 125.7 (d, *J* = 5.8 Hz), 124.3 (d, *J* = 274.0 Hz), 73.2, 59.1, 56.6, 44.5, 42.1, 39.4, 38.6, 36.2, 33.3, 33.1, 26.3, 21.9, 20.9, 20.7, 18.1, 15.1.^19^F NMR (376 MHz, CDCl_3_) δ -59.24. HRMS (ESI+) m/z Calcd for C_25_H_34_F_3_SN_2_O_2_ [M + H]^+^ 483.22876; Found 483.22855.

**(1*R*,2*R*,8a*S*)-1-((5-((2,4-difluorobenzyl)thio**)**-1,3,4-oxadiazol-2-yl)methyl**)**-2,5,5,8a-tetramethyldecahydronaphthalen-2-ol (H8).** Yield 72%; White solid; m. p.93–95°C. ^1^H NMR (400 MHz, CDCl_3_) δ 7.44 (dd, *J* = 15.4, 8.5 Hz, 1H), 6.83–6.78 (m, 1H), 6.77–6.72 (m, 1H), 4.12 (s, 2H), 2.50–2.40 (m, 4H), 2.10–1.73 (m, 1H), 1.49–1.40 (m, 4H), 1.38–1.28 (m, 2H), 1.25 (s, 3H), 1.02 (d, *J* = 2.4 Hz, 1H), 0.88 (s, 3H), 0.87 (s, 3H), 0.83 (s, 3H). ^13^C NMR (126 MHz, CDCl_3_) δ 170.5, 162.8 (d, *J* = 249.6 Hz), 162.8, 161.1 (d, *J* = 250.7 Hz), 132.3 (dd, *J* = 9.6, 4.8 Hz), 119.4 (dd, *J* = 14.5, 3.5 Hz), 111.5 (dd, *J* = 21.0, 3.5 Hz), 104.2 (d, *J* = 25.4 Hz), 73.3, 59.1, 55.8, 44.6, 41.6, 39.4, 38.8, 33.4, 33.3, 29.5, 23.3, 21.5, 21.2, 20.4, 18.4, 15.2.^19^F NMR (376 MHz, CDCl_3_) δ -109.30, -112.11. HRMS (ESI+) m/z Calcd for C_24_H_32_F_2_SN_2_O_2_Na [M + Na]^+^ 473.20448; Found 473.20499.

**(1*R*,2*R*,8a*S*)-1-((5-((2-bromo-5-fluorobenzyl)thio**)**-1,3,4-oxadiazol-2-yl)methyl**)**-2,5,5,8a-tetramethyldecahydronaphthalen-2-ol (H9).** Yield 84%; White solid; m. p.120–122°C. ^1^H NMR (400 MHz, CDCl_3_) δ 7.47 (dd, *J* = 8.8, 5.3 Hz, 1H), 7.30 (dd, *J* = 9.3, 3.0 Hz, 1H), 6.82 (td, *J* = 8.4, 3.1 Hz, 1H), 4.22 (s, 2H), 2.56–2.39 (m, 2H), 2.10–1.74 (m, 4H), 1.61 (dd, *J* = 11.6, 4.2 Hz, 1H), 1.44 (ddd, *J* = 20.8, 12.2, 5.6 Hz, 4H), 1.32 (ddd, *J* = 16.5, 10.4, 3.4 Hz, 2H), 1.25 (s, 3H), 1.02 (d, *J* = 2.5 Hz, 1H), 0.88 (s, 3H), 0.87 (s, 3H), 0.83 (s, 3H). ^13^C NMR (100 MHz, CDCl_3_) δ 168.3, 161.8 (d, *J* = 246.9 Hz), 154.7, 140.3 (d, *J* = 8.0 Hz), 133.6 (d, *J* = 8.1 Hz), 118.6 (d, *J* = 3.6 Hz), 118.2 (d, *J* = 23.4 Hz), 115.8 (d, *J* = 23.9 Hz), 74.1, 59.1, 56.6, 42.1, 39.4, 38.6, 36.2, 33.3, 33.1, 26.3, 21.9, 20.9, 20.7, 18.1, 15.1.^19^F NMR (376 MHz, CDCl_3_) δ -114.65. HRMS (ESI+) m/z Calcd for C_24_H_32_FBrSN_2_O_2_Na [M + Na]^+^ 533.12441; Found 533.12457.

**(1*R*,2*R*,8a*S*)-1-((5-((3-bromobenzyl)thio**)**-1,3,4-oxadiazol-2-yl)methyl**)**-2,5,5,8a-tetramethyldecahydronaphthalen-2-ol (H10).** Yield 83%; White solid; m. p.68–70°C.

^1^H NMR (400 MHz, CDCl_3_) δ 7.57 (t, *J* = 1.7 Hz, 1H), 7.44–7.40 (m, 1H), 7.36 (d, *J* = 7.8 Hz, 1H), 7.20 (t, *J* = 7.8 Hz, 1H), 4.37 (s, 2H), 3.05–2.72 (m, 2H), 1.96–1.79 (m, 4H), 1.73–1.66 (m, 1H), 1.56–1.42 (m, 4H), 1.28 (ddd, *J* = 13.4, 6.6, 3.5 Hz, 2H), 1.20 (s, 3H), 1.01 (d, *J* = 2.2 Hz, 1H), 0.88 (s, 3H), 0.86 (s, 3H), 0.80 (s, 3H). ^13^C NMR (100 MHz, CDCl_3_) δ 170.3, 162.6, 138.1, 132.0, 131.1, 130.3, 127.8, 122.6, 73.2, 59.0, 55.7, 44.5, 41.5, 39.3, 38.8, 36.0, 33.4, 33.2, 23.3, 21.4, 21.1, 20.4, 18.3, 15.1. HRMS (ESI+) m/z Calcd for C_24_H_34_BrSN_2_O_2_ [M + H]^+^ 493.15189; Found 493.15204.

**(1*R*,2*R*,8a*S*)-1-((5-(((6-chloropyridin-3-yl)methyl**)**thio)-1,3,4-oxadiazol-2-yl)methyl**)**-2,5,5,8a-tetramethyldecahydronaphthalen-2-ol (H11).** Yield 72%; White solid; m. p.102–104°C. ^1^H NMR (400 MHz, CDCl_3_) δ 8.44–8.36 (m, 2H), 7.69 (dd, *J* = 8.2, 2.3 Hz, 1H), 4.07 (s, 2H), 2.55–2.39 (m, 2H), 2.06 (dt, *J* = 11.6, 3.1 Hz, 1H), 1.88–1.74 (m, 2H), 1.68–1.60 (m, 2H), 1.44 (dt, *J* = 20.2, 5.8 Hz, 4H), 1.32 (ddd, *J* = 16.5, 11.1, 3.4 Hz, 2H), 1.26 (s, 3H), 1.02 (d, *J* = 2.7 Hz, 1H), 0.88 (s, 6H), 0.83 (s, 3H). ^13^C NMR (100 MHz, CDCl_3_) δ 168.0, 154.9, 149.9, 139.5, 133.8, 123.9, 89.0, 59.1, 56.6, 42.1, 39.4, 38.6, 36.2, 33.3, 33.0, 29.4, 26.4, 21.9, 20.9, 20.7, 18.1, 15.1. HRMS (ESI+) m/z Calcd for C_23_H_31_ClSN_3_O_2_ [M-H]^-^ 448.18200; Found 448.18344.

**(1*R*,2*R*,8a*S*)-1-((5-((2,3-difluorobenzyl)thio**)**-1,3,4-oxadiazol-2-yl)methyl**)**-2,5,5,8a-tetramethyldecahydronaphthalen-2-olH-12 (H12).** Yield 67%; White solid; m. p.99–100°C. ^1^H NMR (400 MHz, CDCl_3_) δ 7.19 (ddd, *J* = 8.8, 5.8, 3.1 Hz, 1H), 6.95 (td, *J* = 9.0, 4.5 Hz, 1H), 6.92–6.84 (m, 1H), 4.12 (s, 2H), 2.53–2.40 (m, 2H), 2.06 (dt, *J* = 11.6, 3.2 Hz, 1H), 1.87 (ddd, *J* = 14.0, 6.8, 3.2 Hz, 2H), 1.78 (dd, *J* = 13.5, 7.2 Hz, 2H), 1.69–1.59 (m, 2H), 1.49–1.32 (m, 4H), 1.25 (s, 3H), 1.02 (d, *J* = 2.5 Hz, 1H), 0.88 (s, 3H), 0.88 (s, 3H), 0.83 (s, 3H). ^13^C NMR (100 MHz, CDCl_3_) δ 168.3, 159.6, 156.8 (d, *J* = 243.0 Hz), 155.9 (d, *J* = 255.4 Hz), δ 127.6 (dd, *J* = 17.6, 8.1 Hz), 117.5 (dd, *J* = 24.4, 3.4 Hz), 116.0 (dd, *J* = 24.6, 8.7 Hz), 115.0 (dd, *J* = 23.9, 8.5 Hz), 88.8, 59.1, 56.6, 42.1, 39.4, 38.6, 36.2, 33.3, 33.1, 26.3, 21.9, 20.9, 20.7, 18.1, 15.1.^19^F NMR (376 MHz, CDCl_3_) δ -119.09, -123.36. HRMS (ESI+) m/z Calcd for C_24_H_33_F_2_SN_2_O_2_ [M + H]^+^ 451.22253; Found 451.22275.

**(1*R*,2*R*,8a*S*)-1-((5-((3,5-difluorobenzyl)thio**)**-1,3,4-oxadiazol-2-yl)methyl**)**-2,5,5,8a-tetramethyldecahydronaphthalen-2-ol (H13).** Yield 60%; White solid; m. p.86–88°C. ^1^H NMR (400 MHz, CDCl_3_) δ 6.90 (dd, *J* = 8.2, 2.2 Hz, 2H), 6.65 (tt, *J* = 9.0, 2.3 Hz, 1H), 4.08 (s, 2H), 2.54–2.41 (m, 2H), 2.07 (dt, *J* = 11.8, 3.2 Hz, 1H), 1.91–1.79 (m, 2H), 1.65–1.58 (m, 2H), 1.49–1.38 (m, 4H), 1.39–1.27 (m, 2H), 1.26 (s, 3H), 1.02 (d, *J* = 2.7 Hz, 1H), 0.88 (s, 6H), 0.83 (s, 3H). ^13^C NMR (100 MHz, CDCl_3_) δ 168.1, 162.9 (d, *J* = 248.2 Hz), 162.7 (d, *J* = 248.1 Hz), 154.8, 142.6 (d, *J* = 9.0 Hz), 111.8 (d, *J* = 11.7 Hz), 111.8 (d, *J* = 25.4 Hz), 102.3 (d, *J* = 25.3 Hz), 88.9, 59.1, 56.6, 42.1, 39.4, 38.6, 36.2, 33.3, 33.1, 26.4, 21.9, 20.9, 20.7, 18.1, 15.1.^19^F NMR (376 MHz, CDCl_3_) δ -110.18, -110.18. HRMS (ESI+) m/z Calcd for C_24_H_33_F_2_SN_2_O_2_ [M + H]^+^ 451.22253; Found 451.22287.

**(1*R*,2*R*,8a*S*)-1-((5-((4-chlorobenzyl)thio**)**-1,3,4-oxadiazol-2-yl)methyl**)**-2,5,5,8a-tetramethyldecahydronaphthalen-2-ol (H14).** Yield 65%; White solid; m. p.95–96°C.

^1^H NMR (500 MHz, CDCl_3_) δ 7.38–7.34 (m, 2H), 7.32–7.28 (m, 2H), 4.37 (s, 2H), 2.89 (ddd, *J* = 21.7, 16.3, 5.7 Hz, 2H), 1.99–1.84 (m, 2H), 1.74–1.66 (m, 2H), 1.56–1.48 (m, 2H), 1.46–1.34 (m, 4H), 1.26 (dd, *J* = 13.3, 3.2 Hz, 1H), 1.20 (s, 3H), 0.99 (dd, *J* = 12.2, 2.1 Hz, 1H), 0.88 (s, 3H), 0.86 (s, 3H), 0.80 (s, 3H). ^13^C NMR (126 MHz, CDCl_3_) δ 170.4, 162.8, 134.5, 133.9, 130.5, 128.9, 73.3, 59.1, 55.8, 44.6, 41.6, 39.4, 38.8, 36.1, 33.4, 33.3, 23.4, 21.5, 21.2, 20.4, 18.4, 15.2. HRMS (ESI+) m/z Calcd for C_24_H_34_ClSN_2_O_2_ [M + H]^+^ 449.20240; Found 449.20154.

**(1*R*,2*R*,8a*S*)-1-((5-((2-chlorobenzyl)thio**)**-1,3,4-oxadiazol-2-yl)methyl**)**-2,5,5,8a-tetramethyldecahydronaphthalen-2-ol (H15).** Yield 74%; White solid; m. p.87–89°C.

^1^H NMR (500 MHz, CDCl_3_) δ 7.56 (dd, *J* = 7.3, 1.9 Hz, 1H), 7.39 (dd, *J* = 7.7, 1.3 Hz, 1H), 7.26–7.18 (m, 2H), 4.53 (s, 2H), 2.89 (ddd, *J* = 96.9, 16.3, 5.5 Hz, 2H), 1.92 (ddd, *J* = 14.8, 8.8, 4.3 Hz, 2H), 1.75–1.67 (m, 2H), 1.55–1.47 (m, 2H), 1.44–1.33 (m, 4H), 1.26 (dd, *J* = 13.5, 3.5 Hz, 1H), 1.20 (s, 3H), 0.99 (dd, *J* = 12.1, 2.0 Hz, 1H), 0.88 (s, 3H), 0.86 (s, 3H), 0.80 (s, 3H). ^13^C NMR (126 MHz, CDCl_3_) δ 170.4, 163.1, 134.3, 133.9, 131.5, 129.8, 129.6, 127.1, 73.3, 59.1, 55.8, 44.6, 41.6, 39.4, 38.9, 34.61, 33.4, 33.3, 23.3, 21.5, 21.2, 20.4, 18.4, 15.2. HRMS (ESI+) m/z Calcd for C_24_H_34_ClSN_2_O_2_ [M + H]^+^ 449.20240; Found 449.20117.

**(1*R*,2*R*,8a*S*)-1-((5-((2-bromo-4-fluorobenzyl)thio**)**-1,3,4-oxadiazol-2-yl)methyl**)**-2,5,5,8a-tetramethyldecahydronaphthalen-2-ol (H16).** Yield 70%; White solid; m. p.92–94°C. ^1^H NMR (500 MHz, CDCl_3_) δ 7.60 (dd, *J* = 8.6, 5.9 Hz, 1H), 7.32 (dd, *J* = 8.1, 2.6 Hz, 1H), 6.97 (td, *J* = 8.3, 2.7 Hz, 1H), 4.50 (s, 2H), 2.89 (ddd, *J* = 95.7, 16.2, 5.6 Hz, 2H), 1.95–1.87 (m, 2H), 1.68 (dd, *J* = 9.9, 6.5 Hz, 2H), 1.58–1.48 (m, 2H), 1.46–1.34 (m, 4H), 1.30–1.25 (m, 1H), 1.20 (s, 3H), 0.99 (dd, *J* = 12.2, 2.1 Hz, 1H), 0.87 (s, 3H), 0.86 (s, 3H), 0.79 (s, 3H). ^13^C NMR (126 MHz, CDCl_3_) δ 170.4, 162.9, 162.0 (d, *J* = 251.6 Hz), 132.6 (d, *J* = 8.5 Hz), 131.7 (d, *J* = 3.5 Hz), 124.8 (d, *J* = 9.7 Hz), 120.3 (d, *J* = 24.8 Hz), 114.9 (d, *J* = 21.1 Hz), 73.3, 59.1, 55.8, 44.6, 41.6, 39.4, 38.9, 36.4, 33.4, 33.3, 23.3, 21.5, 21.2, 20.4, 18.4, 15.2.^19^F NMR (376 MHz, CDCl_3_) δ -111.25. HRMS (ESI+) m/z Calcd for C_24_H_33_FBrSN_2_O_2_ [M + H]^+^ 511.14247; Found 511.14197.

**(1*R*,2*R*,8a*S*)-1-((5-((3-chloro-2-fluorobenzyl)thio**)**-1,3,4-oxadiazol-2-yl)methyl**)**-2,5,5,8a-tetramethyldecahydronaphthalen-2-ol (H17).** Yield 61%; White solid; m. p.79–81°C. ^1^H NMR (500 MHz, CDCl_3_) δ 7.44–7.39 (m, 1H), 7.36–7.32 (m, 1H), 7.03 (dt, *J* = 8.2, 4.2 Hz, 1H), 4.44 (s, 2H), 2.90 (ddd, *J* = 96.4, 16.3, 5.7 Hz, 2H), 1.92 (ddd, *J* = 14.8, 8.9, 4.4 Hz, 2H), 1.76–1.66 (m, 2H), 1.56–1.47 (m, 2H), 1.45–1.32 (m, 4H), 1.31–1.26 (m, 1H), 1.20 (s, 3H), 1.00 (dd, *J* = 12.2, 2.1 Hz, 1H), 0.88 (s, 3H), 0.86 (s, 3H), 0.80 (s, 3H). ^13^C NMR (126 MHz, CDCl_3_) δ 170.5, 162.6, 156.4 (d, *J* = 250.3 Hz), 130.5, 129.7, 125.3 (d, *J* = 14.4 Hz), 124.7 (d, *J* = 4.7 Hz), 121.3 (d, *J* = 17.8 Hz), 73.3, 59.1, 55.8, 44.6, 41.6, 39.4, 38.8, 33.4, 33.3, 30.0, 23.3, 21.5, 21.2, 20.4, 18.4, 15.2.^19^F NMR (376 MHz, CDCl_3_) δ -118.45. HRMS (ESI+) m/z Calcd for C_24_H_33_FClSN_2_O_2_ [M + H]^+^ 467.19298; Found 467.19138.

**(1*R*,2*R*,8a*S*)-1-((5-((4-bromobenzyl)thio**)**-1,3,4-oxadiazol-2-yl)methyl**)**-2,5,5,8a-tetramethyldecahydronaphthalen-2-ol (H18).** Yield 62%; White solid; m. p.99–101°C. ^1^H NMR (400 MHz, CDCl_3_) δ 7.46 (s, 1H), 7.44 (s, 1H), 7.31 (s, 1H), 7.29 (s, 1H), 4.36 (s, 2H), 2.89 (ddd, *J* = 77.1, 16.3, 5.7 Hz, 2H), 1.96–1.85 (m, 2H), 1.63–1.42 (m, 4H), 1.41–1.29 (m, 4H), 1.28–1.24 (m, 1H), 1.20 (s, 3H), 0.99 (dd, *J* = 12.1, 2.2 Hz, 1H), 0.88 (s, 3H), 0.86 (s, 3H), 0.80 (s, 3H). ^13^C NMR (100 MHz, CDCl_3_) δ 170.3, 162.7, 135.0, 131.8, 131.8, 130.8, 122.0, 122.0, 73.2, 59.0, 55.8, 44.5, 41.5, 39.4, 38.8, 36.1, 33.4, 33.2, 23.3, 21.4, 21.1, 20.4, 18.3, 15.1. HRMS (ESI+) m/z Calcd for C_24_H_34_BrSN_2_O_2_ [M + H]^+^ 493.15189; Found 493.15070.

**(1*R*,2*R*,8a*S*)-1-((5-((4-methoxybenzyl)thio**)**-1,3,4-oxadiazol-2-yl)methyl**)**-2,5,5,8a-tetramethyldecahydronaphthalen-2-ol (H19).** Yield 70%; White solid; m. p.86–88°C. ^1^H NMR (400 MHz, CDCl_3_) δ 7.34 (s, 1H), 7.32 (s, 1H), 6.86 (s, 1H), 6.84 (s, 1H), 4.39 (s, 2H), 3.79 (s, 3H), 2.90 (ddd, *J* = 78.2, 16.3, 5.6 Hz, 2H), 1.92 (ddd, *J* = 16.1, 8.8, 4.4 Hz, 2H), 1.77–1.65 (m, 2H), 1.60–1.49 (m, 2H), 1.46–1.33 (m, 4H), 1.29–1.23 (m, 1H), 1.20 (s, 3H), 1.00 (dd, *J* = 12.1, 2.2 Hz, 1H), 0.88 (s, 3H), 0.87 (s, 3H), 0.80 (s, 3H). ^13^C NMR (100 MHz, CDCl_3_) δ 170.1, 163.2, 159.3, 130.3, 127.5, 114.1, 73.2, 59.0, 55.7, 55.2, 44.4, 41.5, 39.3, 38.8, 36.4, 33.3, 33.2, 23.2, 21.4, 21.1, 20.3, 18.3, 15.1. HRMS (ESI+) m/z Calcd for C_25_H_37_SN_2_O_3_ [M + H]^+^ 445.25194; Found (H20). Yield 75%; White solid; m. p.98–100°C. ^1^H NMR (400 MHz, CDCl_3_) δ 7.52 (s, 1H), 4.55 (s, 2H), 2.92 (ddd, *J* = 75.5, 16.2, 5.7 Hz, 2H), 1.96–1.89 (m, 2H), 1.75–1.69 (m, 2H), 1.55–1.50 (m, 2H), 1.45–1.34 (m, 4H), 1.28–1.25 (m, 1H), 1.22 (s, 3H), 1.01 (dd, *J* = 12.2, 2.2 Hz, 1H), 0.88 (s, 3H), 0.87 (s, 3H), 0.80 (s, 3H). ^13^C NMR (100 MHz, CDCl_3_) δ 170.8, 161.9, 152.3, 140.9, 135.9, 73.2, 59.0, 55.7, 44.5, 41.5, 39.4, 38.7, 33.3, 33.2, 28.5, 23.3, 21.4, 21.1, 20.3, 18.3, 15.1. HRMS (ESI+) m/z Calcd for C_21_H_30_ClS_2_N_3_O_2_Na [M + Na]^+^ 478.13602; Found 478.13550.

**(1*R*,2*R*,8a*S*)-1-((5-((4-bromo-2-fluorobenzyl)thio**)**-1,3,4-oxadiazol-2-yl)methyl**)**-2,5,5,8a-tetramethyldecahydronaphthalen-2-ol (H21).** Yield 68%; White solid; m. p.120–122°C. ^1^H NMR (400 MHz, CDCl_3_) δ 7.40 (t, *J* = 8.3 Hz, 1H), 7.23 (dd, *J* = 7.1, 2.0 Hz, 2H), 4.38 (s, 2H), 2.89 (ddd, *J* = 77.6, 16.3, 5.6 Hz, 2H), 1.92 (ddd, *J* = 13.8, 8.7, 4.4 Hz, 2H), 1.63–1.42 (m, 4H), 1.41–1.30 (m, 4H), 1.28–1.24 (m, 1H), 1.20 (s, 3H), 1.00 (dd, *J* = 12.1, 2.2 Hz, 1H), 0.88 (s, 3H), 0.86 (s, 3H), 0.80 (s, 3H). ^13^C NMR (100 MHz, CDCl_3_) δ 170.4, 162.6, 160.6 (d, *J* = 253.0 Hz), 132.4 (d, *J* = 4.0 Hz), 127.6 (d, *J* = 3.8 Hz), 122.7 (d, *J* = 14.6 Hz), 122.3 (d, *J* = 9.5 Hz), 119.2 (d, *J* = 24.4 Hz), 73.2, 59.0, 55.7, 44.5, 41.5, 39.3, 38.7, 33.3, 33.2, 29.5, 23.3, 21.4, 21.1, 20.3, 18.3, 15.1.^19^F NMR (376 MHz, CDCl3) δ -113.84. HRMS (ESI+) m/z Calcd for C_24_H_33_BrFSN_2_O_2_ [M + H]^+^ 511.14247; Found 511.14252.

**(1*R*,2*R*,8a*S*)-2,5,5,8a-tetramethyl-1-((5-((4-(trifluoromethoxy)benzyl**)**thio)-1,3,4-oxadiazol-2-yl)methyl**)**decahydronaphthalen-2-ol (H22).** Yield 70%; White solid; m. p.83–85°C. ^1^H NMR (500 MHz, CDCl_3_) δ 7.45 (s, 1H), 7.43 (s, 1H), 7.16 (s, 1H), 7.14 (s, 1H), 4.39 (s, 2H), 2.88 (ddd, *J* = 21.7, 16.4, 5.8 Hz, 2H), 1.90 (ddd, *J* = 15.5, 7.8, 4.4 Hz, 2H), 1.60–1.39 (m, 4H), 1.38–1.22 (m, 4H), 1.19 (s, 3H), 1.14–1.07 (m, 1H), 0.98 (dd, *J* = 12.1, 2.2 Hz, 1H), 0.86 (s, 3H), 0.85 (s, 3H), 0.78 (s, 3H). ^13^C NMR (126 MHz, CDCl_3_) δ 170.4, 162.8, 148.9, 134.7, 130.7, 121.2, 120.4 (d, *J* = 257.8 Hz), 73.3, 59.1, 55.8, 44.6, 41.6, 39.4, 38.8, 35.9, 33.4, 33.3, 23.4, 21.5, 21.2, 20.4, 18.4, 15.2.^19^F NMR (376 MHz, CDCl_3_) δ -57.72. HRMS (ESI+) m/z Calcd for C_25_H_33_F_3_SN_2_O_3_ [M + H]^+^ 521.20562; Found 521.20575.

**(1*R*,2*R*,8a*S*)-2,5,5,8a-tetramethyl-1-((5-((4-nitrobenzyl)thio**)**-1,3,4-oxadiazol-2-yl)methyl**)**decahydronaphthalen-2-ol (H23).** Yield 53%; White solid; m. p.105–107°C. ^1^H NMR (500 MHz, CDCl_3_) δ 8.17 (s, 1H), 8.15 (s, 1H), 7.61 (s, 1H), 7.59 (s, 1H), 4.45 (s, 2H), 2.87 (ddd, *J* = 21.6, 16.3, 5.7 Hz, 2H), 1.94–1.83 (m, 2H), 1.60–1.39 (m, 4H), 1.38–1.29 (m, 4H), 1.18 (s, 3H), 1.13–1.06 (m, 1H), 0.96 (dd, *J* = 12.2, 2.1 Hz, 1H), 0.86 (s, 3H), 0.84 (s, 3H), 0.78 (s, 3H). ^13^C NMR (126 MHz, CDCl_3_) δ 170.7, 162.2, 147.5, 143.7, 130.1, 124.0, 73.3, 59.1, 55.8, 44.6, 41.6, 39.4, 38.8, 35.7, 33.4, 33.3, 23.4, 21.5, 21.2, 20.4, 18.4, 15.2. HRMS (ESI+) m/z Calcd for C_24_H_34_SN_3_O_4_ [M + H]^+^ 460.22645; Found 460.22681.

### Biological Activity Test Method

The *in vitro* antibacterial activities of target compounds **H1-H23** against *Xoo* and *Xac* was evaluated by the turbidity method (Zhang et al., 2021). According to Schaad’s method (Zhang et al., 2021), the curative and protective activities of compound **H8** against rice bacterial blight were determined *in vivo*. Based on the previous work (Wang et al., 2019; Luo et al., 2020), TMV was extracted and purified, and the interaction mode of active molecules with TMV-CP was explored by molecular docking. Detailed methods for bacterial bioactivity testing, as well as specific steps for TMV extraction and purification can be found in the Supplementary Datasheet S1.

## Conclusion

In conclusion, a series of 1,3,4-oxadiazole contained sesquiterpene derivatives were synthesized, and the biological activity of title compounds was evaluated. The results exhibited that the synthetic compounds had good antibacterial activity against *Xoo* and *Xac*. The EC_50_ values of compounds **H4, H8, H11, H12, H14, H16,** and **H19** for *Xac* inhibitory activity were 33.3, 42.7, 56.1, 74.5, 37.8, 43.8, and 38.4 *μg*/ml, respectively. Compounds **H4, H8, H15, H19, H22**, and **H23** had inhibitory effects on *Xoo*, with EC_50_ values of 51.0, 43.3, 43.4, 50.5, 74.6, and 51.4 *μg*/ml, respectively. In particular, the curative and protective activities of compound **H8** were 51.9 and 49.3%, respectively, showing good antibacterial activity against *Xoo in vitro*. In addition, the EC_50_ values of the inactivation activities of the compounds **H4, H5, H9, H10,** and **H16** against TMV were 69.6, 58.9, 69.4, 43.9, and 60.5 *μg*/ml, respectively. It is worth noting that the molecular docking results indicated that compound **H10** binds to the active site of TMV-CP through amino acid residues ASN73, VAL260, TYR139, ELU131, and THR136. And it existed a strong affinity for TMV-CP, with a binding energy of -8.88 kcal/mol. Thus, the process of self-assembly and replication of TMV particles is inhibited and the anti-TMV effect is played.

## Data Availability

The original contributions presented in the study are included in the article/Supplementary Material, further inquiries can be directed to the corresponding author.
