# Supplementary material for: 1,3,4-Oxadiazole Contained Sesquiterpene Derivatives: Synthesis and Microbiocidal Activity for Plant Disease
Source: Front Chem. 2022 Feb 22;10:854274. doi: 10.3389/fchem.2022.854274 (PMC8902154; doi:10.3389/fchem.2022.854274)
Supplement: Supplementary file 1 [file DataSheet1.PDF]

Supplementary Material

1,3,4-Oxadiazole Contained Sesquiterpene Derivatives: Synthesis and Microbiocidal Activity for Plant Disease

Ali Dai, Zhiguo Zheng, Lijiao Yu, Yuanqin Huang, Jian Wu*

State Key Laboratory Breeding Base of Green Pesticide and Agricultural Bioengineering, Key Laboratory of Green Pesticide and Agricultural Bioengineering, Ministry of Education, Guizhou University, Huaxi District, Guiyang 550025, China.

*Authors to whom correspondence should be addressed; Tel.: +86-851-88292090. Fax: +86-851-88292090. E-mail: jwu6@gzu.edu.cn; wujian2691@126.com

Table of Contents

1. Biological activity test method 3

1.1 *In Vitro* Antibacterial Activity Test 3

1.2 *In Vivo* Antibacterial Activity Test 3

1.3 Purification of the viruses 4

2. ^{1}H NMR, ^{13}C NMR, and ^{19}F NMR Spectra of the title compounds H1 - H23 5

3. HRMS Spectra of the title compounds H1 - H23 35

4. Reference 47

1. Biological activity test method

1.1 *In Vitro* Antibacterial Activity Test

In the work described in this paper, all target compounds were evaluated for their antibacterial activity against *Xoo* and *Xac* by *in vitro* turbidity test.[1] Dimethyl sulfoxide (DMSO) in sterile distilled water was used as a blank control, and bismerthiazol and thiodiazole copper were used as positive controls. To a 15 mL tube, 4 mL of nutrient broth (NB) media, 1 mL of the test compounds or the commercial bactericides solution (final concentration: 100 and 50 μg/mL), and 40 µL of *Xoo* or *Xac* bacterium solution were added. And the preliminary bioassay is based on 100, 50, 2 5, 12.5, 6.25 μg/mL (according to the biological activity of different compounds, select the concentration according to the following downward trend to ensure that the EC_{50} value is within the test concentration range). Then, incubate the test tube in a constant temperature shaker flask at 180 rpm and 28±1°C for 24–48h. By measuring the optical density at 595 nm (OD_{595}) (turbidity correction value = $OD_{\text{Value of medium containing bacteria}}$ – $OD_{\text{Medium value without bacteria}}$), monitor the growth of the culture on the microplate reader, and the inhibition rate *I* was calculated by $I = (C - T) / C \times 100\%$. *C* represents the corrected absorbance value (OD_{595}) of the untreated NB medium, and *T* represents the corrected absorbance value of the treated NB medium. On the basis of preliminary biological activity, the EC_{50} values were also determined and calculated via software SPSS 17.0. Each experiment was repeated three times.

1.2 *In Vivo* Antibacterial Activity Test

The curative and protection activities in potted plants of compound **H8** against rice bacterial leaf blight were determined by Schaad's method.[2] Bismerthiazol (20% wettable powder) and thiodiazole copper (20% suspending agent, the bactericides registered for rice bacterial leaf blight and purchased from the market, served as the positive controls. Under greenhouse control conditions, the curative activity of compound **H8** against rice bacterial leaf blight was determined. Inoculate *Xoo* on rice

leaves, and then use sterile scissors for logarithmic growth culture. On the first day after inoculation, 200 μg/mL of compound **H8** solution was sprayed evenly on the rice leaves until dripping, and distilled water was sprayed evenly on the negative control plants. Then, place all the inoculated rice plants in a plant growth room (28°C and 90% RH). 14 days after spraying, the disease index of the inoculated rice leaves was determined. To test the protective activity of compound **H8** in the same way, 200 μg/mL of compound **H8** solution was sprayed evenly on the rice leaves until it dripped, and distilled water was sprayed evenly on the negative control plants. After spraying for one day, use sterile scissors to inoculate logarithmic growth of *Xoo* on rice leaves. Place all the inoculated rice plants in a growth room (28°C and 90% RH). 14 days after the inoculation, the disease index of the inoculated rice leaves was measured.

The control efficiencies *I* (%) for the curative and protection activities are calculated by the following equation. In the equation, *C* is the disease index of the negative control and *T* is the disease index of the treatment group.

$$I\ (\%) = (C - T) / C \times 100$$

Statistical analysis was conducted by ANOVA with software SPSS 17.0. Different uppercase letters following the control efficiency values indicate that there is significant difference ($P < 0.05$) among different treatment groups.

1.3 Purification of the viruses

Extraction of TMV. According to the reference method,[3-5] tobacco (Nicotiana tabacum L.) the tobacco that has been infected with TMV virus is selected, and the seriously infected leaves are cut and placed in a mortar, added with liquid nitrogen to grind, and then poured into a volume of phosphate (pH 7.20, 0.01 mol/L) buffer that adds twice the weight of the leaves. After sufficient grinding, filter with gauze and centrifuge the filtrate under specific conditions. The specific steps of the purification steps are as follows:

1). The severely infected leaves are cut and placed in a mortar and treated with liquid nitrogen. Then double the volume of phosphate buffer solution (pH 7.20, 0.01 mol/L)

of the blade weight, grind, then add 10% chloroform / n-butanol (1: 1) solution, grind it thoroughly and filter with four layers of gauze.

2). Centrifuge for 20 minutes (Condition: 8000g, 4 °C, 260 nm), and add PEG (6%) to the filtered supernatant.

iii). Stir the supernatant with a mixture of PEG (6%) and NaCl (0.1 mol) for 4h, and centrifuge for 20 min. The precipitate was completely suspended in PBS (0.02 mol/L). Then repeat the centrifugation twice.

3). Combine the above supernatant, centrifuge again (78000 g) for 2 hours, and suspend the pellet in PBS (0.02 mol/L). After centrifuging the suspension at low speed, the supernatant was added to the centrifuge tube together with 25% glycerol for 1.5h (78000 g).

Finally, the purified virus was precipitated and suspended in glycerin.

$$\text{virus concn} = (\text{A260} \times \text{dilution ratio}) / \text{E1cm}^{0.1\%,\ 260\ \text{nm}}.$$

2. ^{1}H NMR, ^{13}C NMR, and ^{19}F NMR Spectra of the title compounds H1 - H23

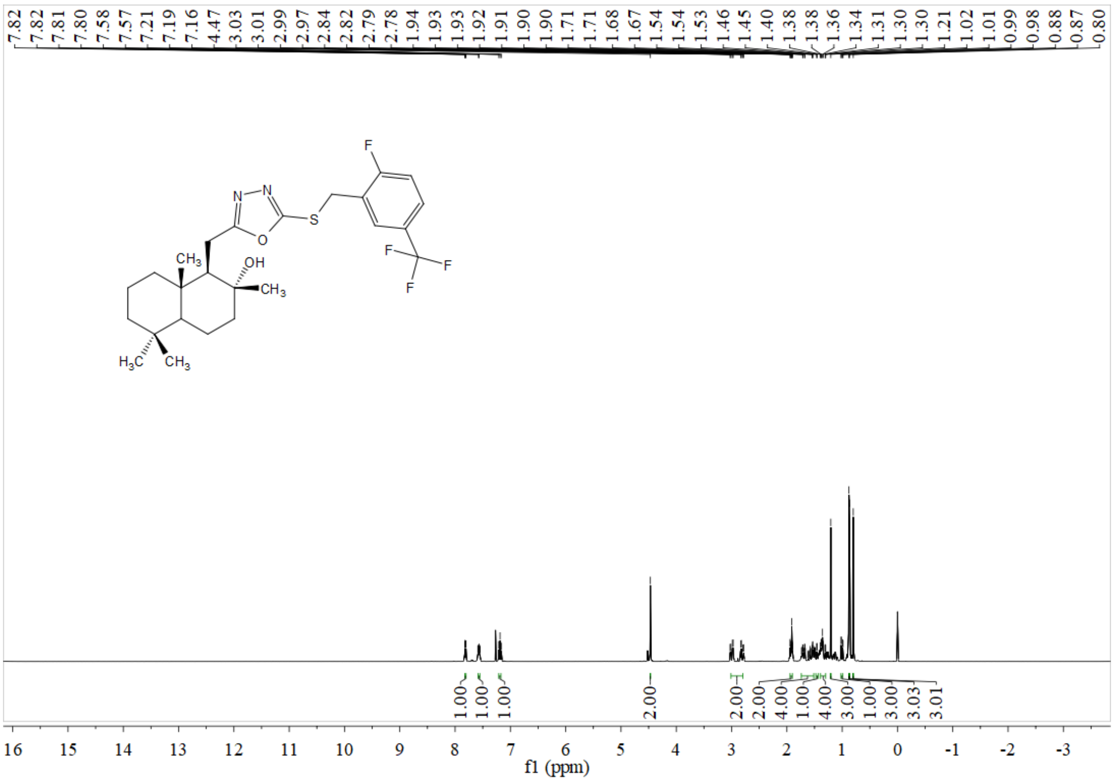

Figure S1 ^{1}H NMR Spectrum of **H1**

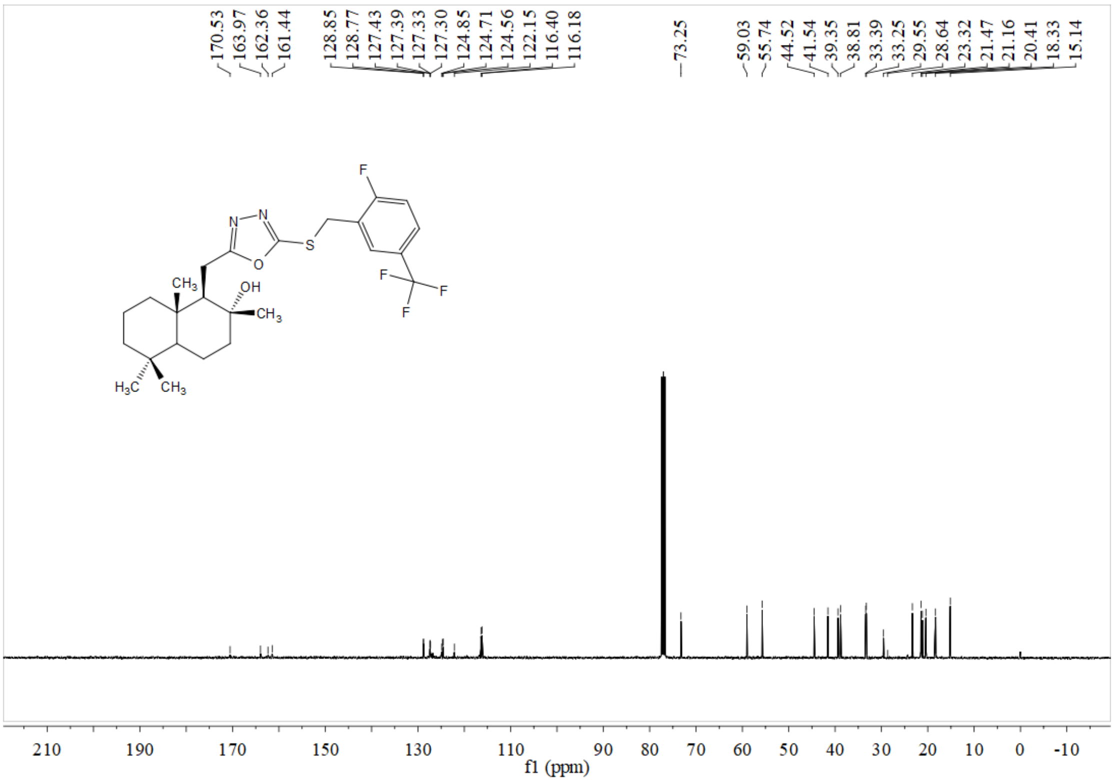

Figure S2 ^{13}C NMR Spectrum of **H1**

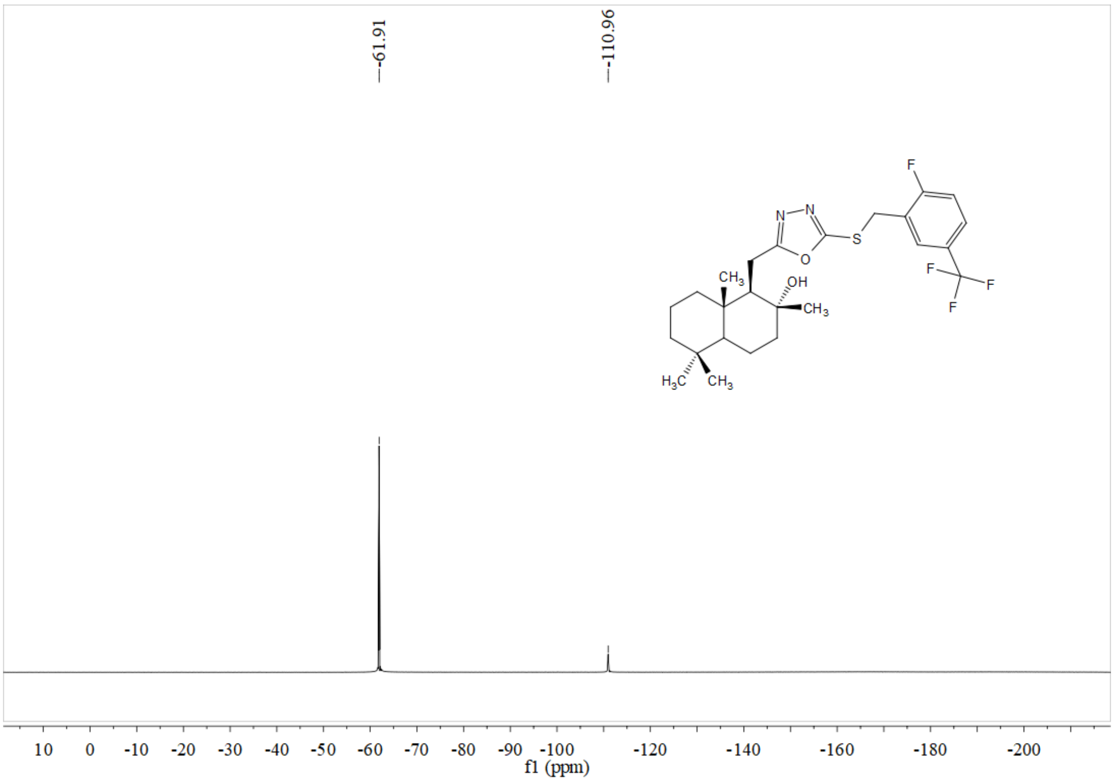

Figure S3 ^{19}F NMR Spectrum of **H1**

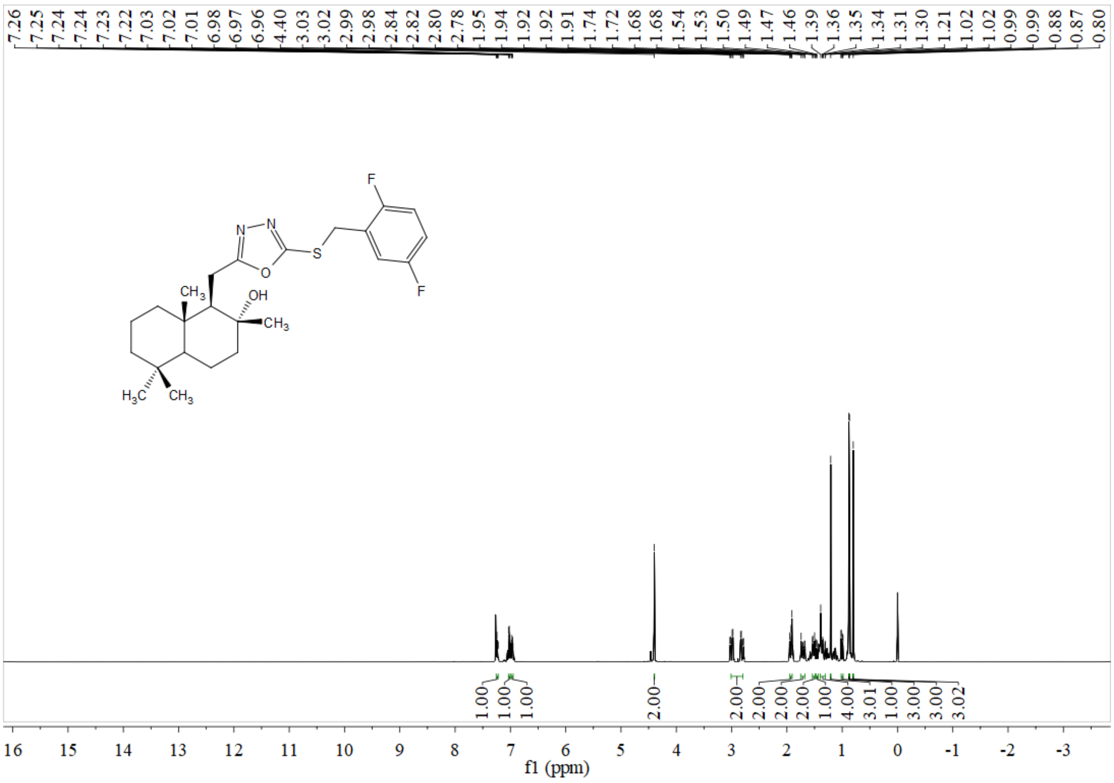

Figure S4 ^{1}H NMR Spectrum of **H2**

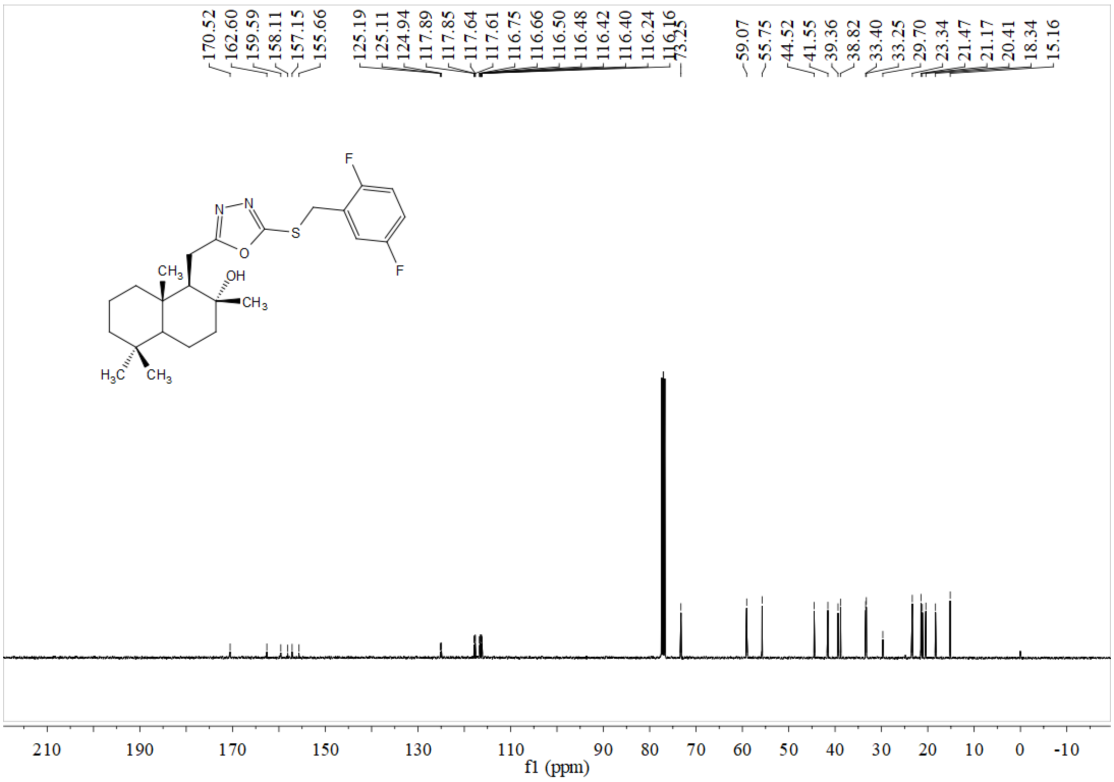

Figure S5 ^{13}C NMR Spectrum of **H2**

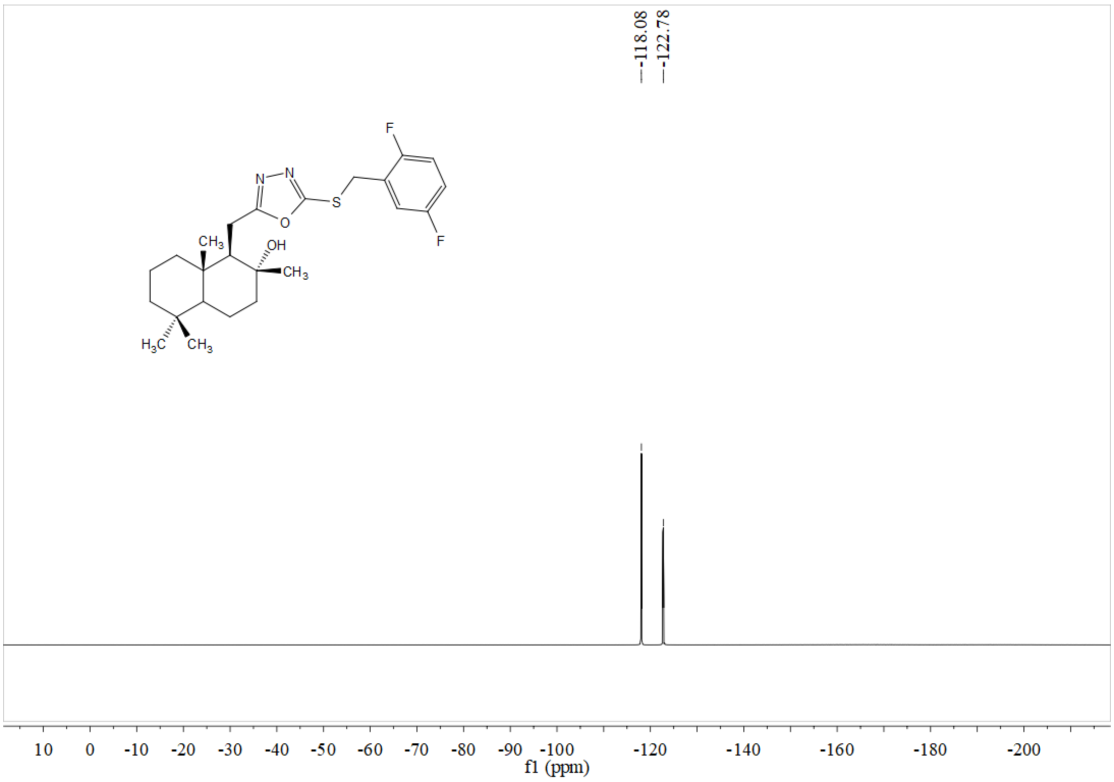

Figure S6 ^{19}F NMR Spectrum of **H2**

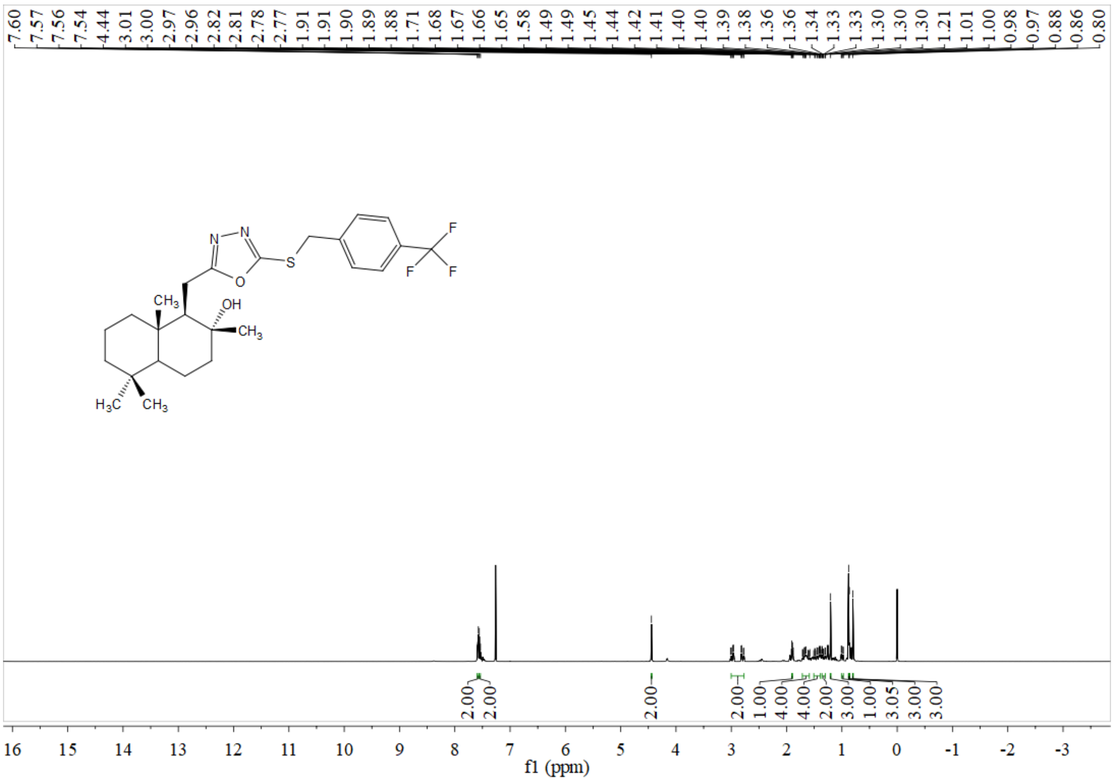

Figure S7 ^{1}H NMR Spectrum of **H3**

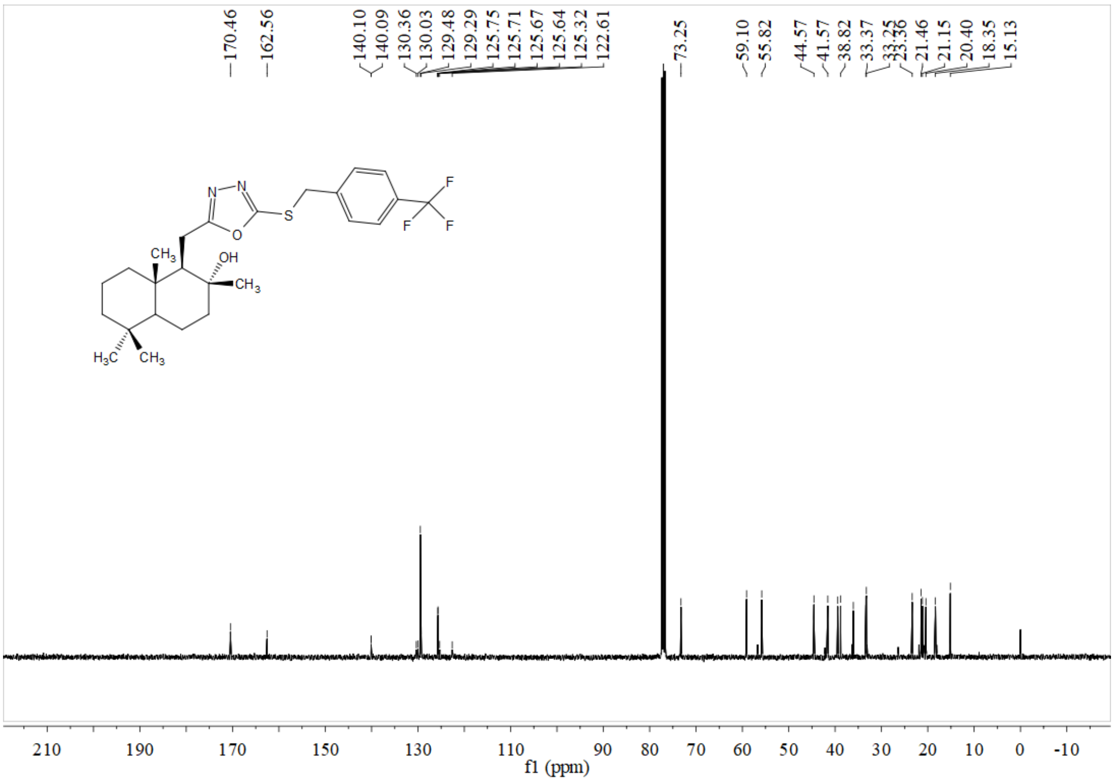

Figure S8 ^{13}C NMR Spectrum of **H3**

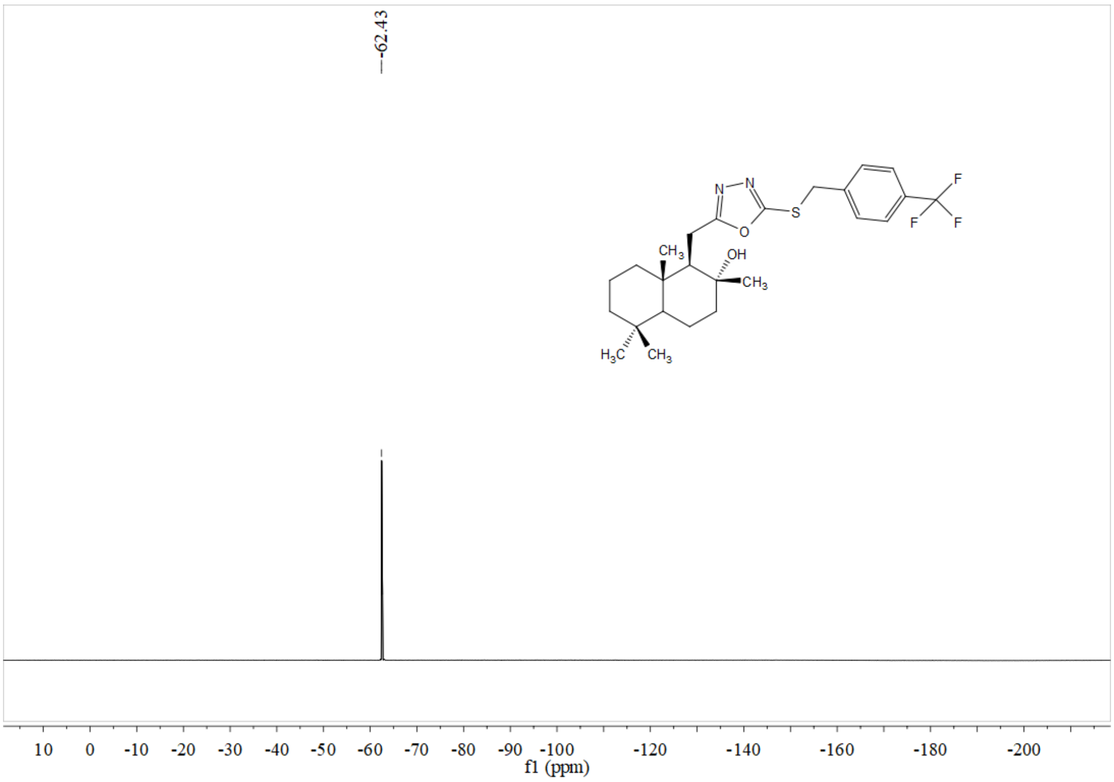

Figure S9 ^{19}F NMR Spectrum of **H3**

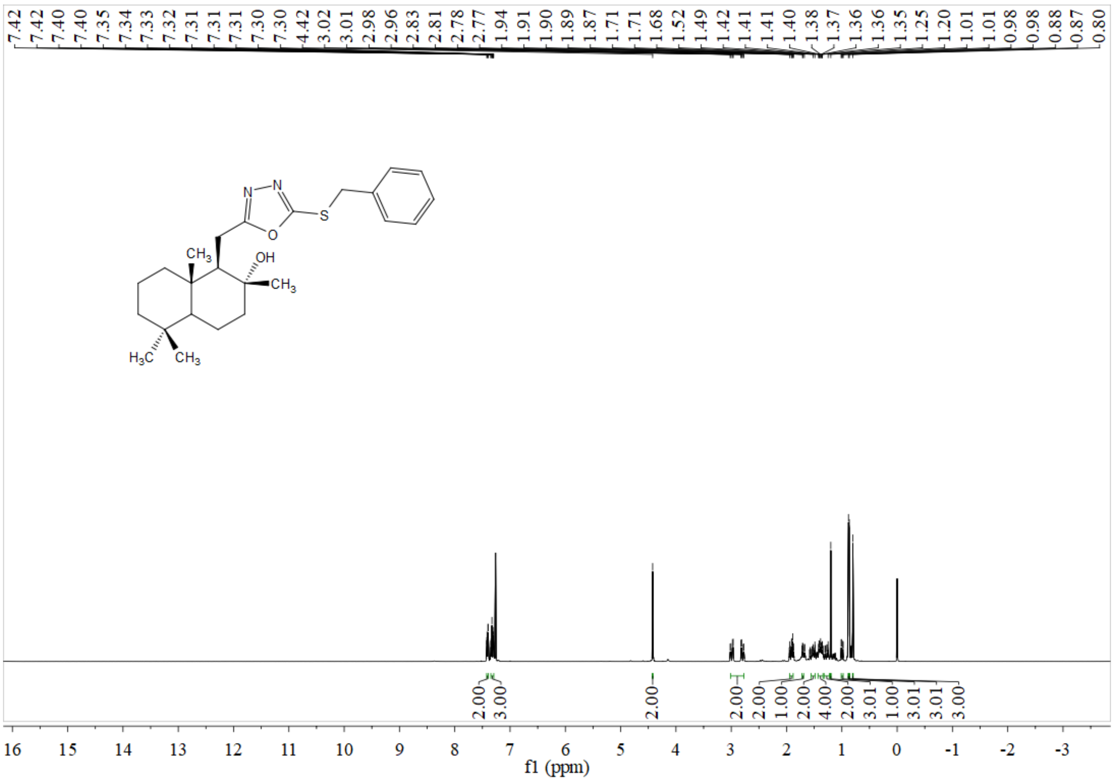

Figure S10 ^{1}H NMR Spectrum of **H4**

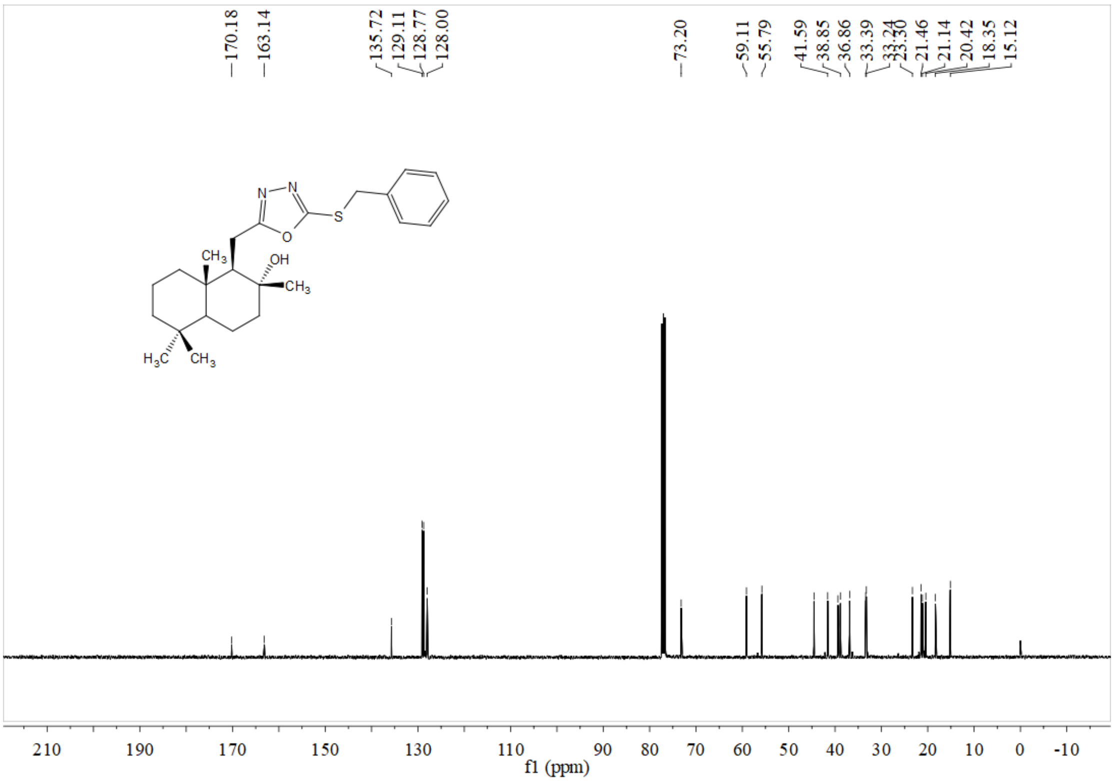

Figure S11 ^{13}C NMR Spectrum of **H4**

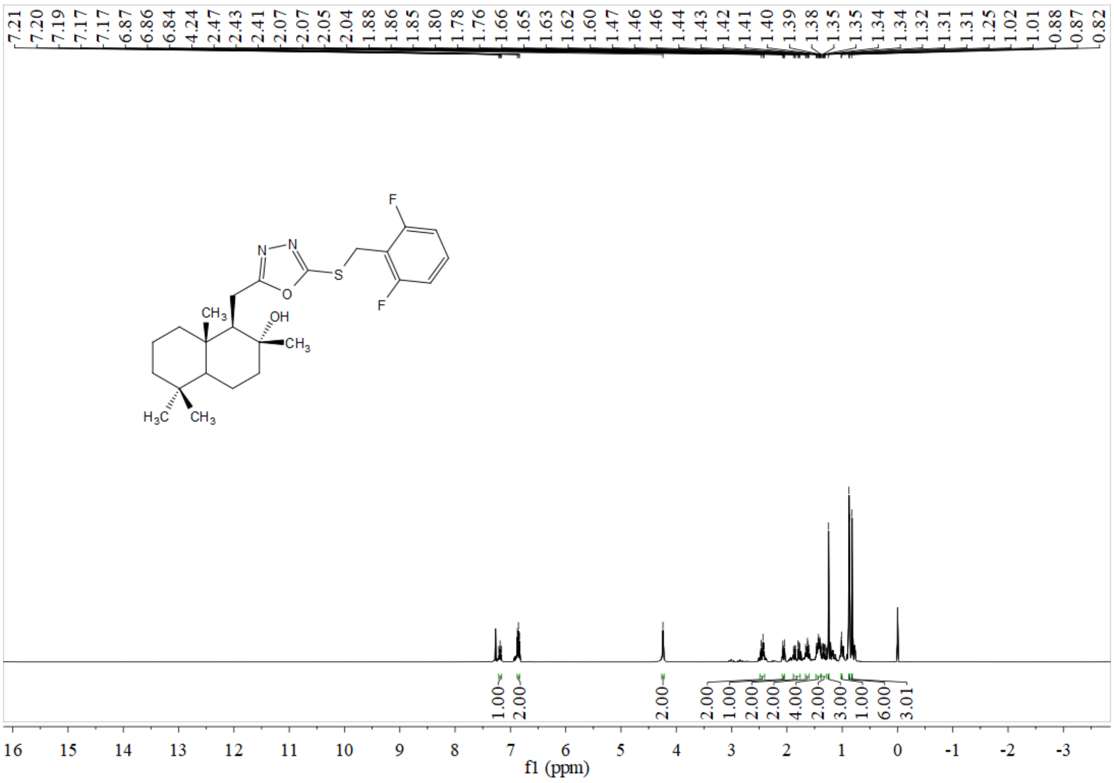

Figure S12 ^{1}H NMR Spectrum of **H5**

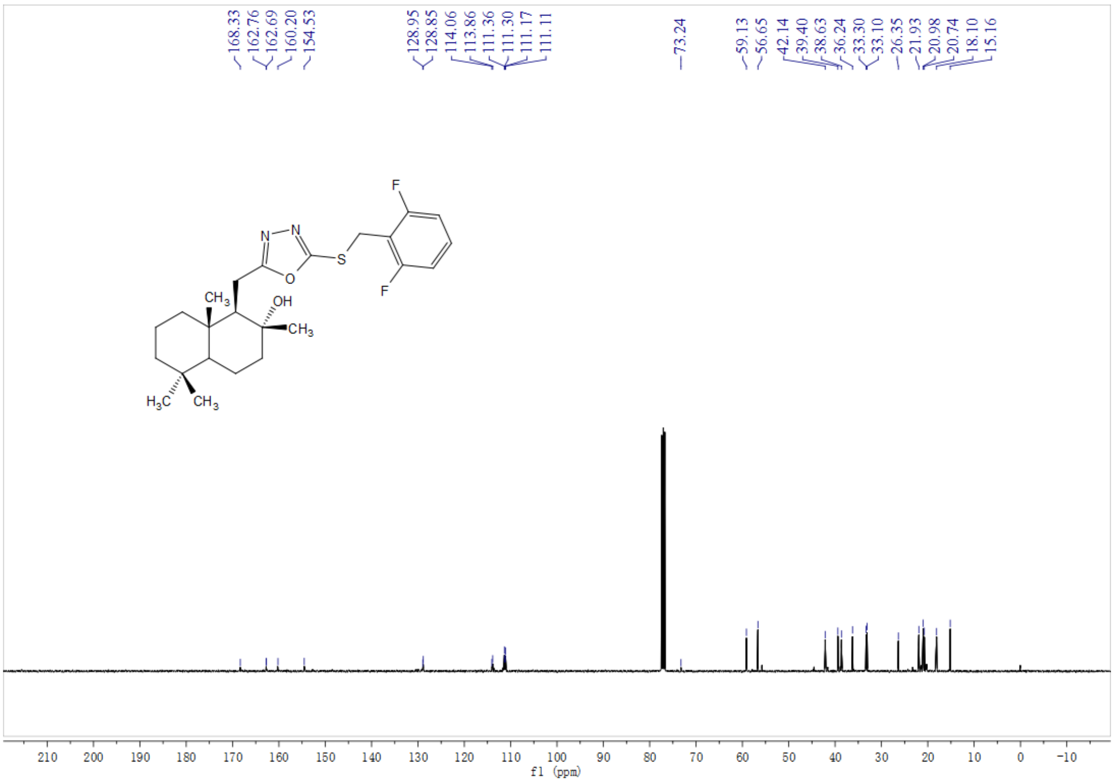

Figure S13 ^{13}C NMR Spectrum of **H5**

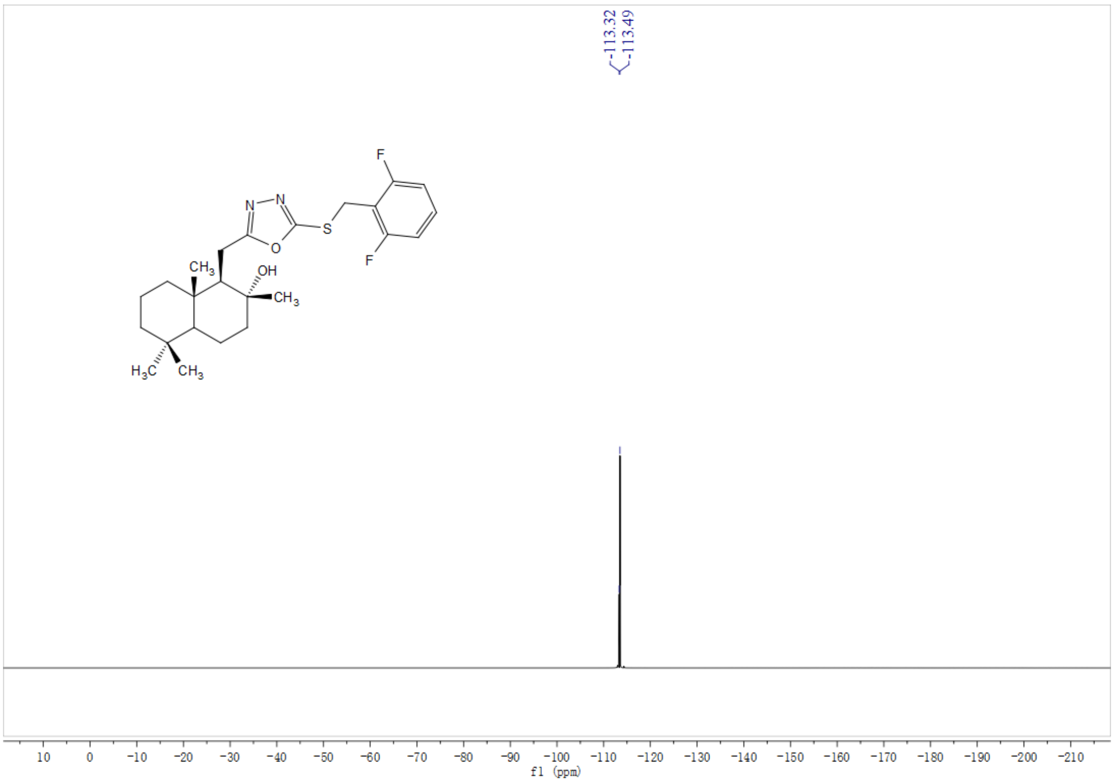

Figure S14 ^{19}F NMR Spectrum of **H5**

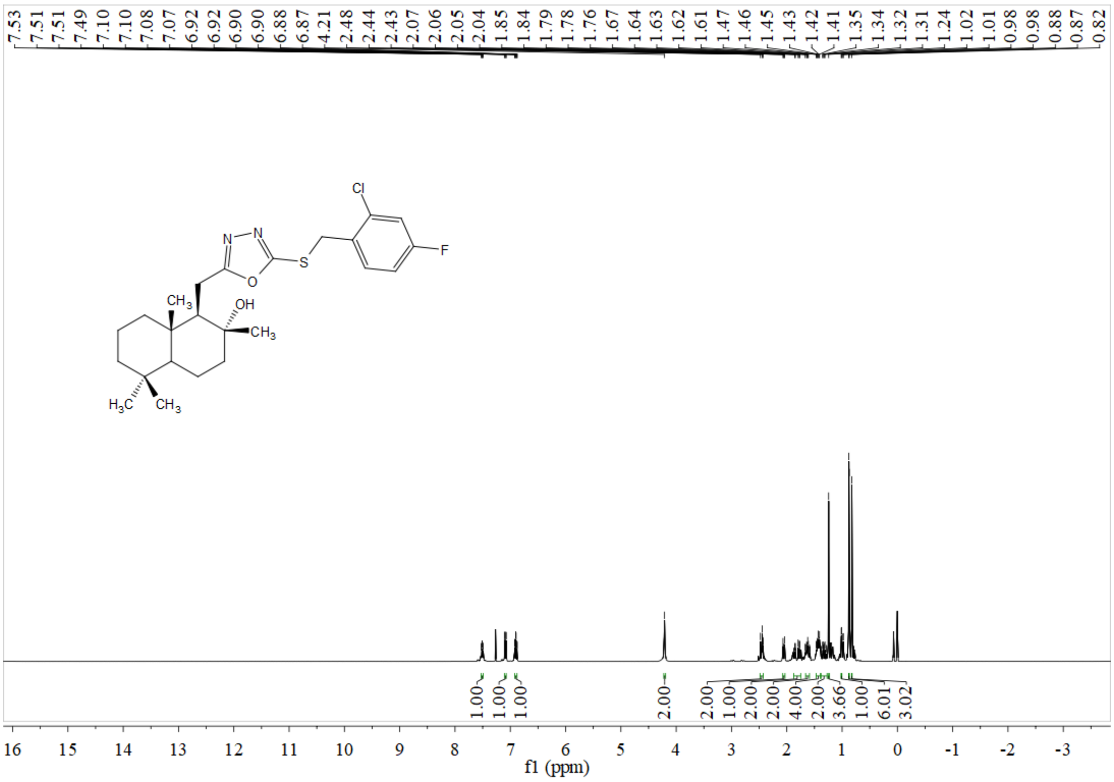

Figure S15 ^{1}H NMR Spectrum of **H6**

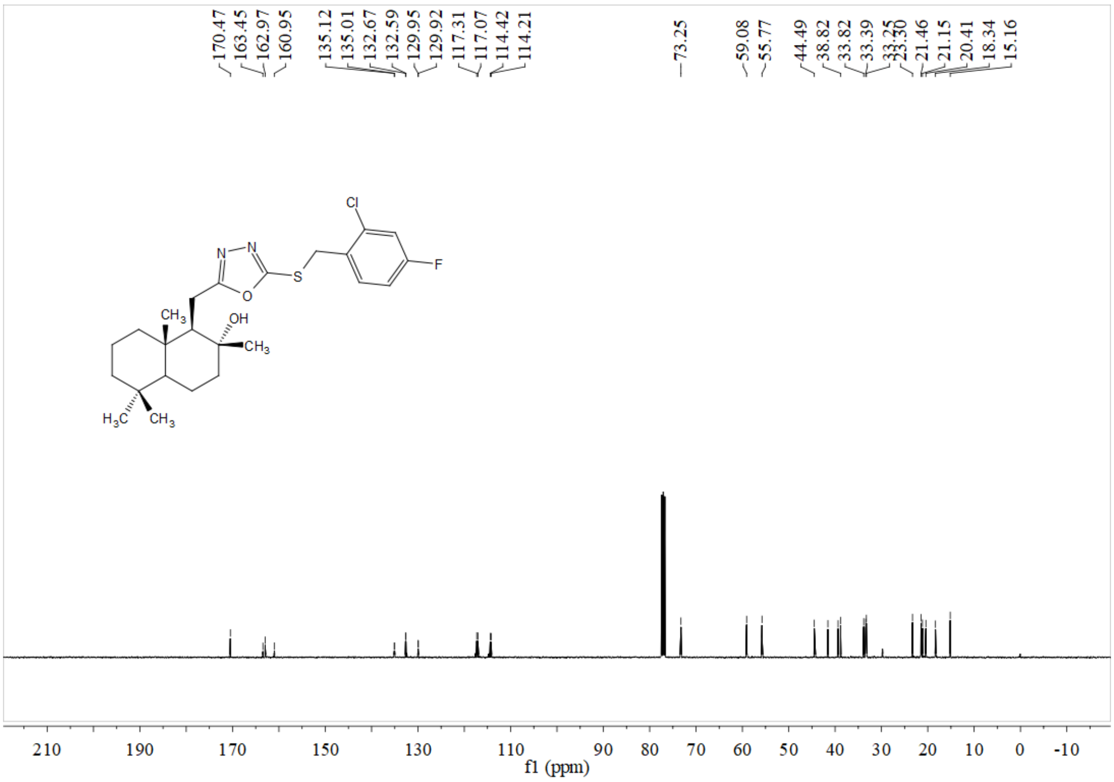

Figure S16 ^{13}C NMR Spectrum of **H6**

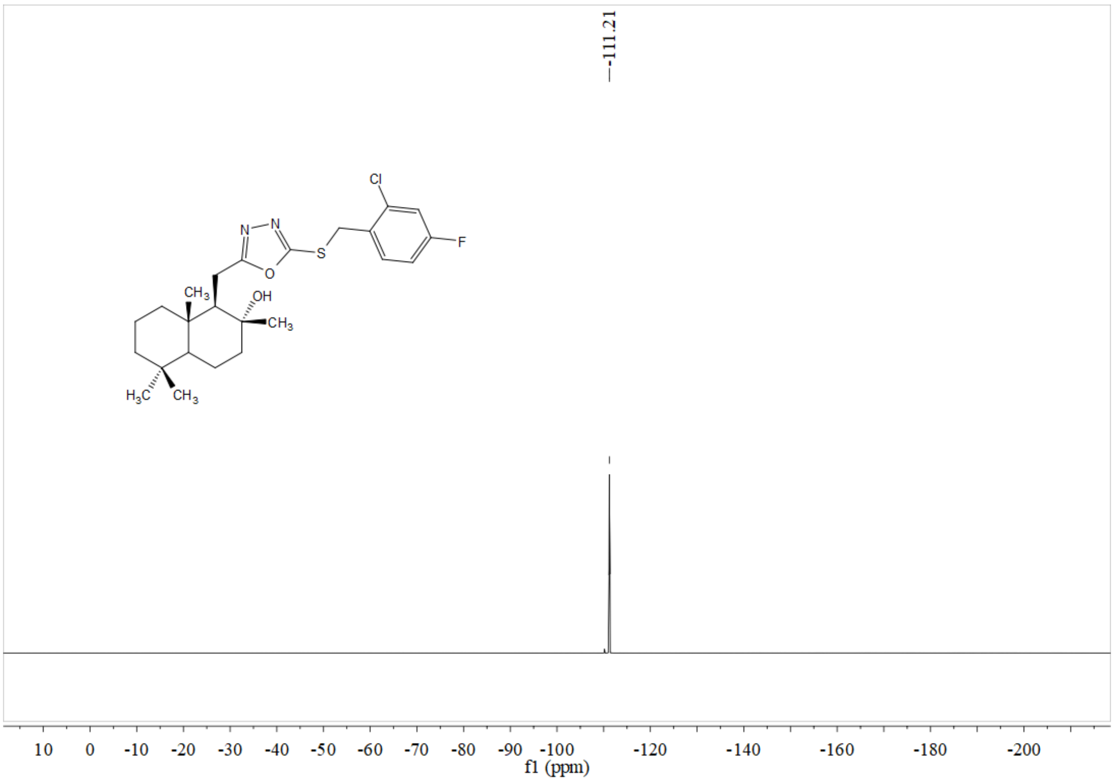

Figure S17 ^{19}F NMR Spectrum of **H6**

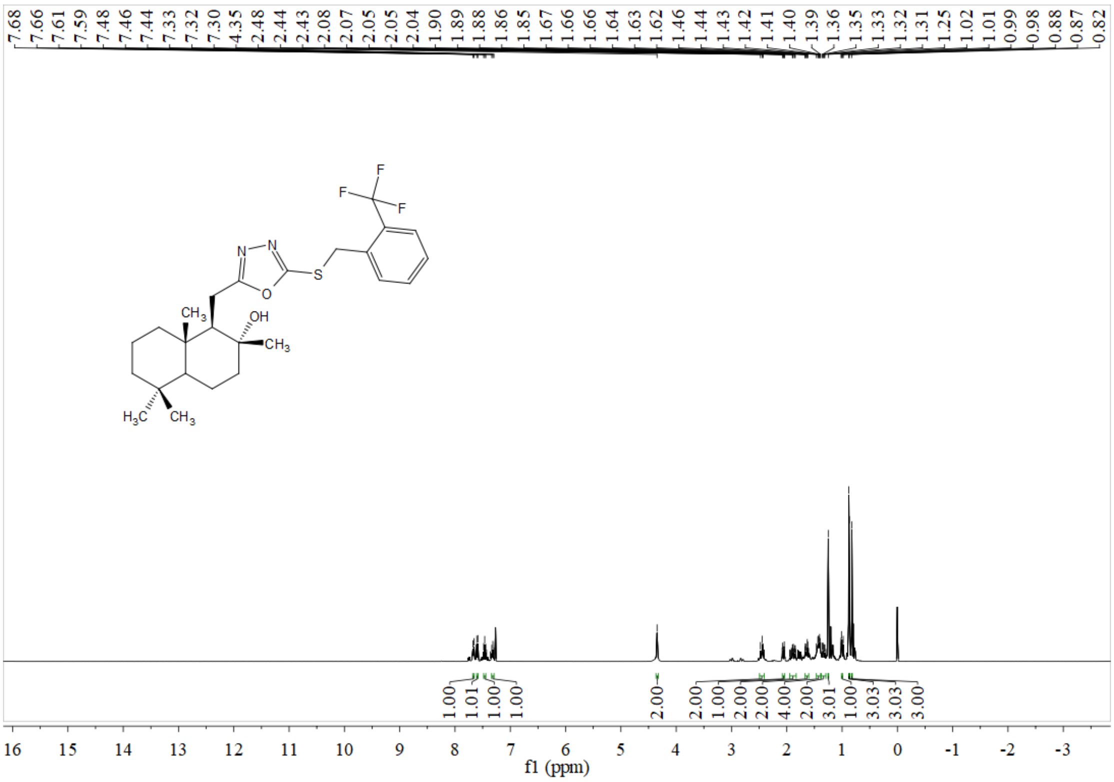

Figure S18 ^{1}H NMR Spectrum of **H7**

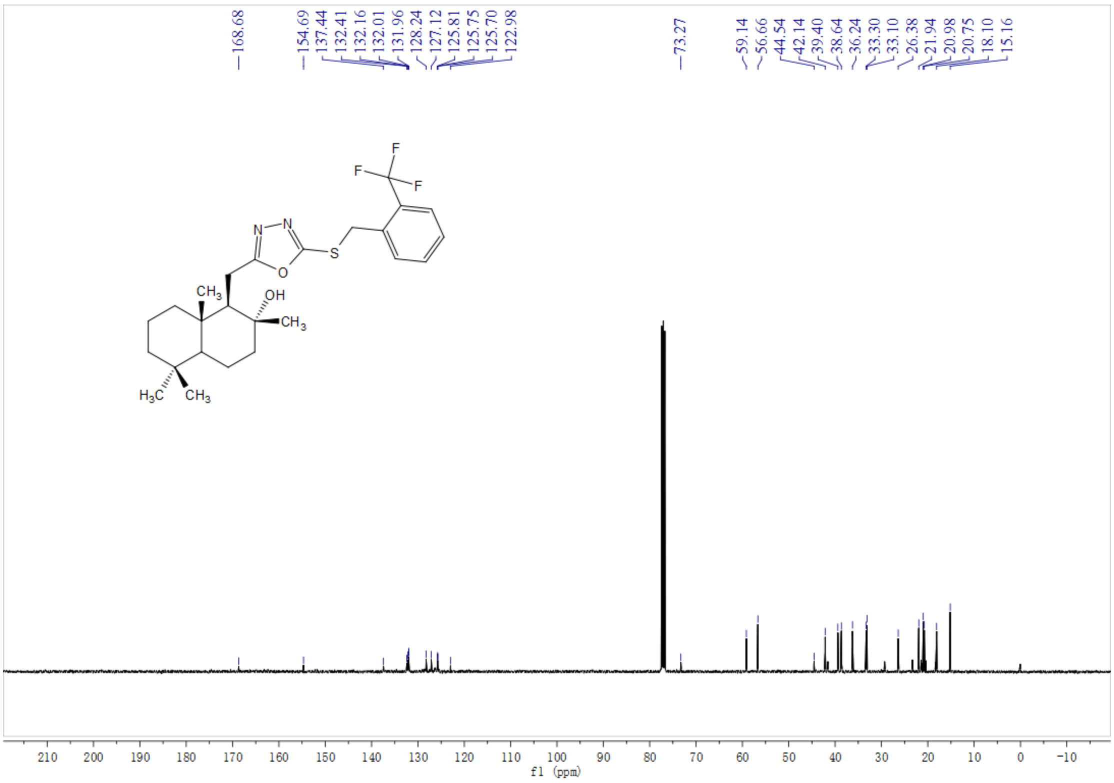

Figure S19 ^{13}C NMR Spectrum of **H7**

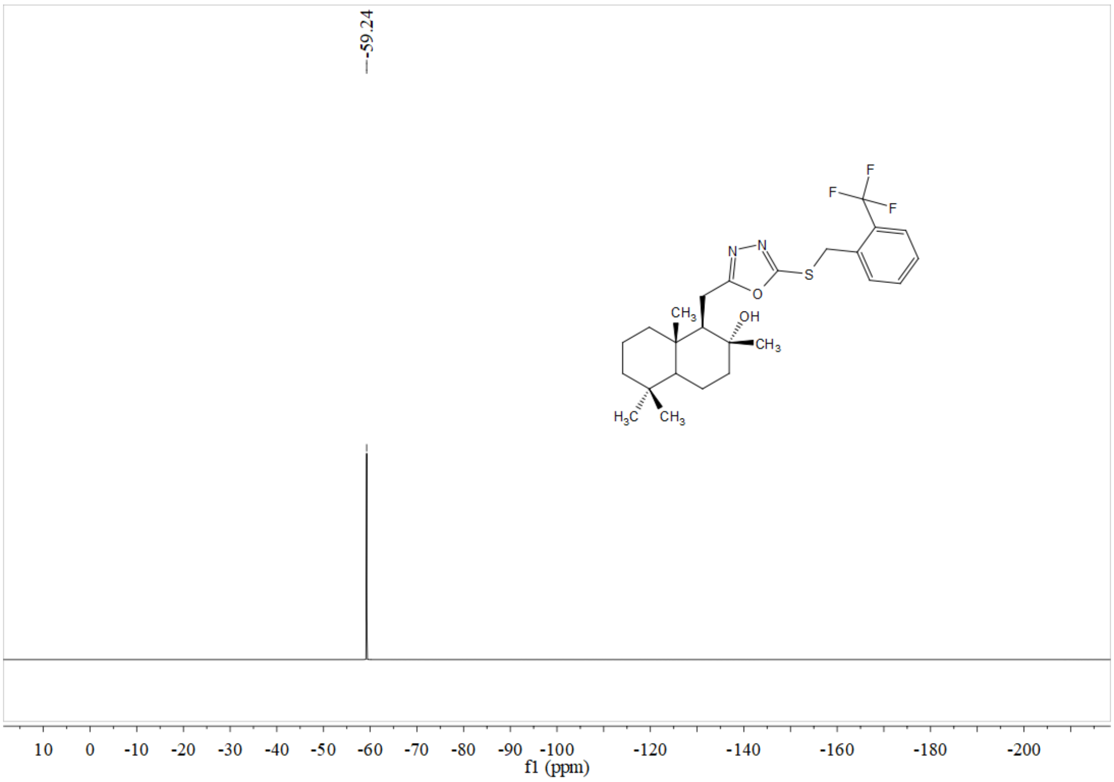

Figure S20 ^{19}F NMR Spectrum of **H7**

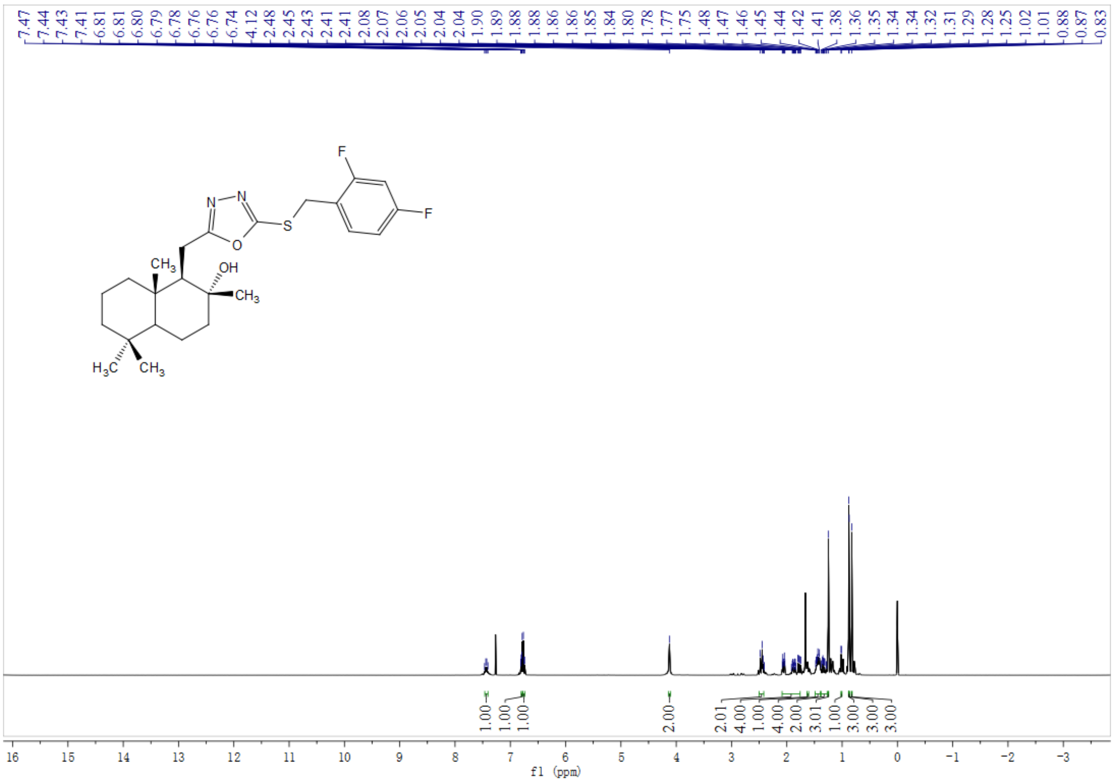

Figure S21^{1}H NMR Spectrum of **H8**

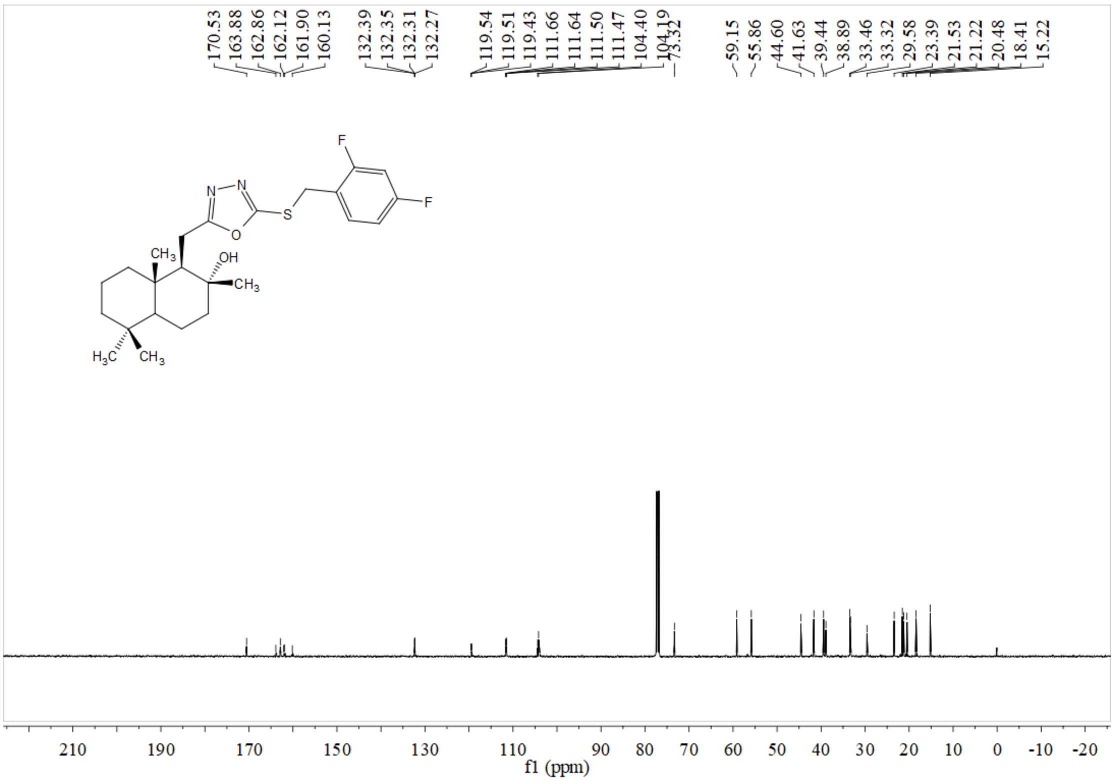

Figure S22 ^{13}C NMR Spectrum of **H8**

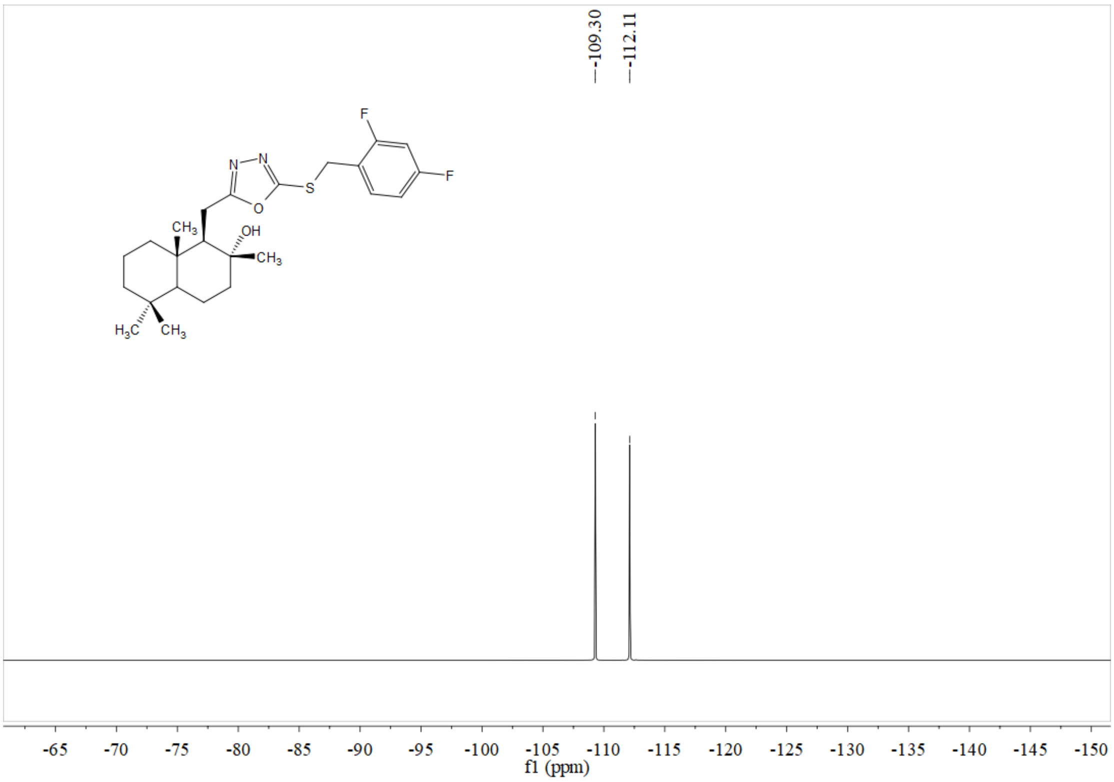

Figure S23 ^{19}F NMR Spectrum of **H8**

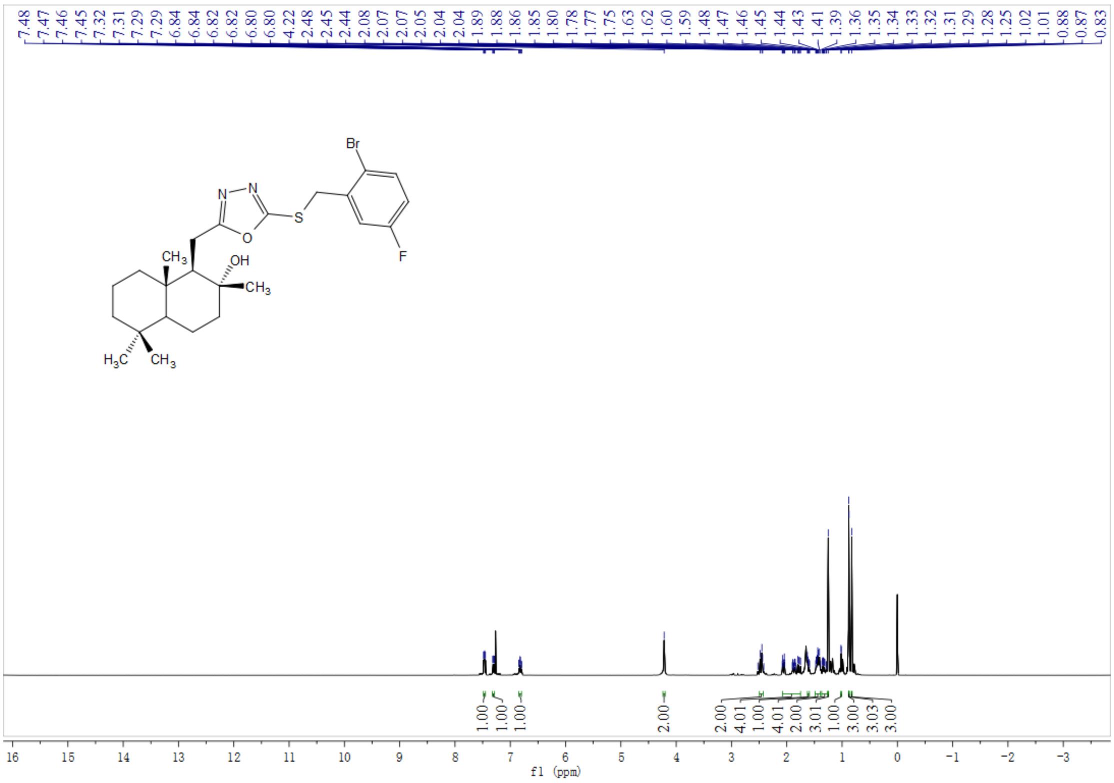

Figure S24 ^{1}H NMR Spectrum of **H9**

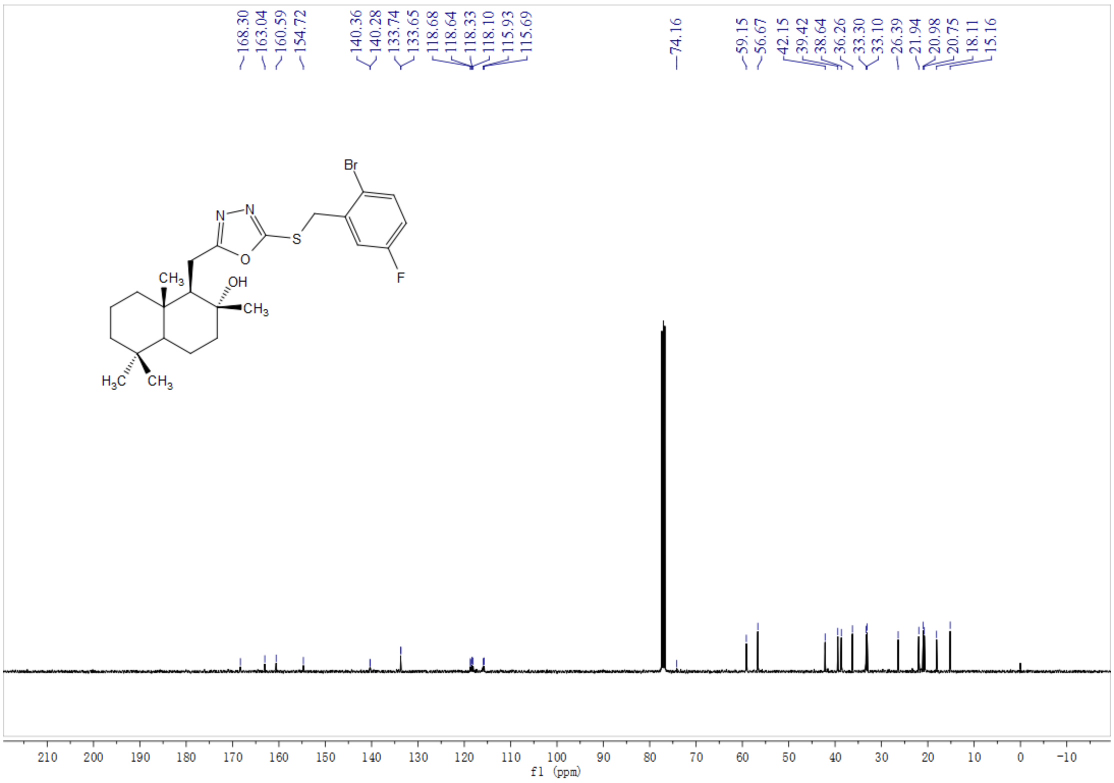

Figure S25 ^{13}C NMR Spectrum of **H9**

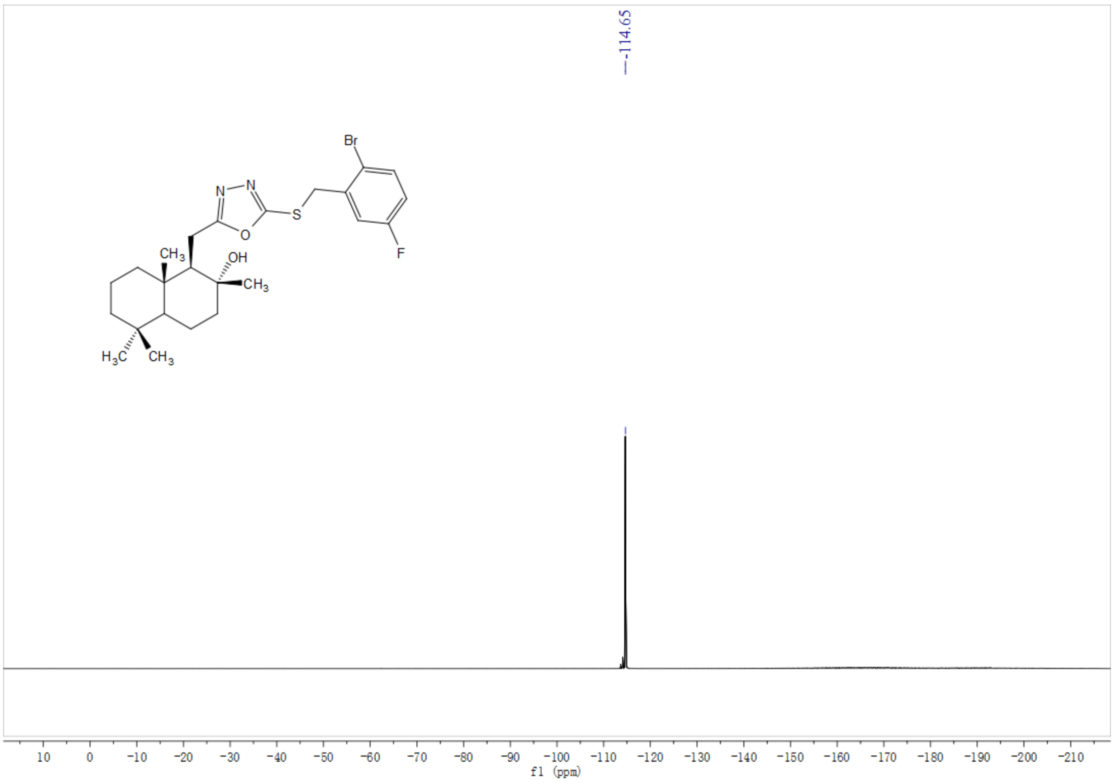

Figure S26 ^{19}F NMR Spectrum of **H9**

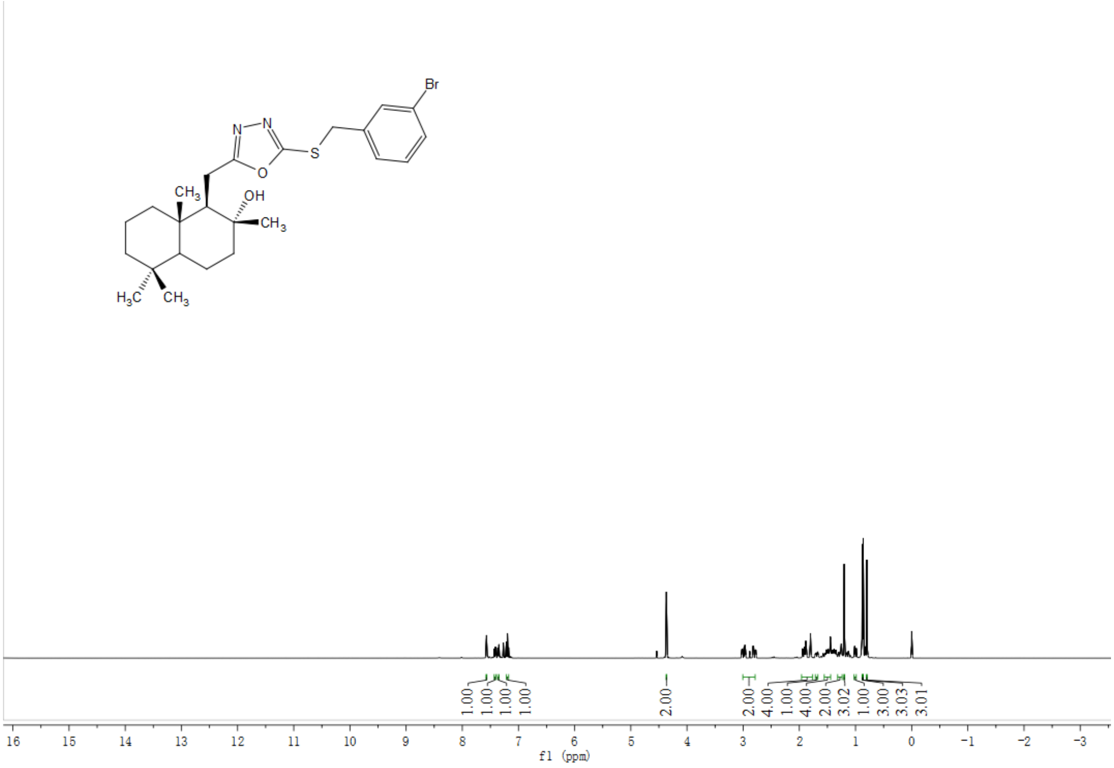

Figure S27 ^{1}H NMR Spectrum of **H10**

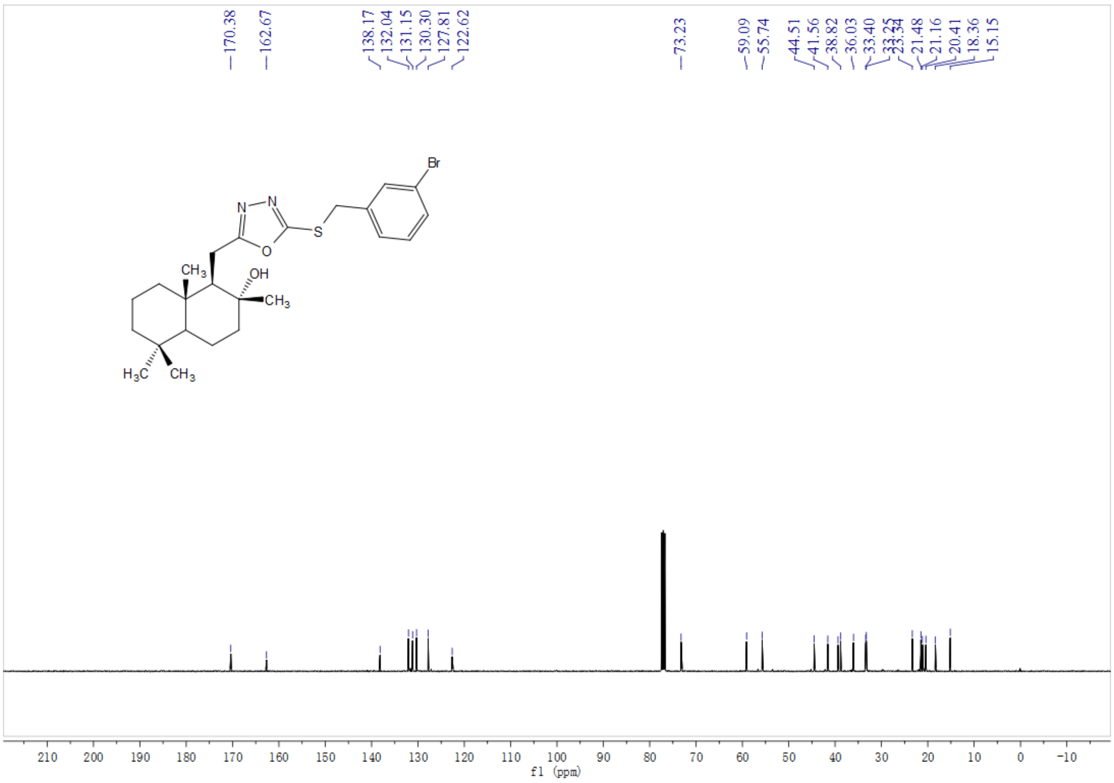

Figure S28 ^{13}C NMR Spectrum of **H10**

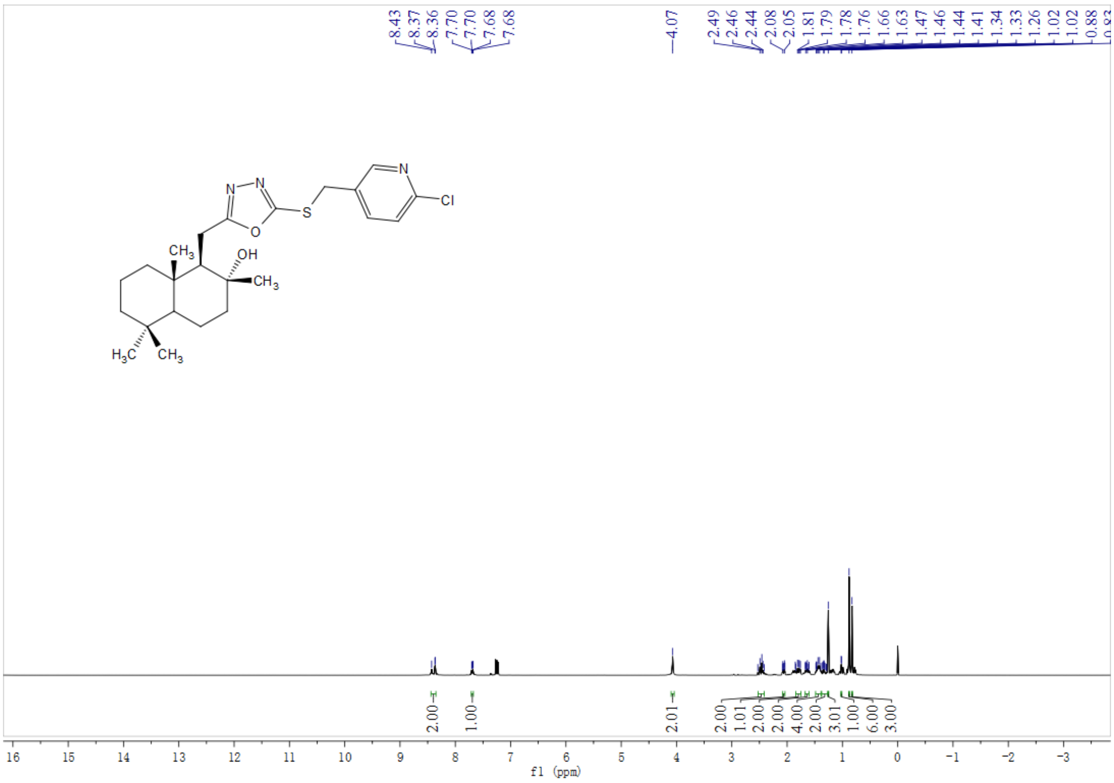

Figure S29 ^{1}H NMR Spectrum of **H11**

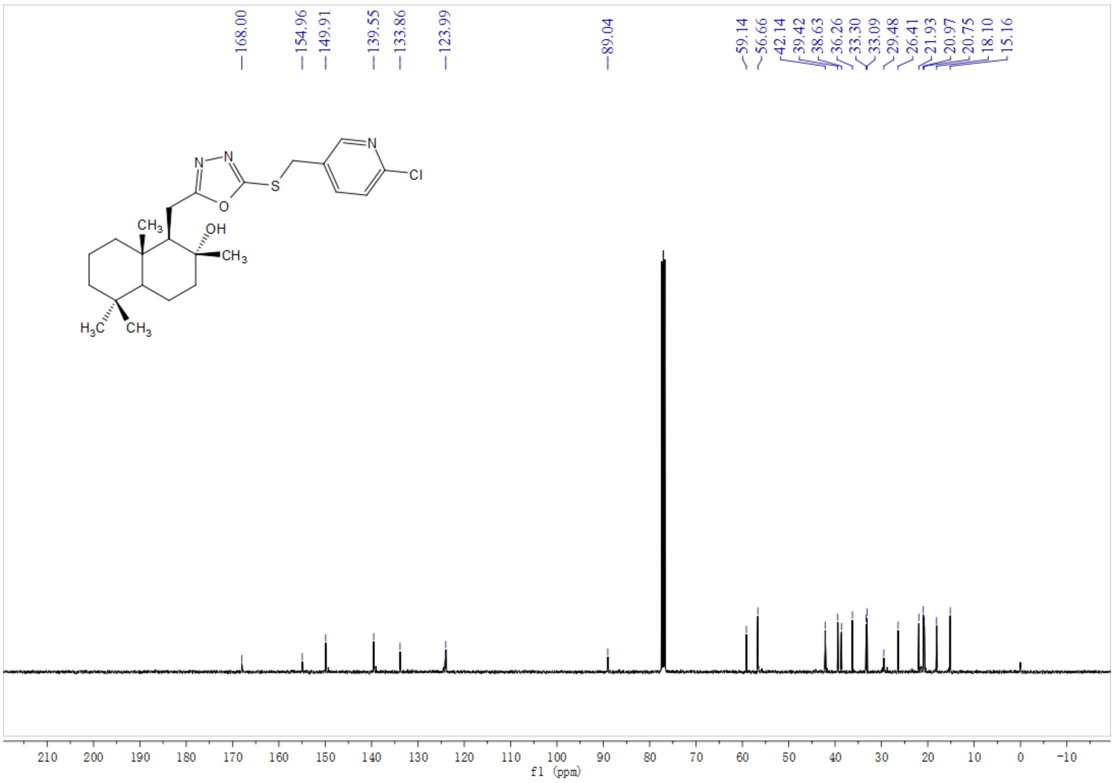

Figure S30 ^{13}C NMR Spectrum of **H11**

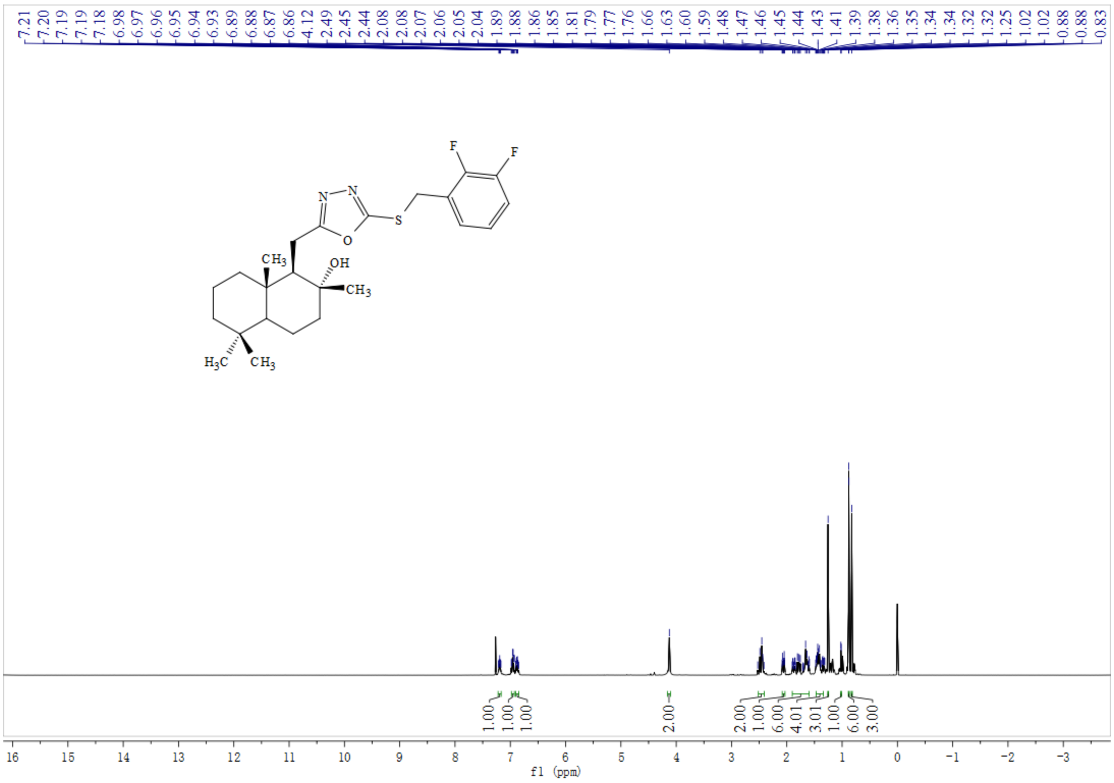

Figure S31 ^{1}H NMR Spectrum of **H12**

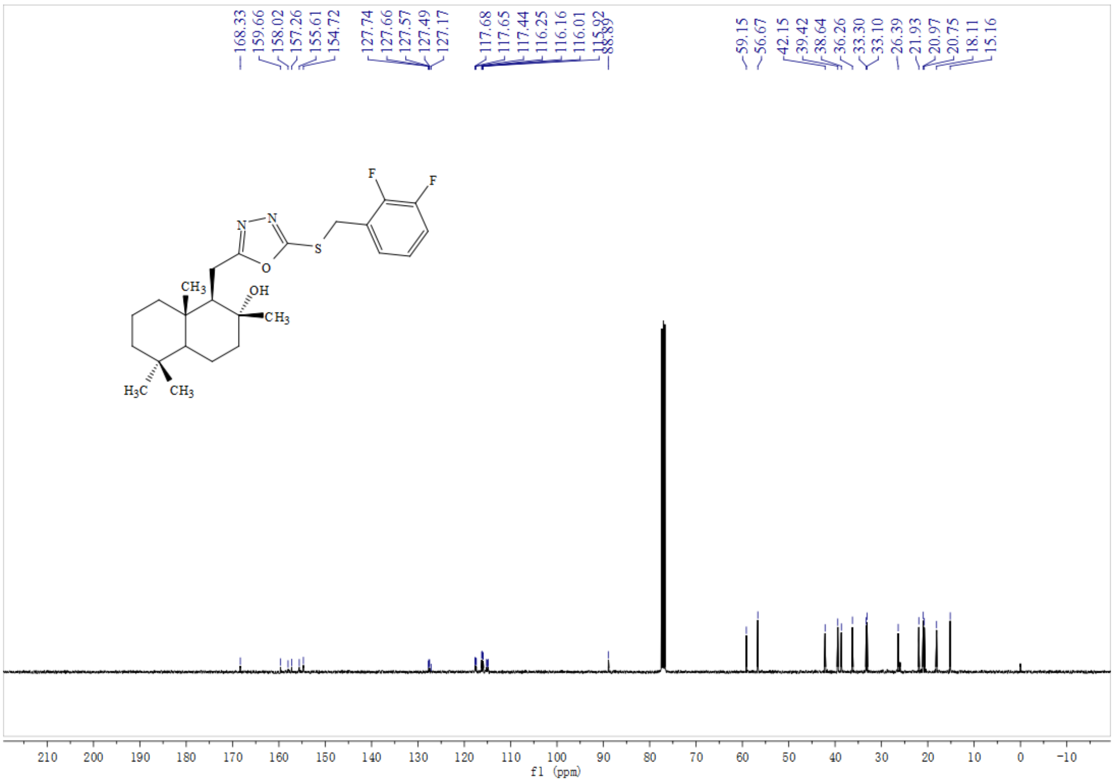

Figure S32 ^{13}C NMR Spectrum of **H12**

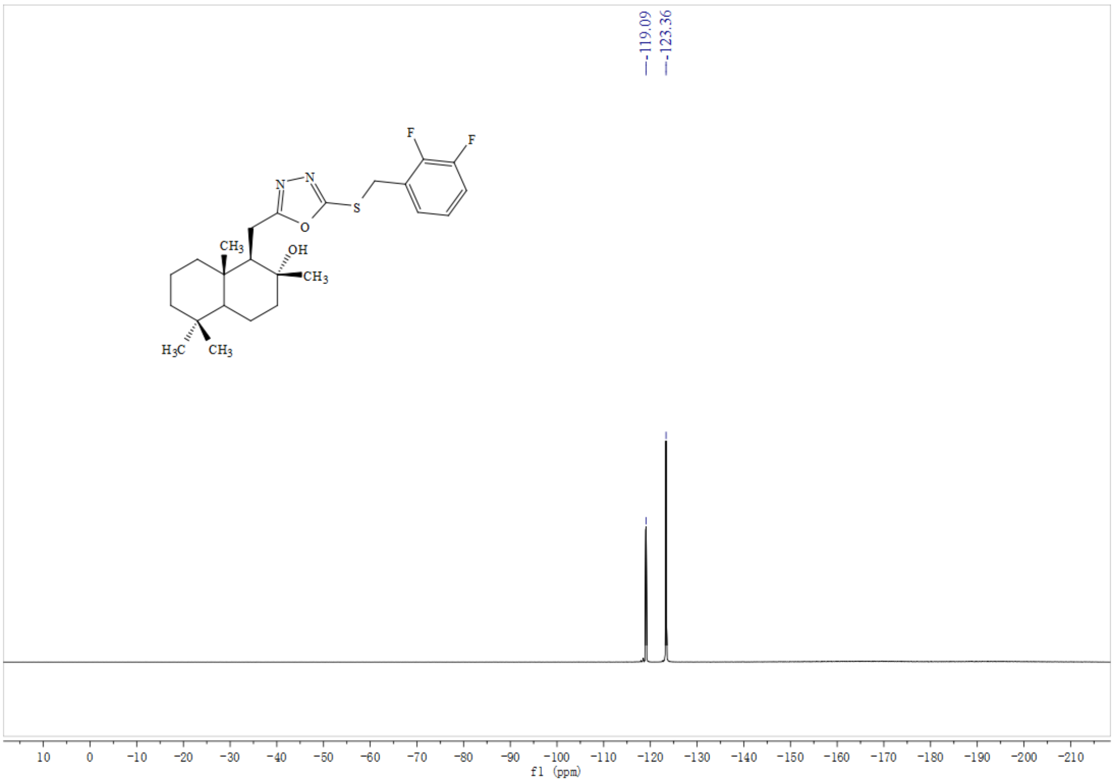

Figure S33 ^{19}F NMR Spectrum of **H12**

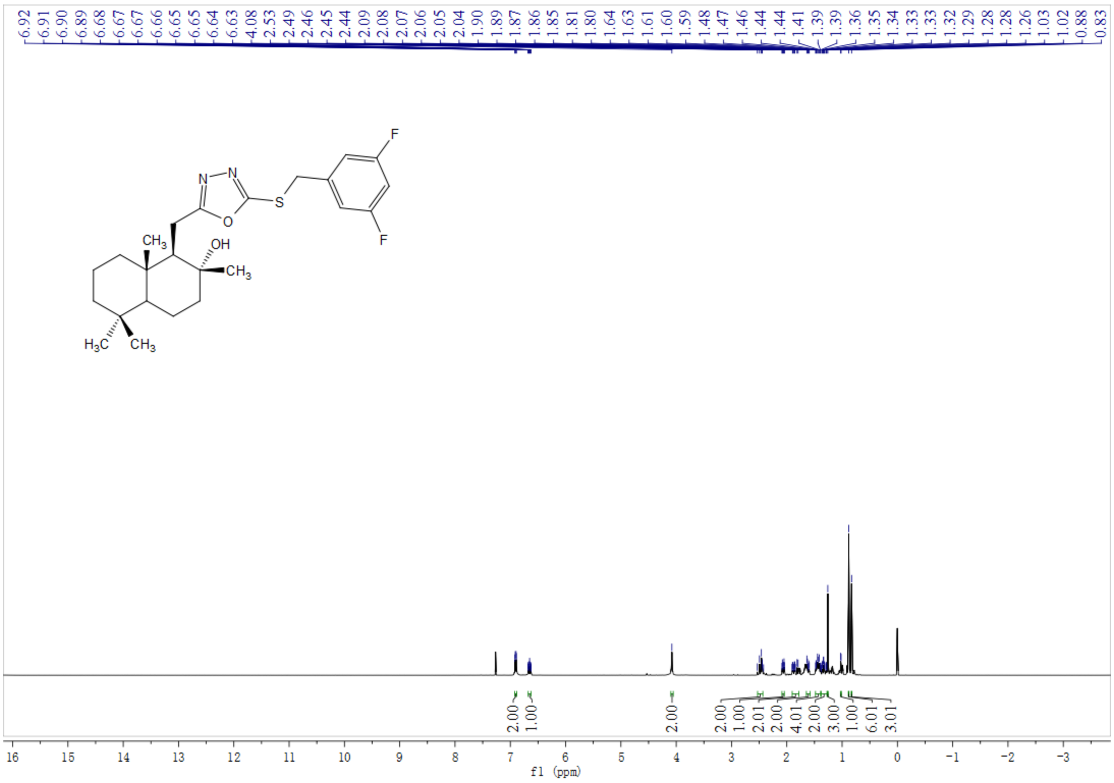

Figure S34 ^{1}H NMR Spectrum of **H13**

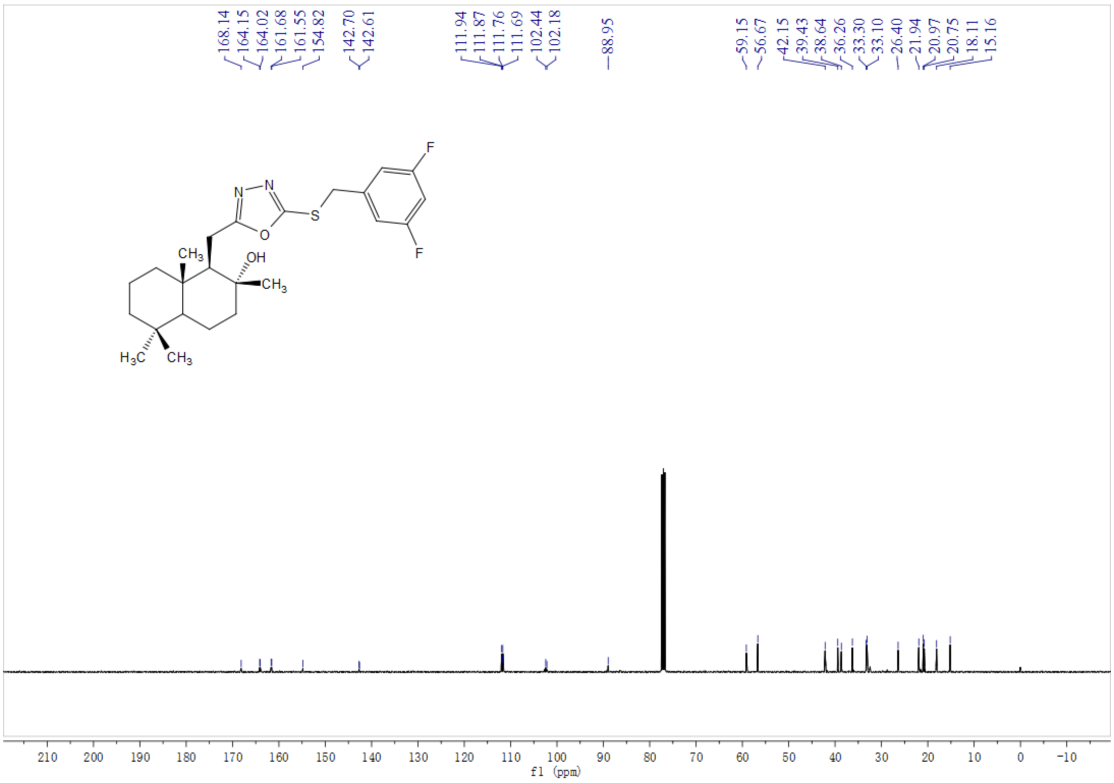

Figure S35 ^{13}C NMR Spectrum of **H13**

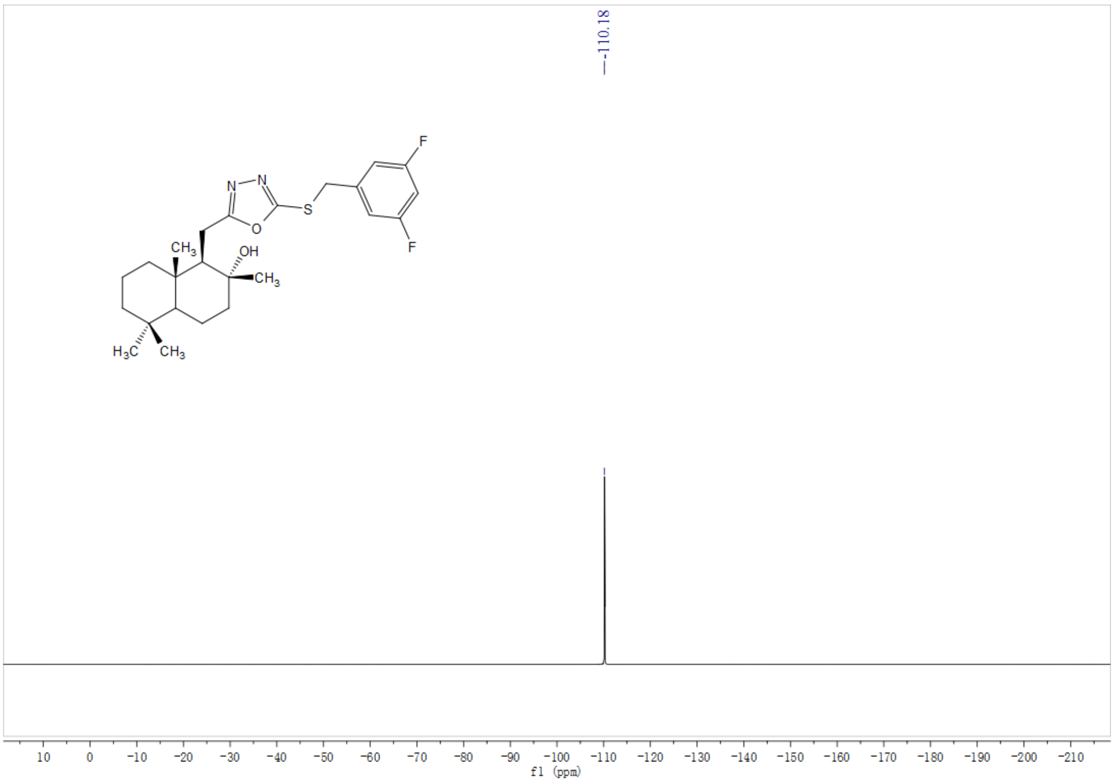

Figure S36 ^{19}F NMR Spectrum of **H13**

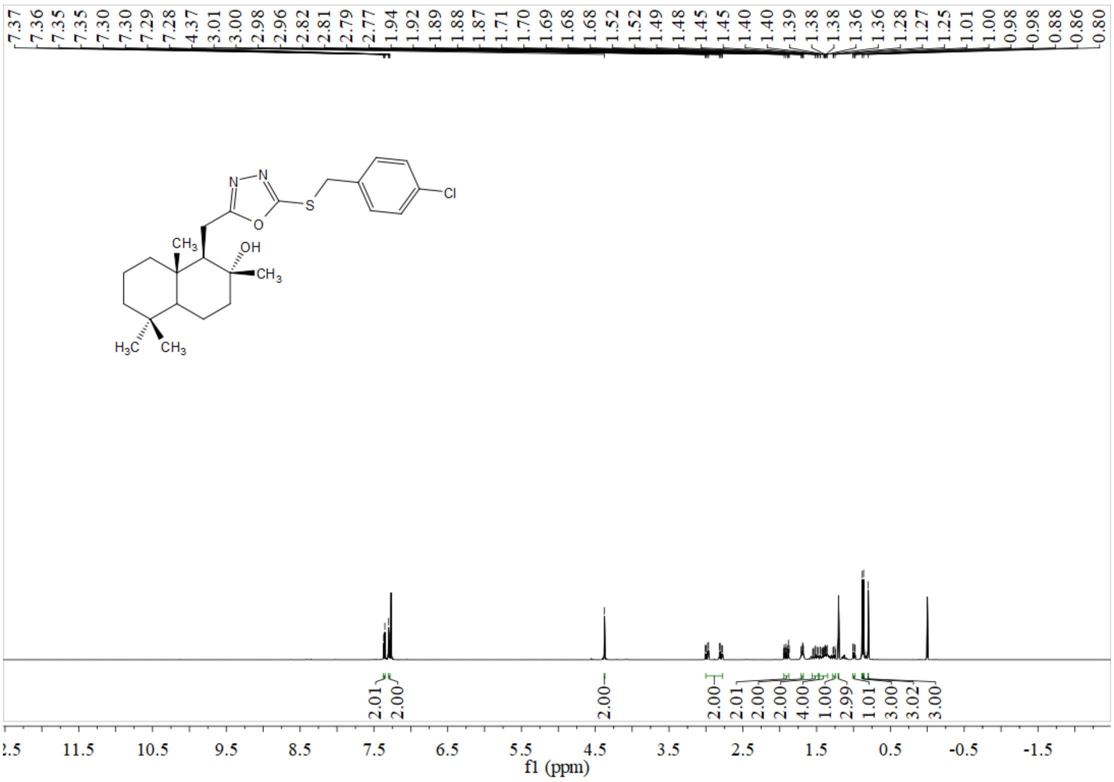

Figure S37 ^{1}H NMR Spectrum of **H14**

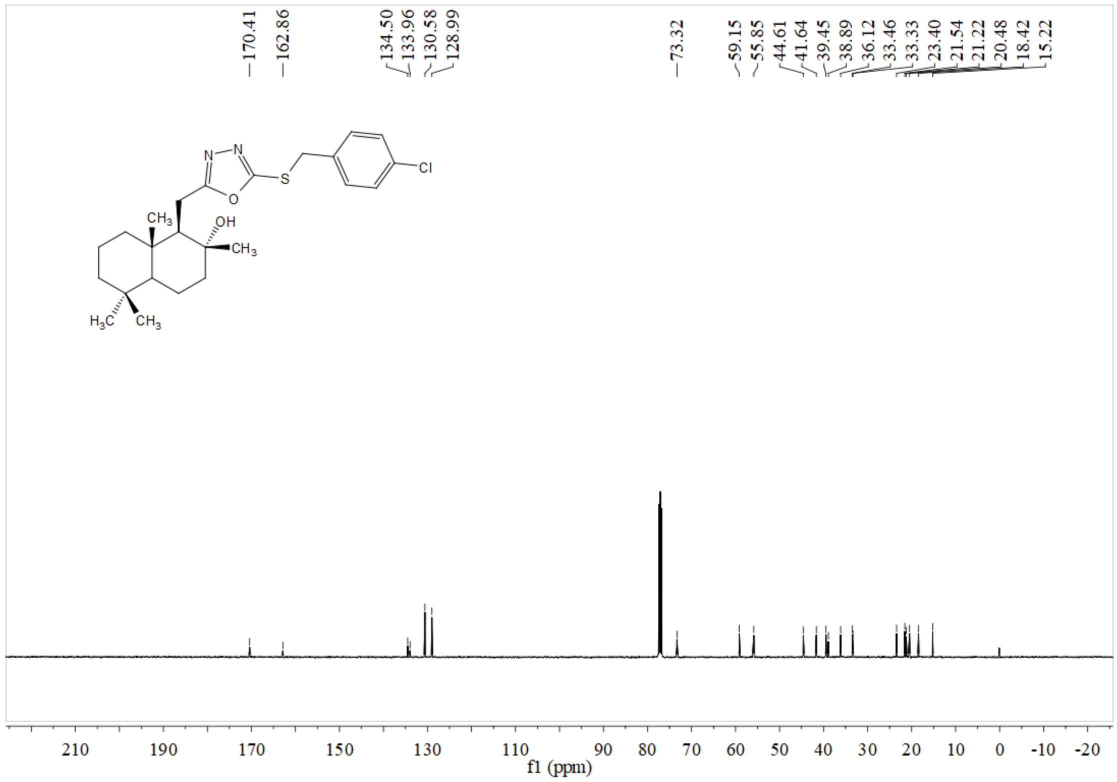

Figure S38 ^{13}C NMR Spectrum of **H14**

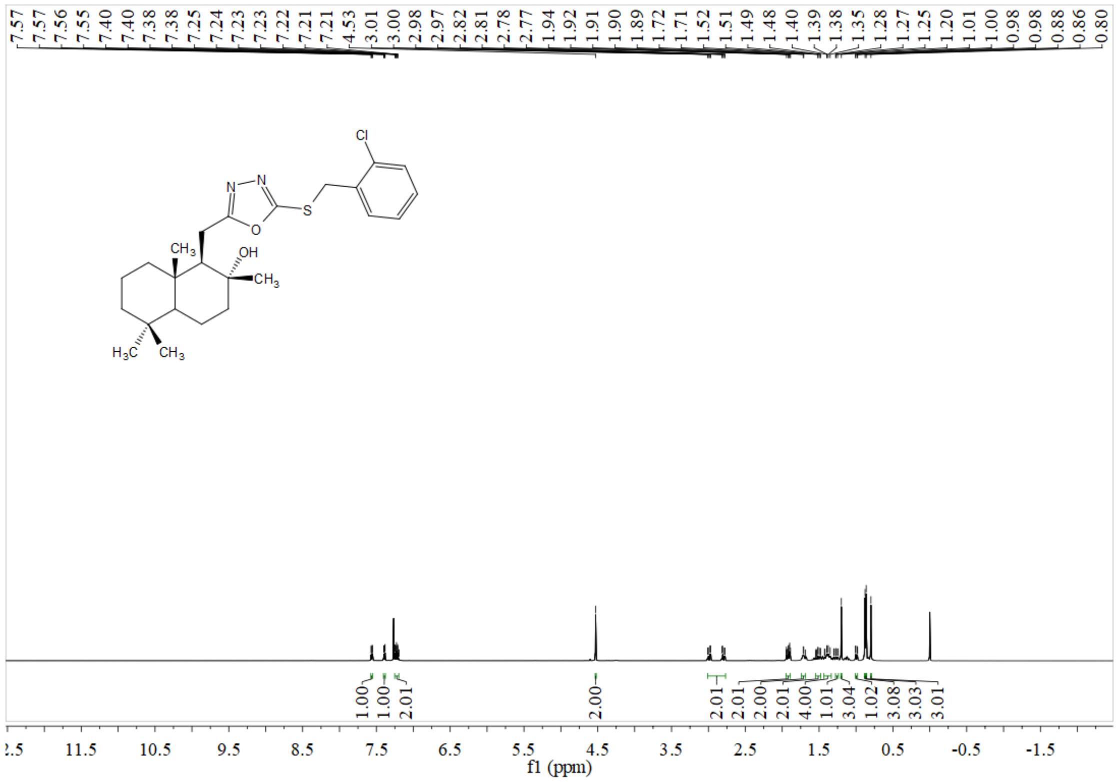

Figure S39 ^{1}H NMR Spectrum of **H15**

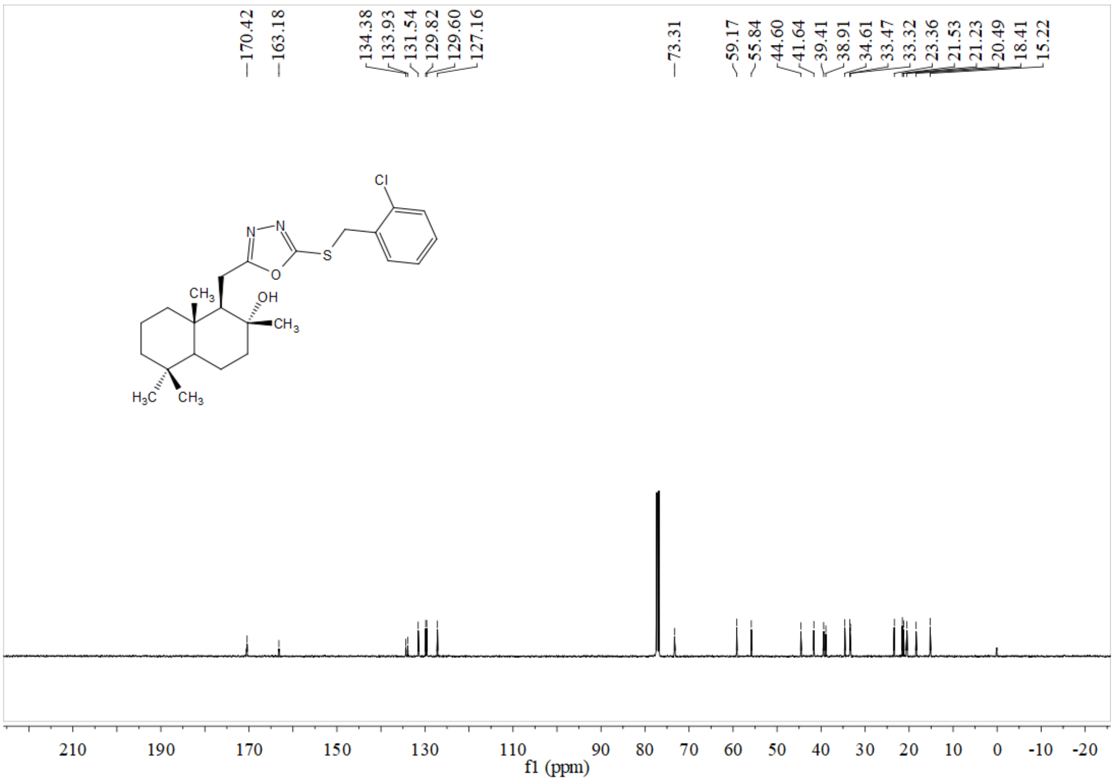

Figure S40 ^{13}C NMR Spectrum of **H15**

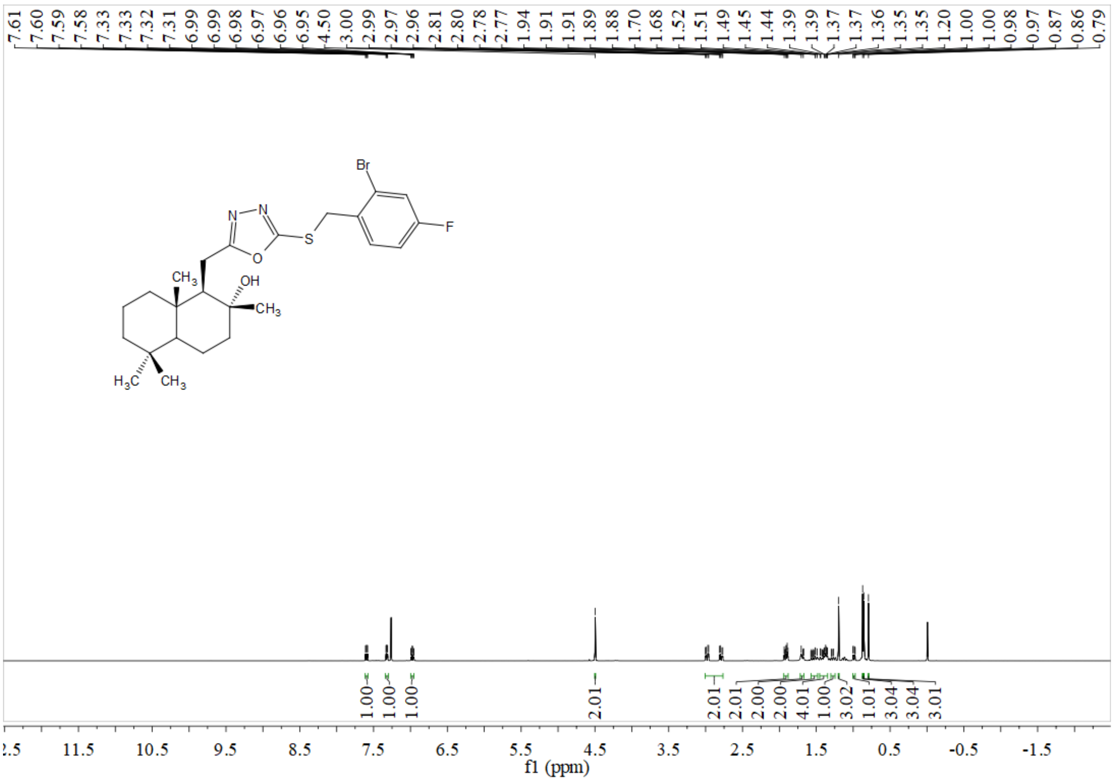

Figure S41 ^{1}H NMR Spectrum of **H16**

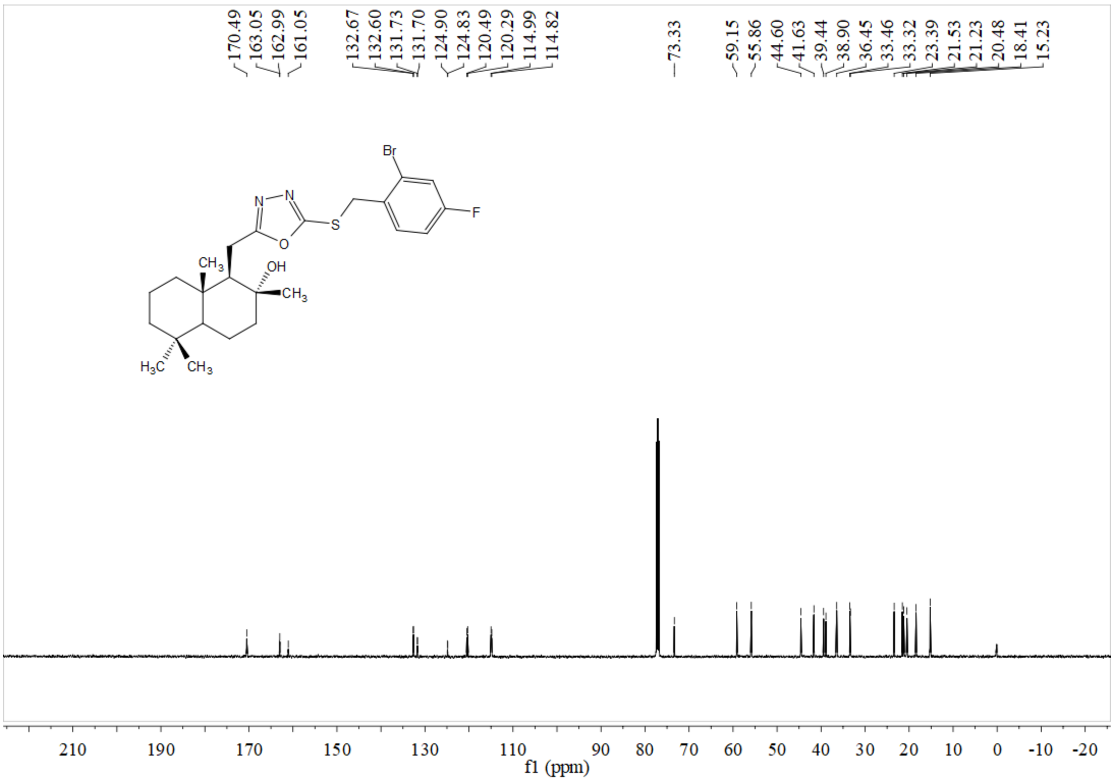

Figure S42 ^{13}C NMR Spectrum of **H16**

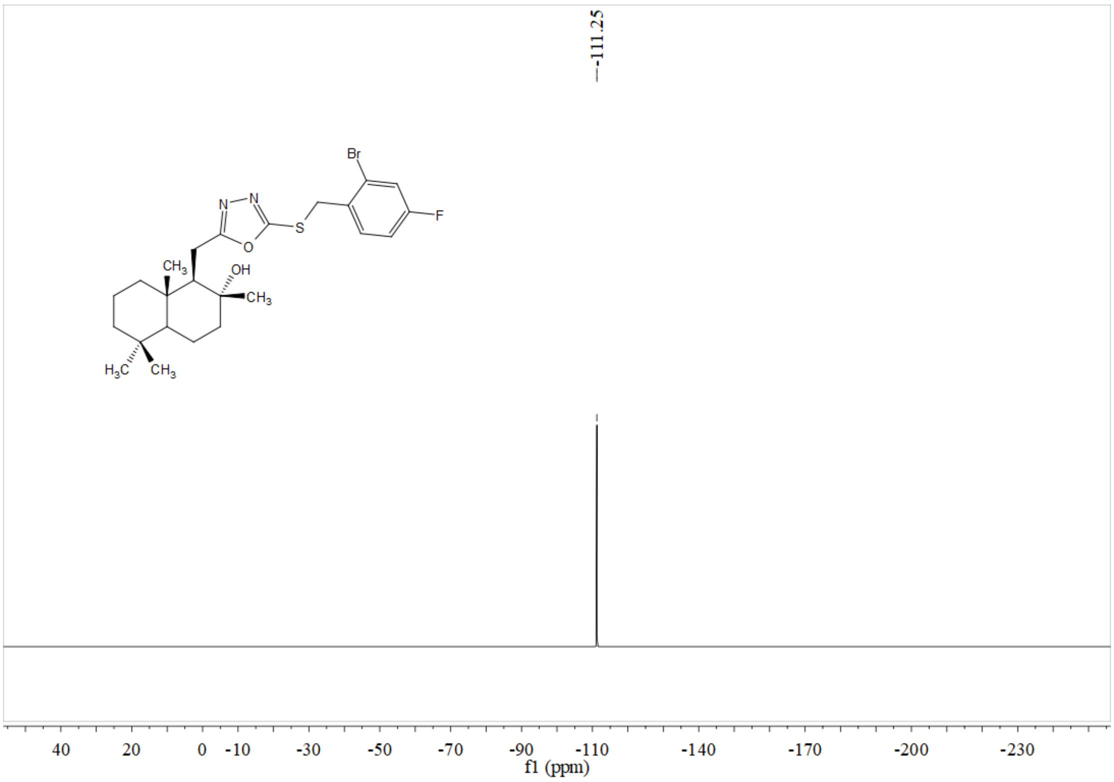

Figure S43 ^{19}F NMR Spectrum of **H16**

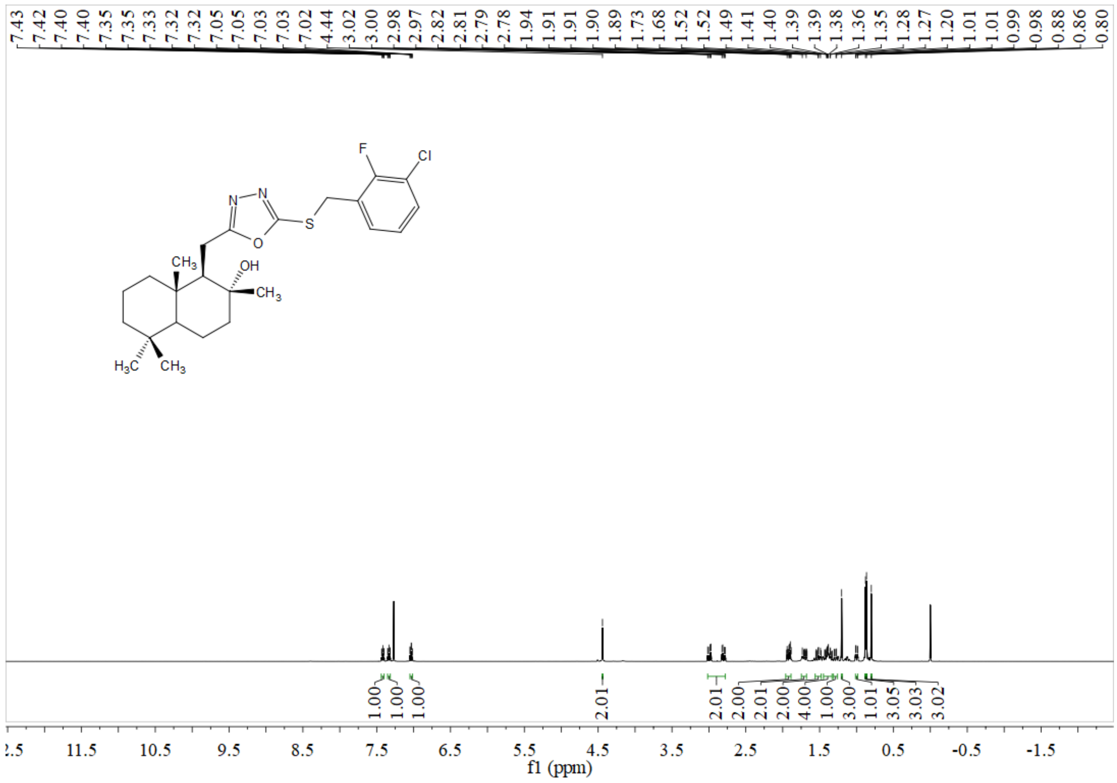

Figure S44 ^{1}H NMR Spectrum of **H17**

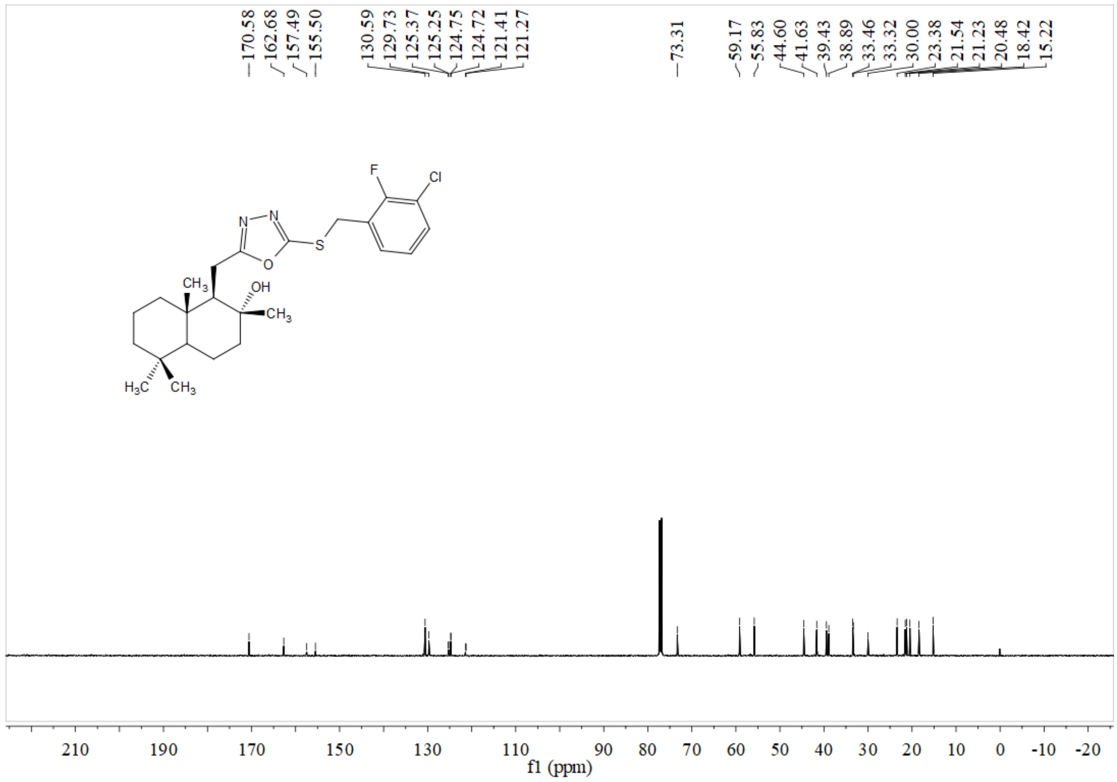

Figure S45 ^{13}C NMR Spectrum of **H17**

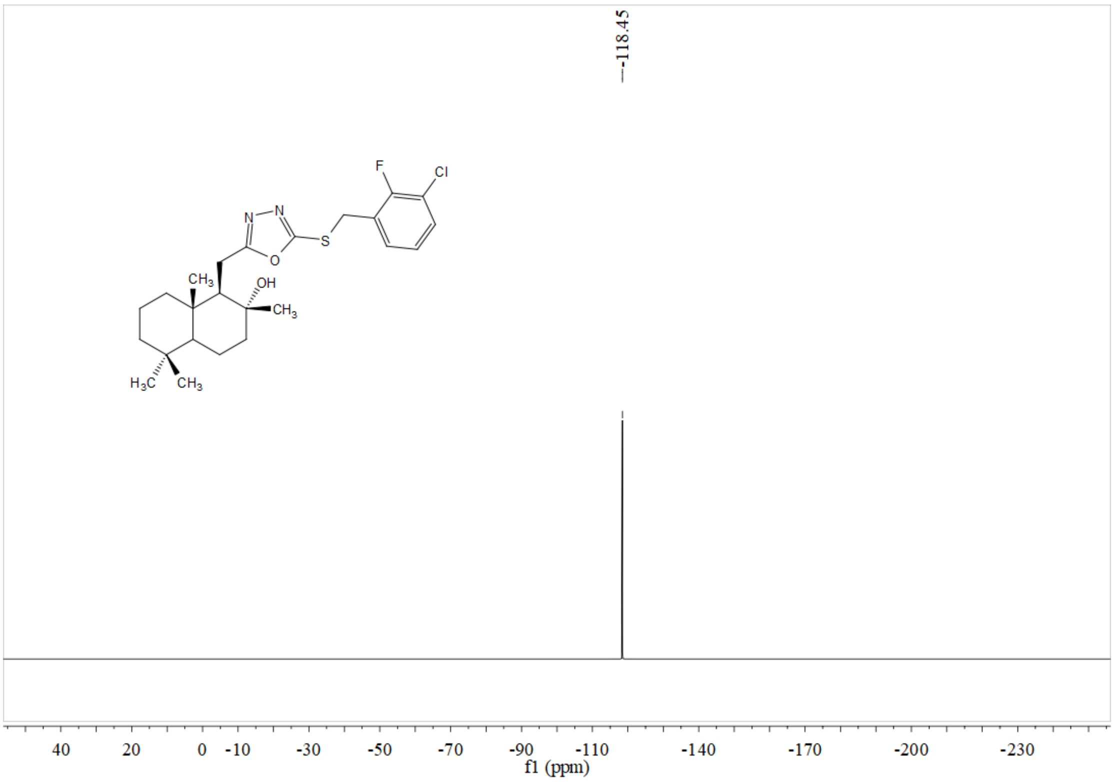

Figure S46 ^{19}F NMR Spectrum of **H13**

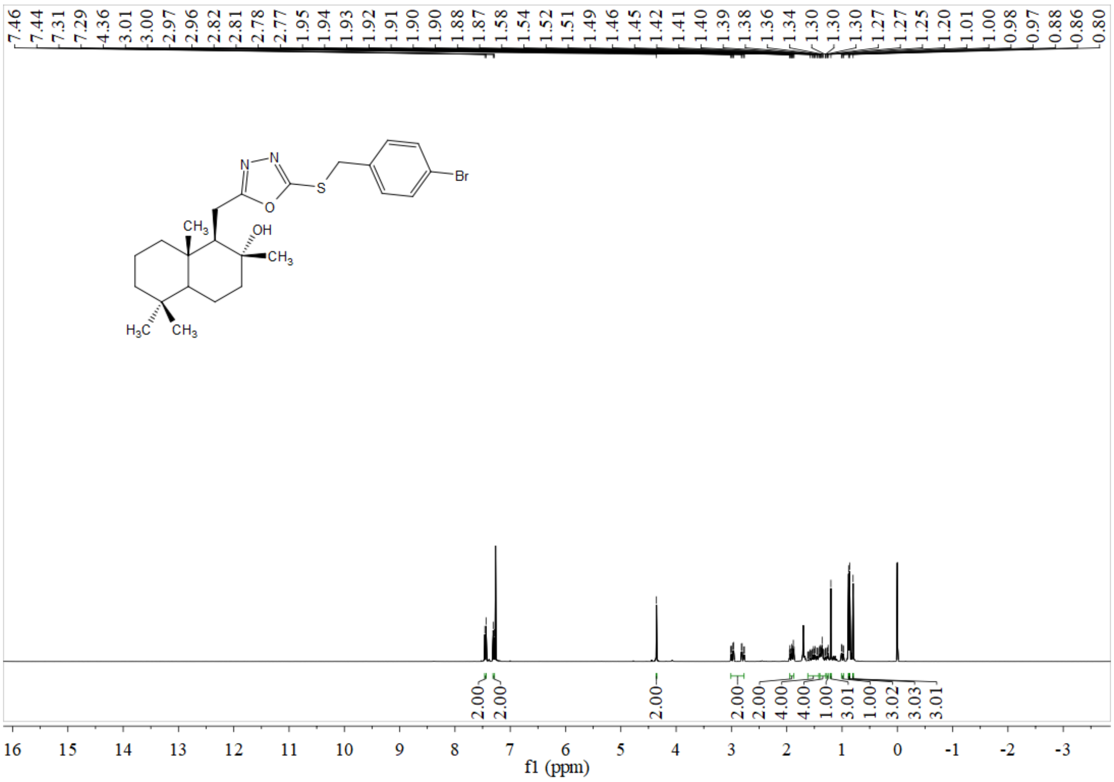

Figure S47 ^{1}H NMR Spectrum of **H18**

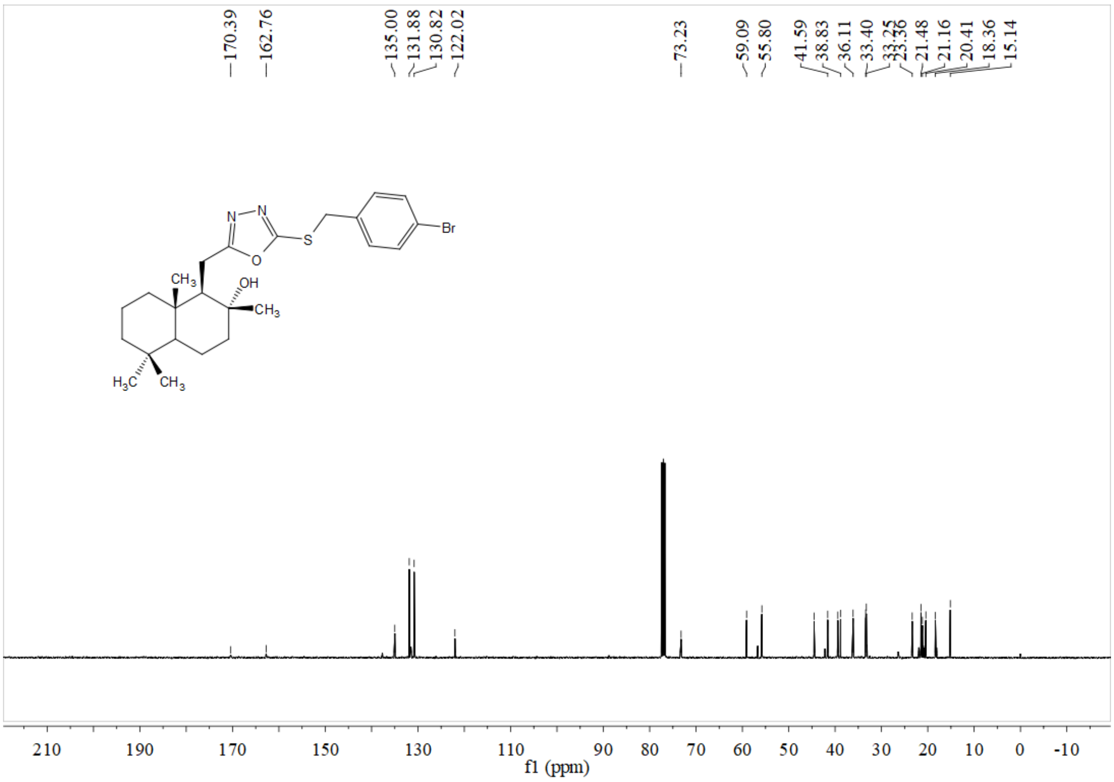

Figure S48 ^{13}C NMR Spectrum of **H18**

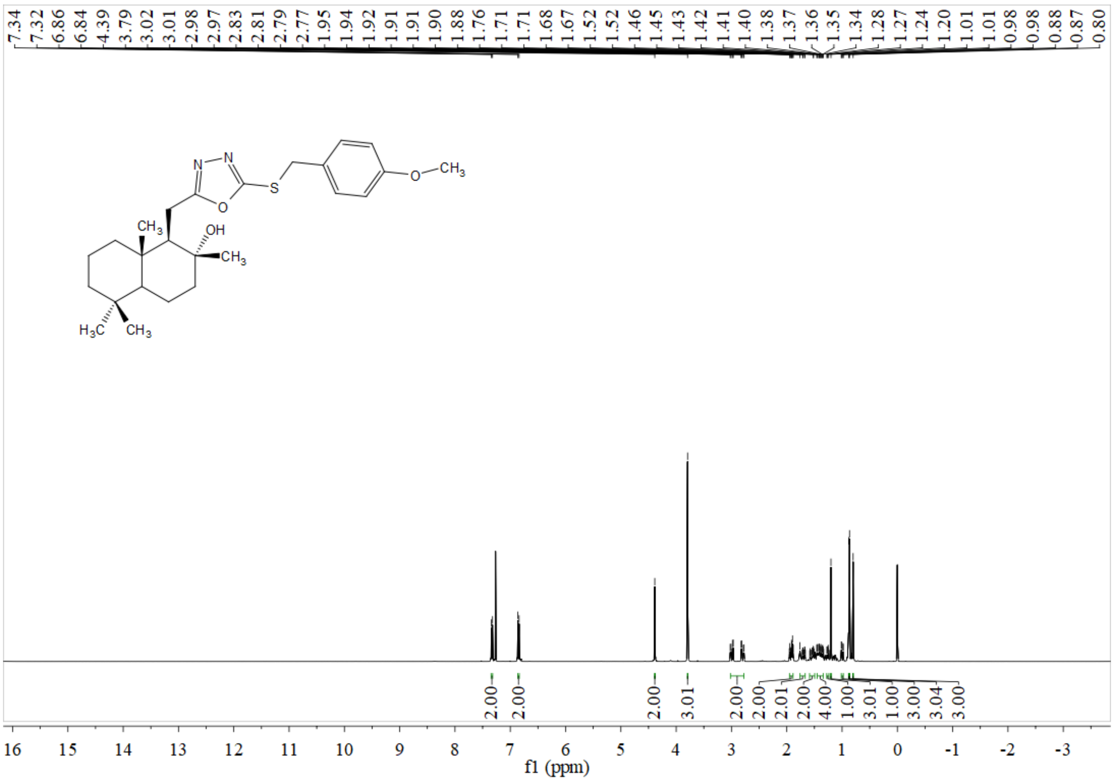

Figure S49 ^{1}H NMR Spectrum of **H19**

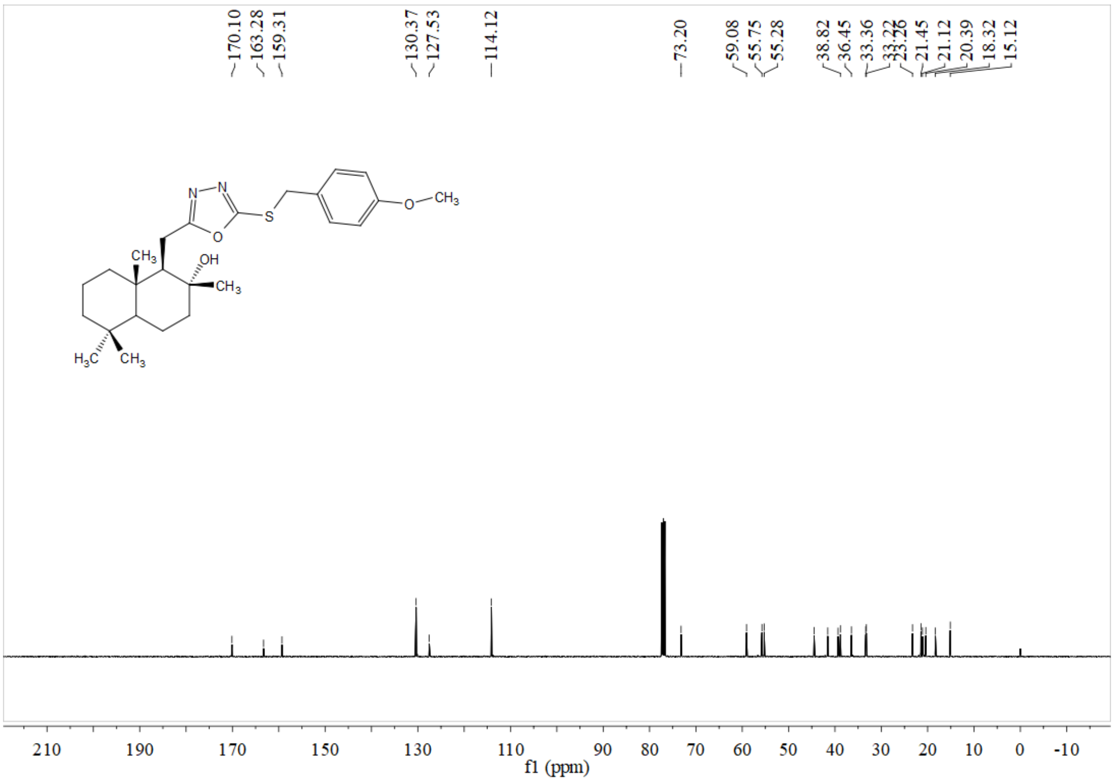

Figure S50 ^{13}C NMR Spectrum of **H19**

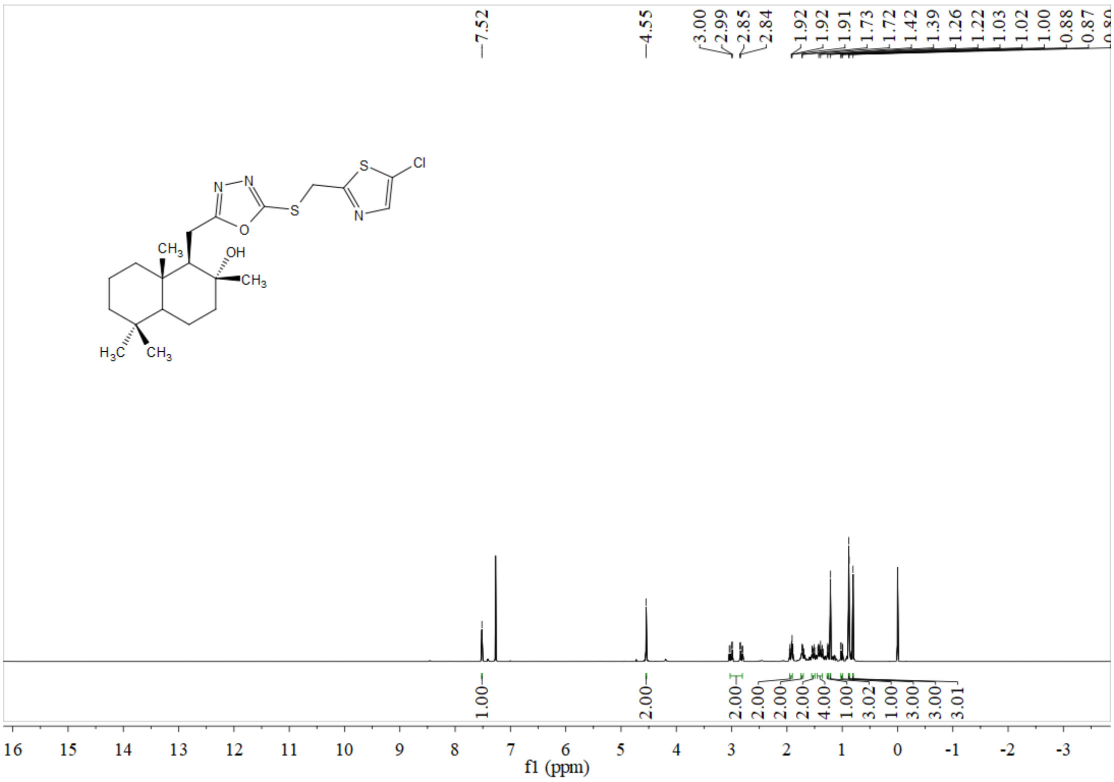

Figure S51 ^{1}H NMR Spectrum of **H20**

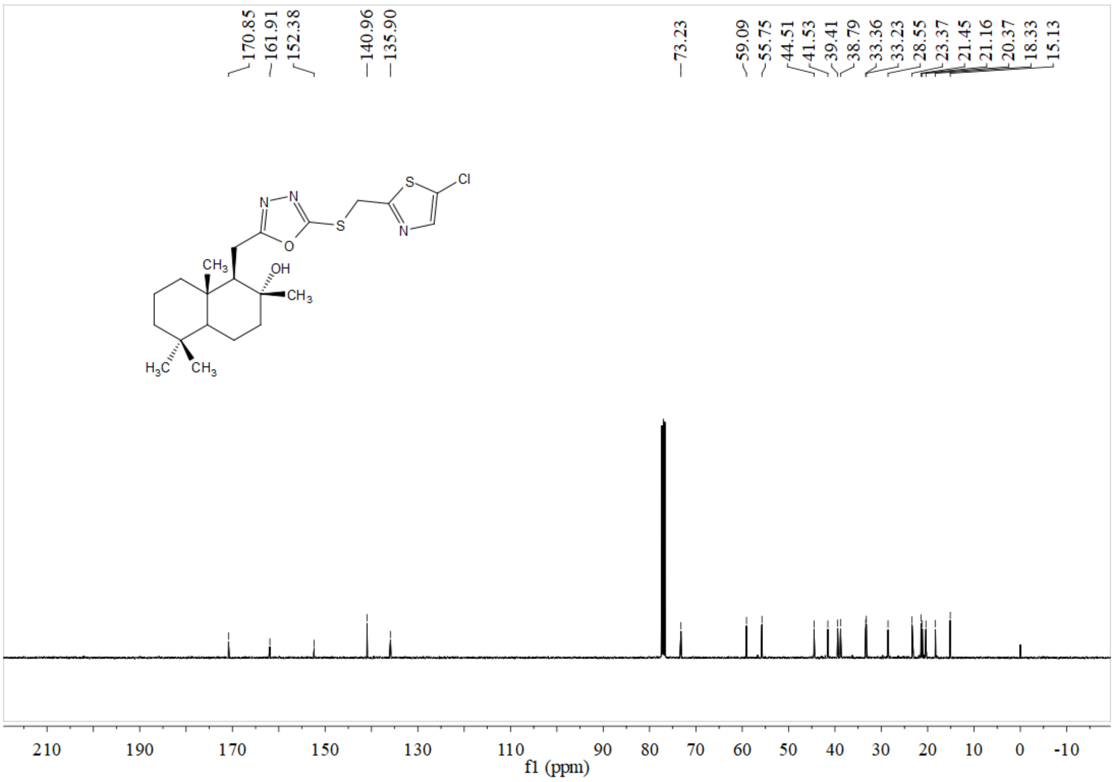

Figure S52 ^{13}C NMR Spectrum of **H20**

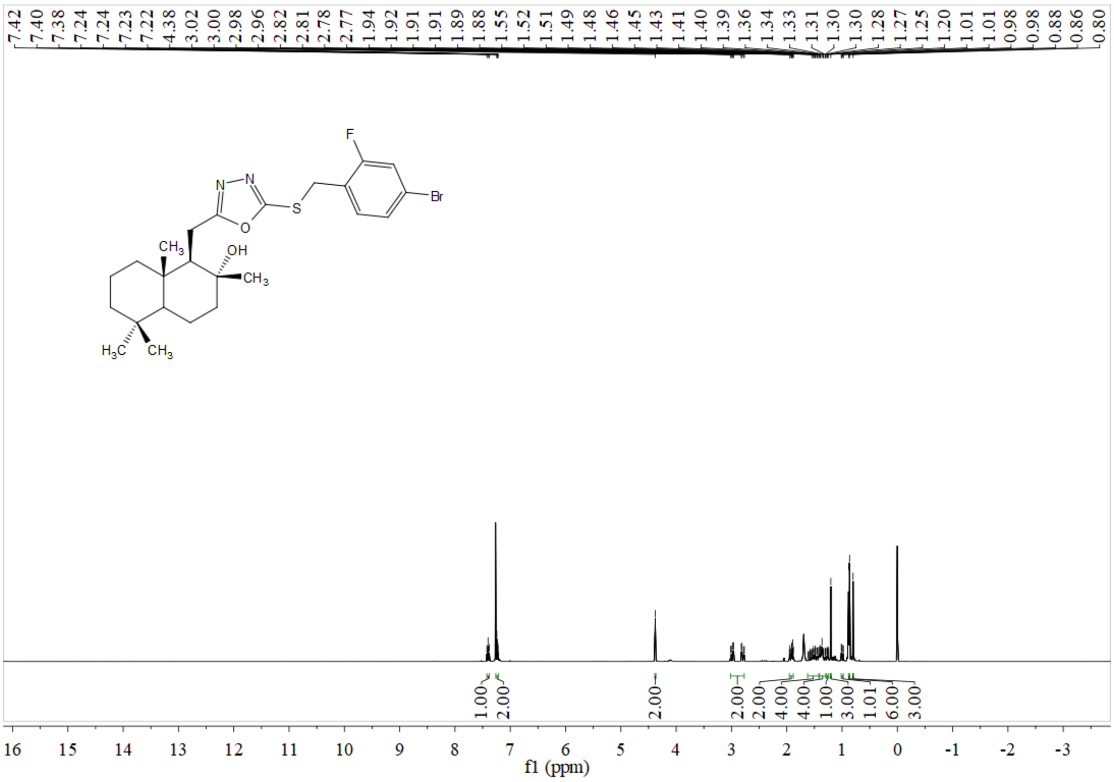

Figure S53 ^{1}H NMR Spectrum of **H21**

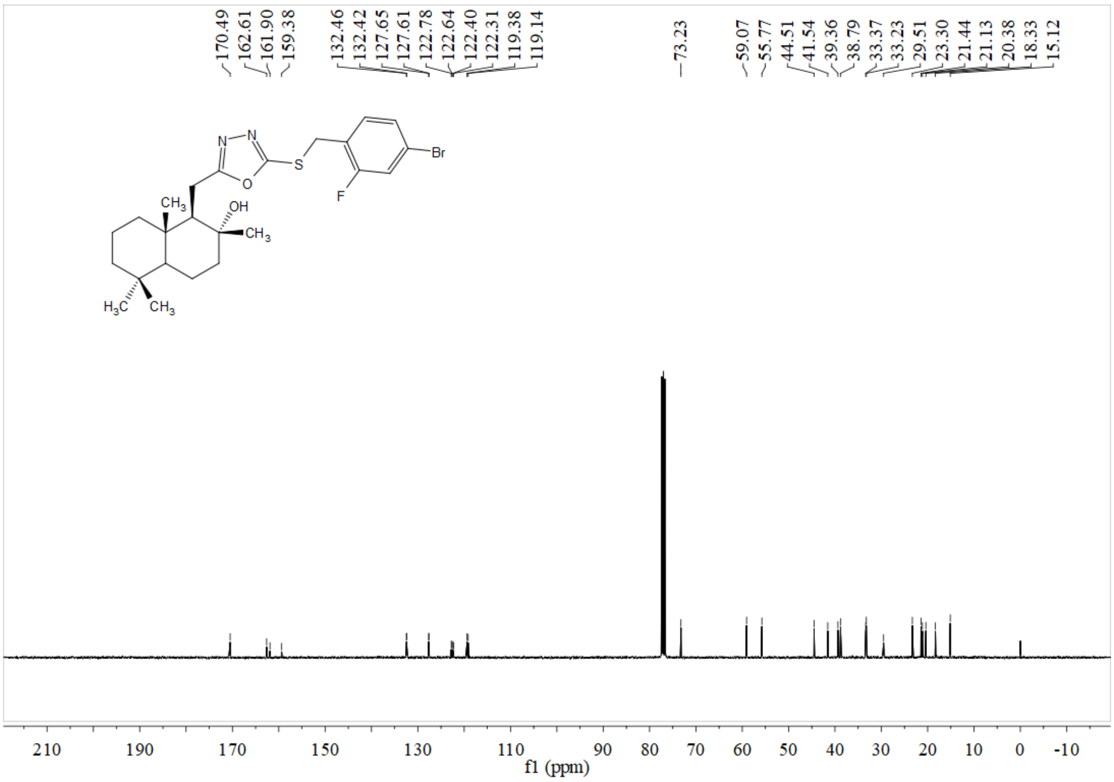

Figure S54 ^{13}C NMR Spectrum of **H21**

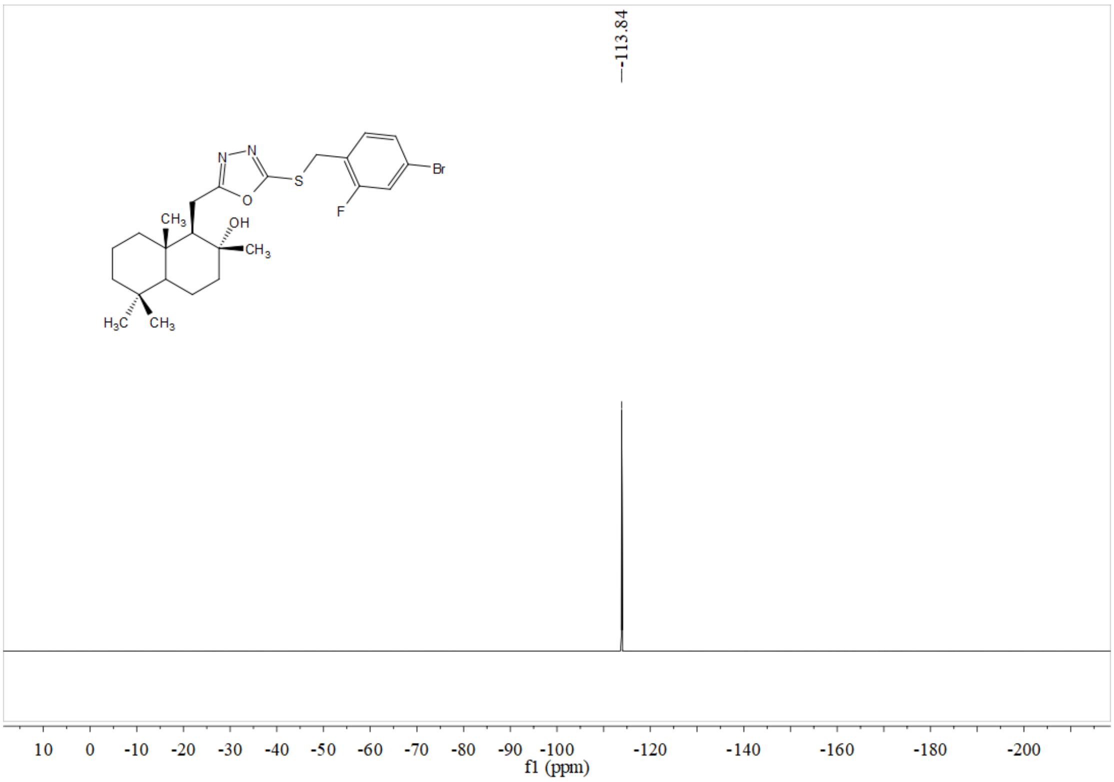

Figure S55 ^{19}F NMR Spectrum of **H21**

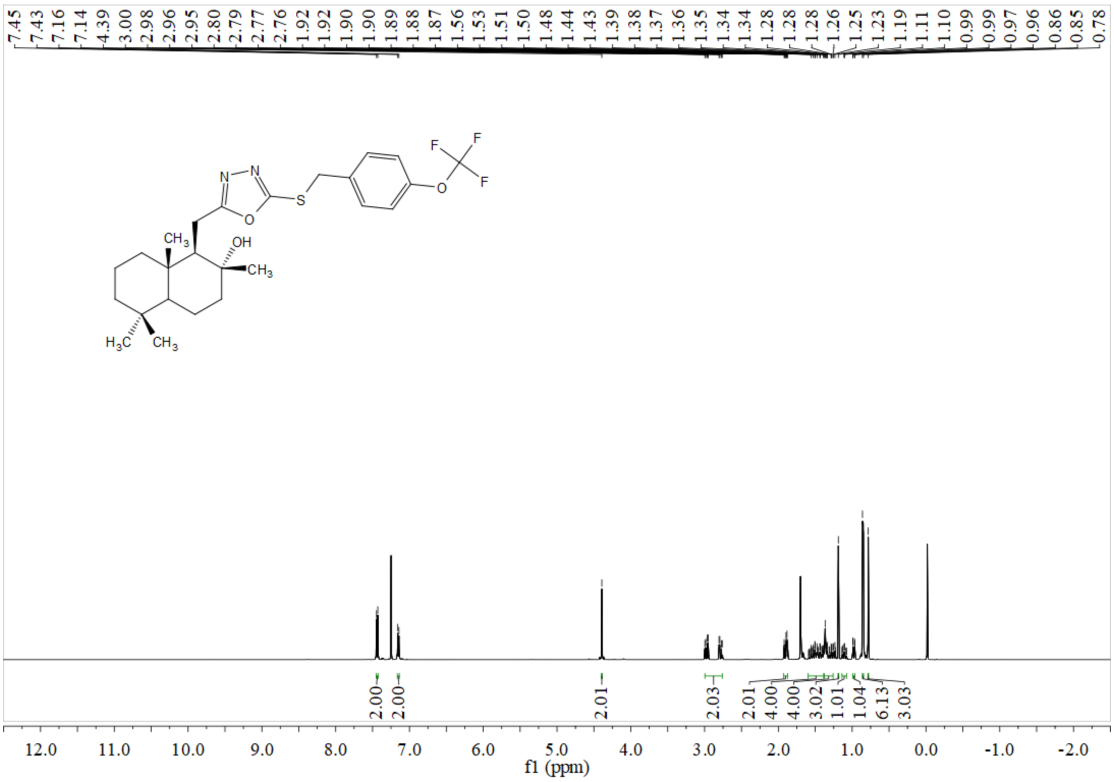

Figure S56 ^{1}H NMR Spectrum of **H22**

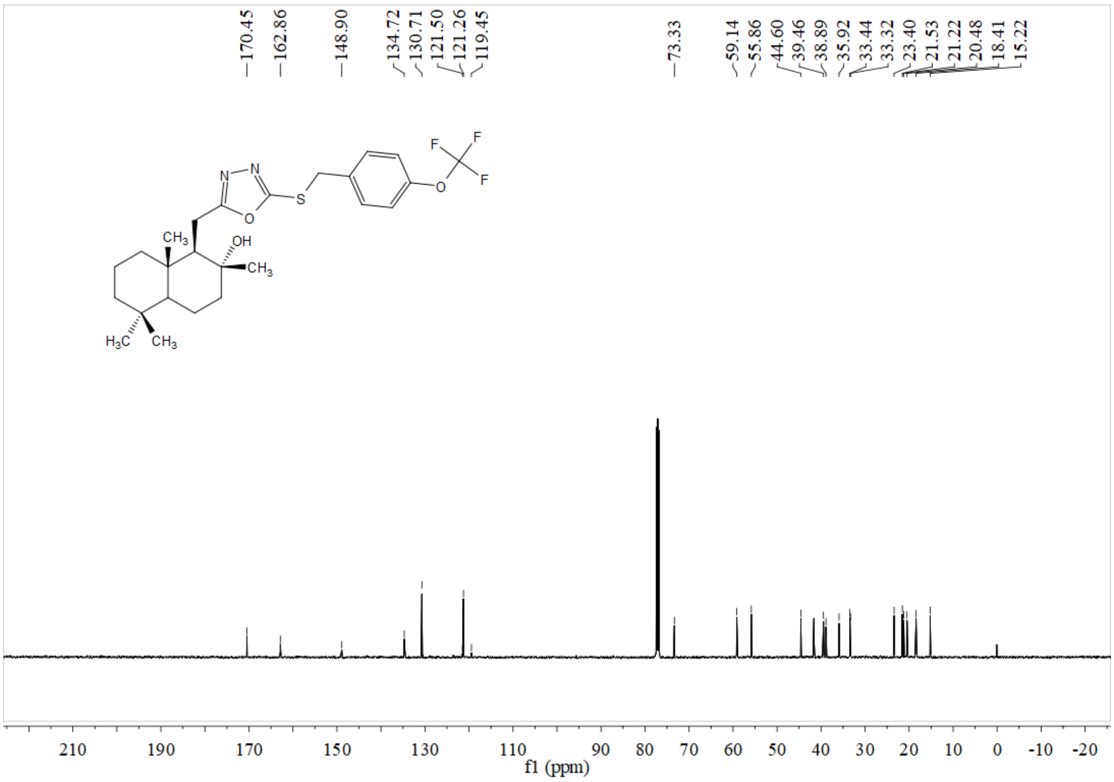

Figure S57 ^{13}C NMR Spectrum of **H22**

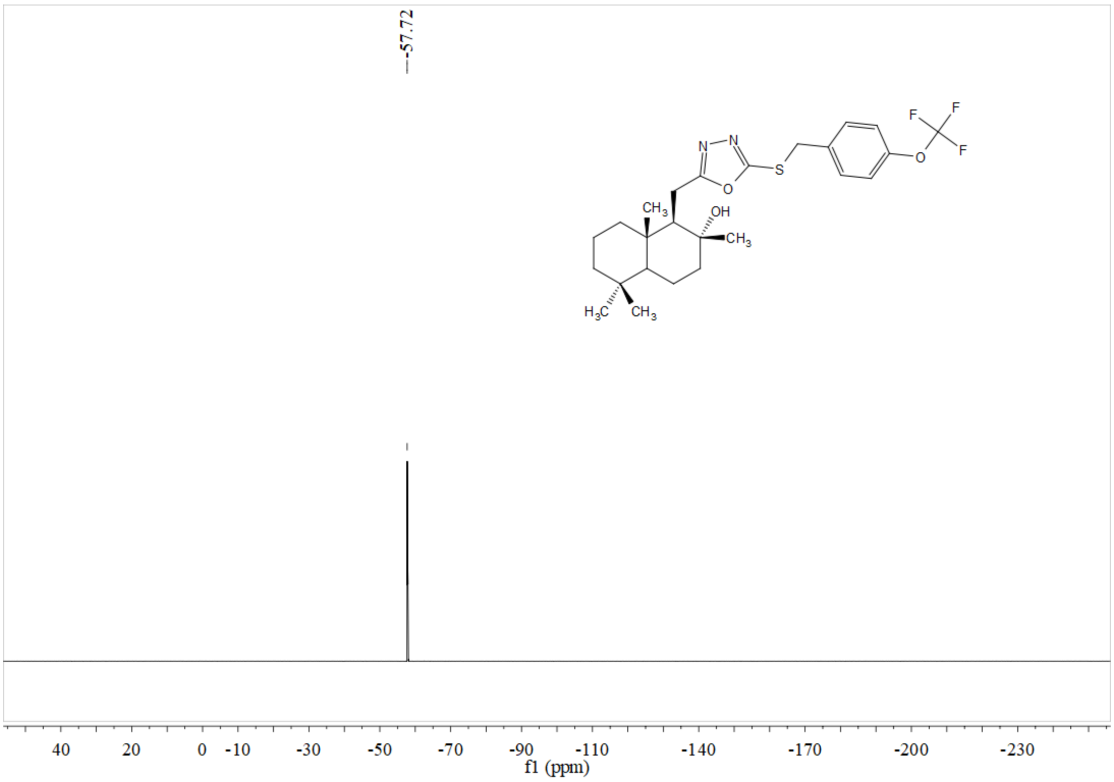

Figure S58 ^{19}F NMR Spectrum of **H22**

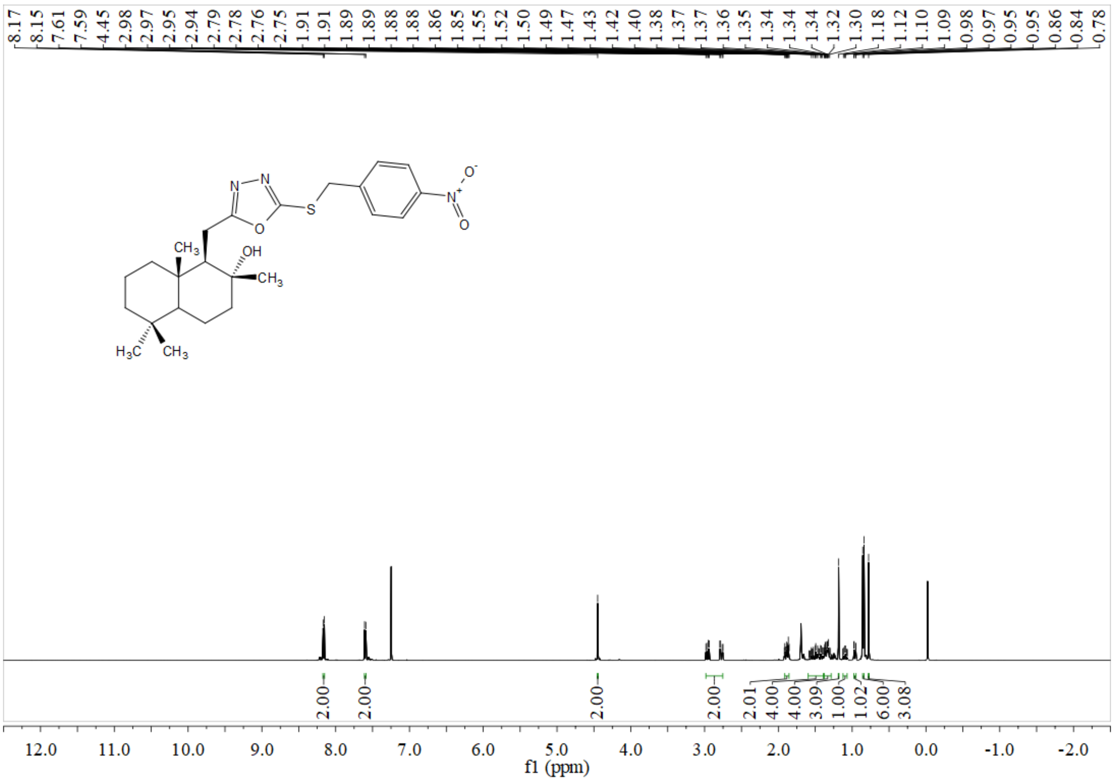

Figure S59 ^{1}H NMR Spectrum of **H23**

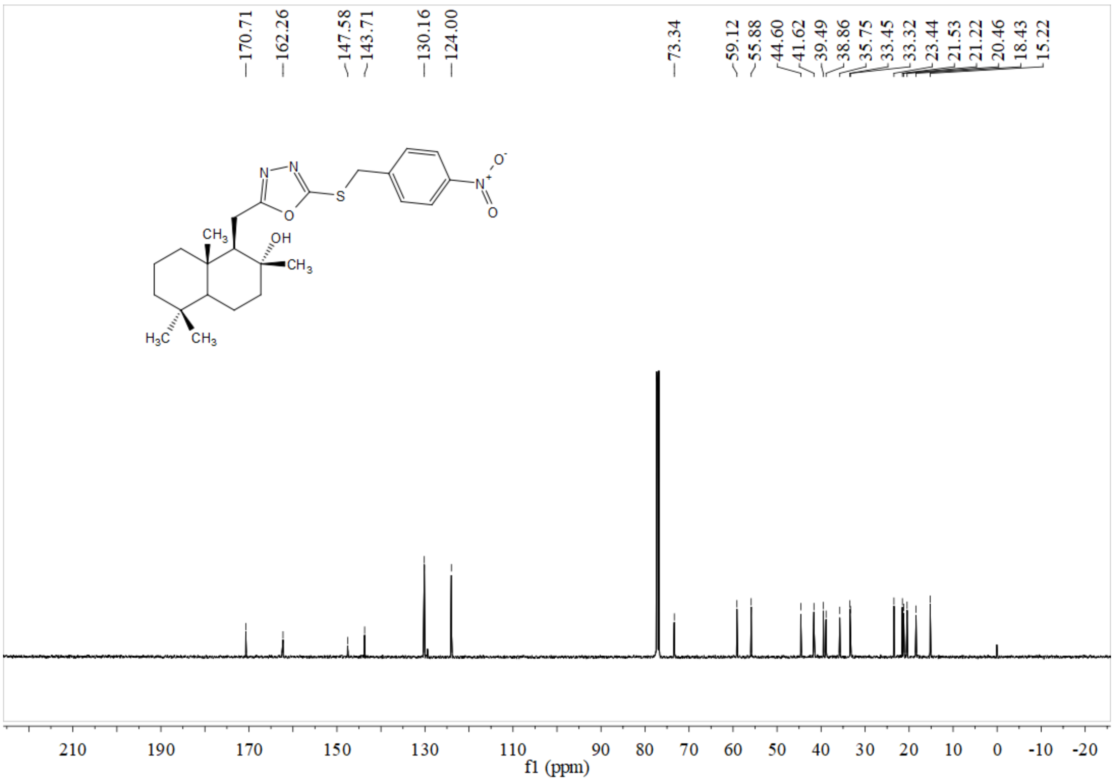

Figure S60 ^{13}C NMR Spectrum of **H23**

3. HRMS Spectra of the title compounds H1 - H23

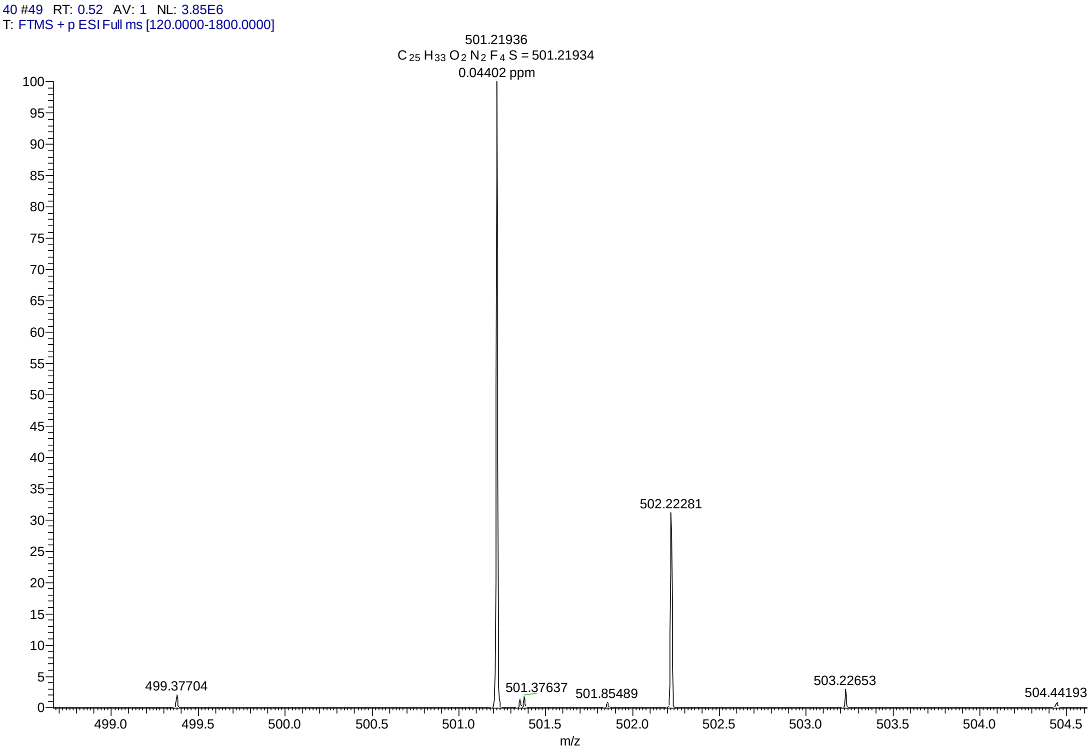

Figure S61 **H1**

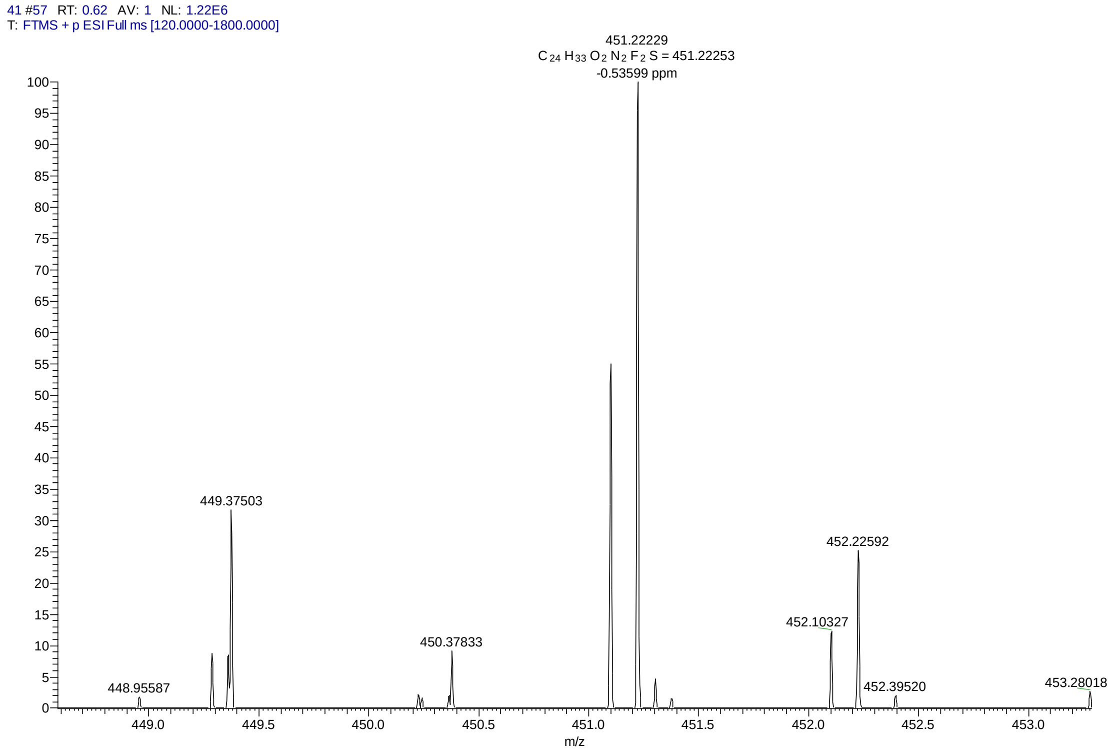

Figure S62 **H2**

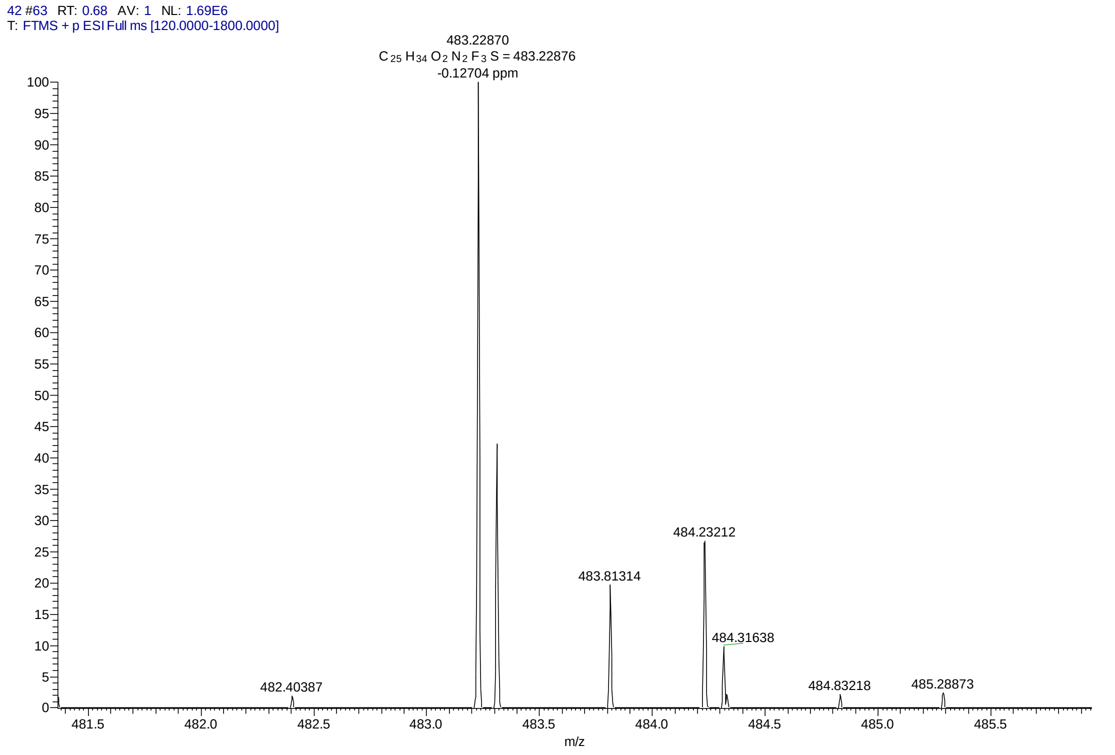

Figure S63 **H3**

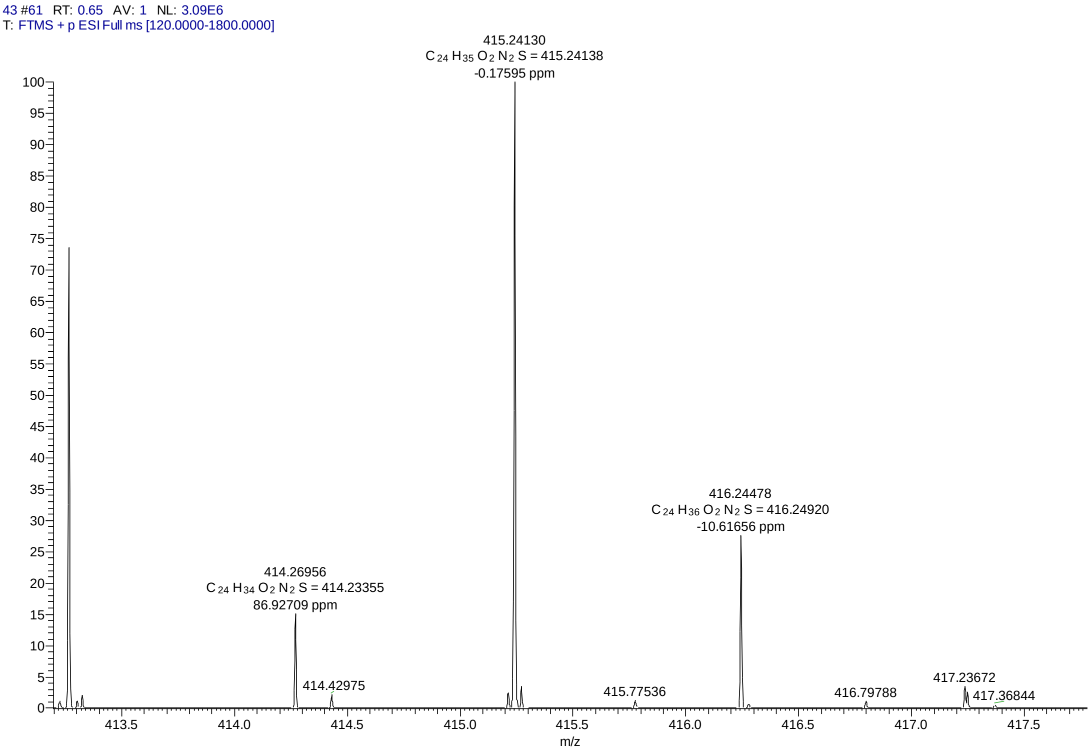

Figure S64 **H4**

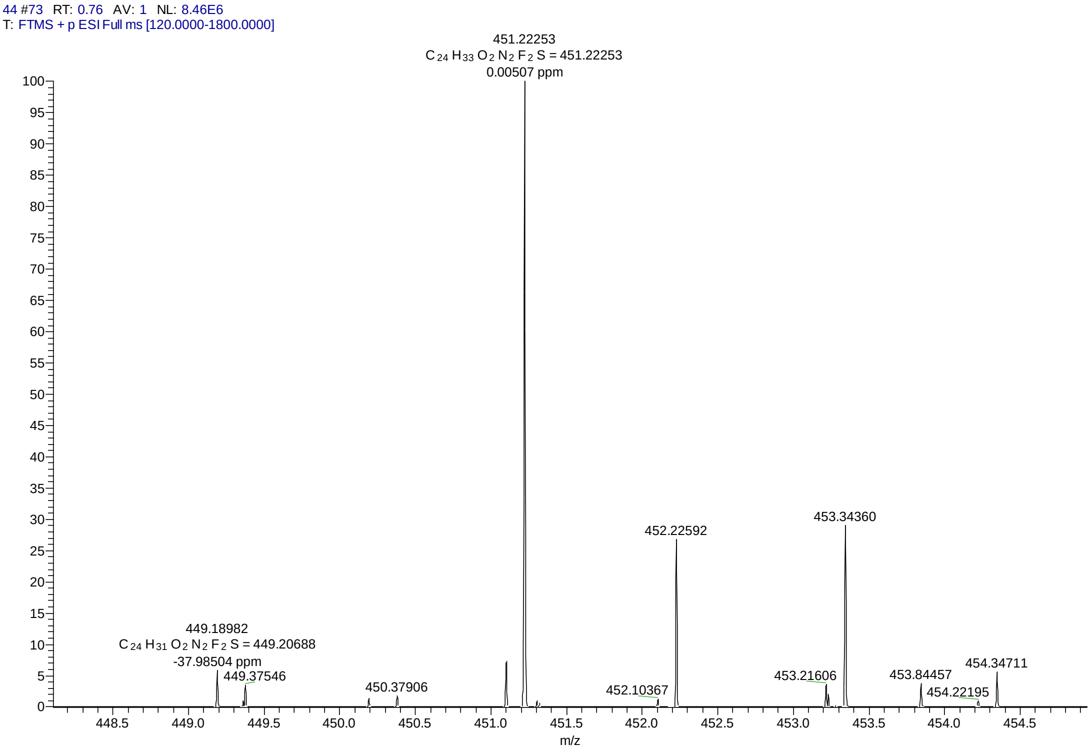

Figure S65 **H5**

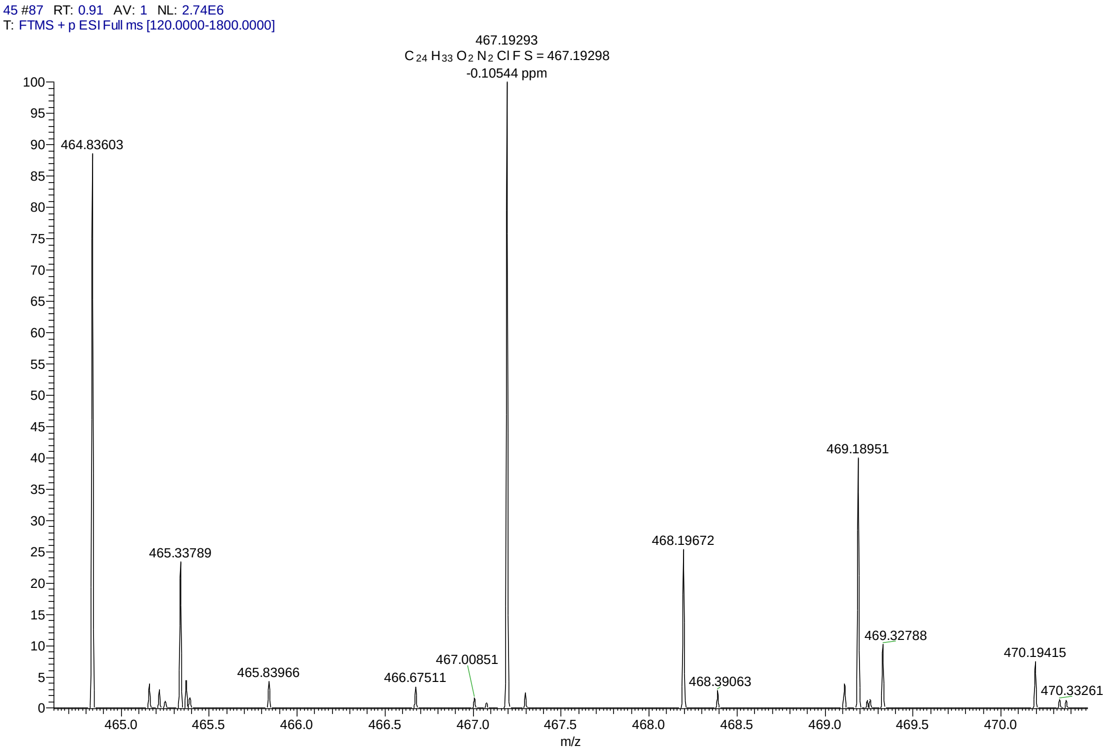

Figure S66 **H6**

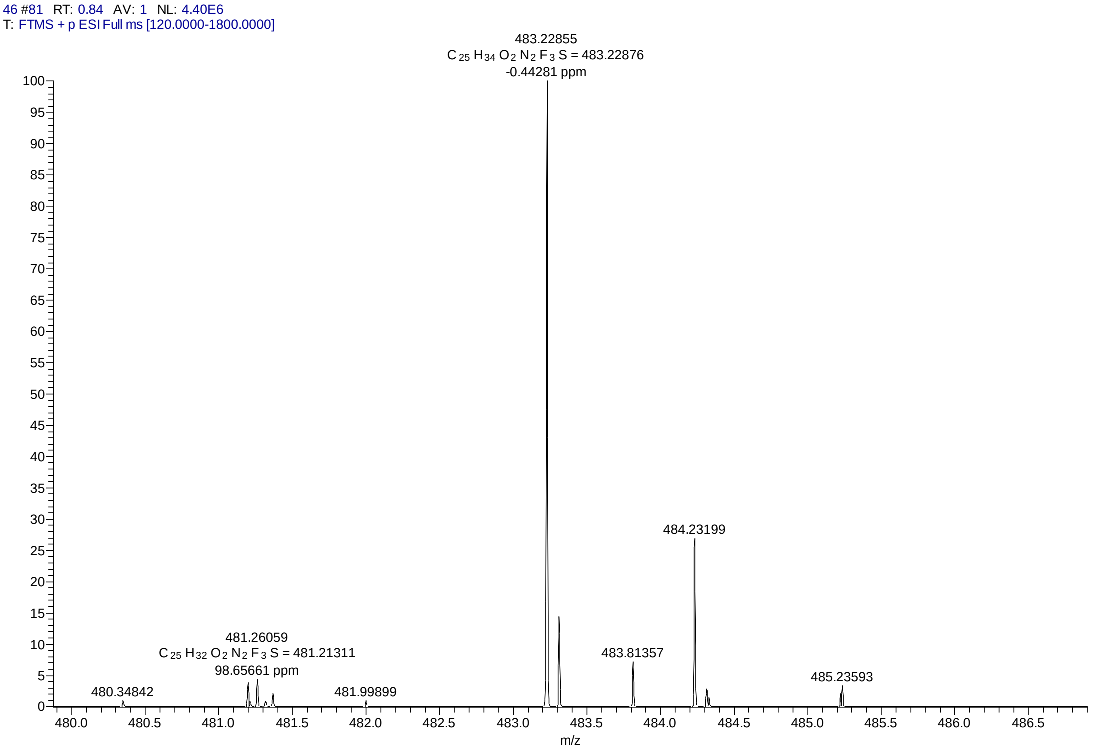

Figure S67 **H7**

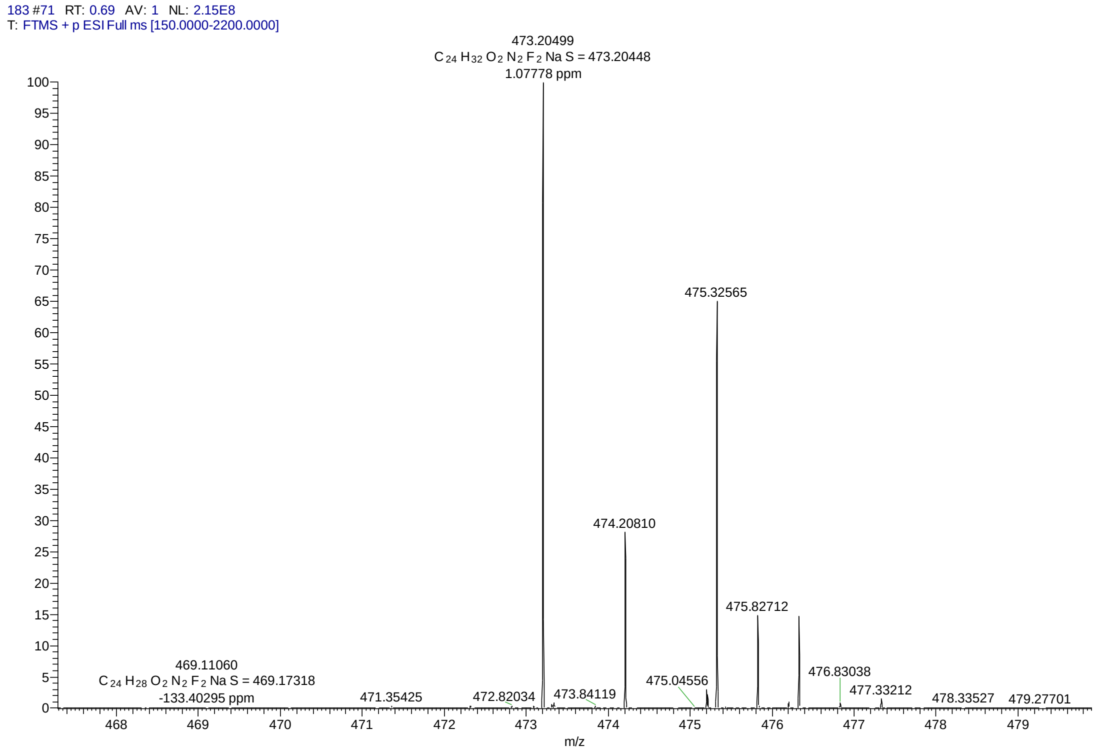

Figure S68 **H8**

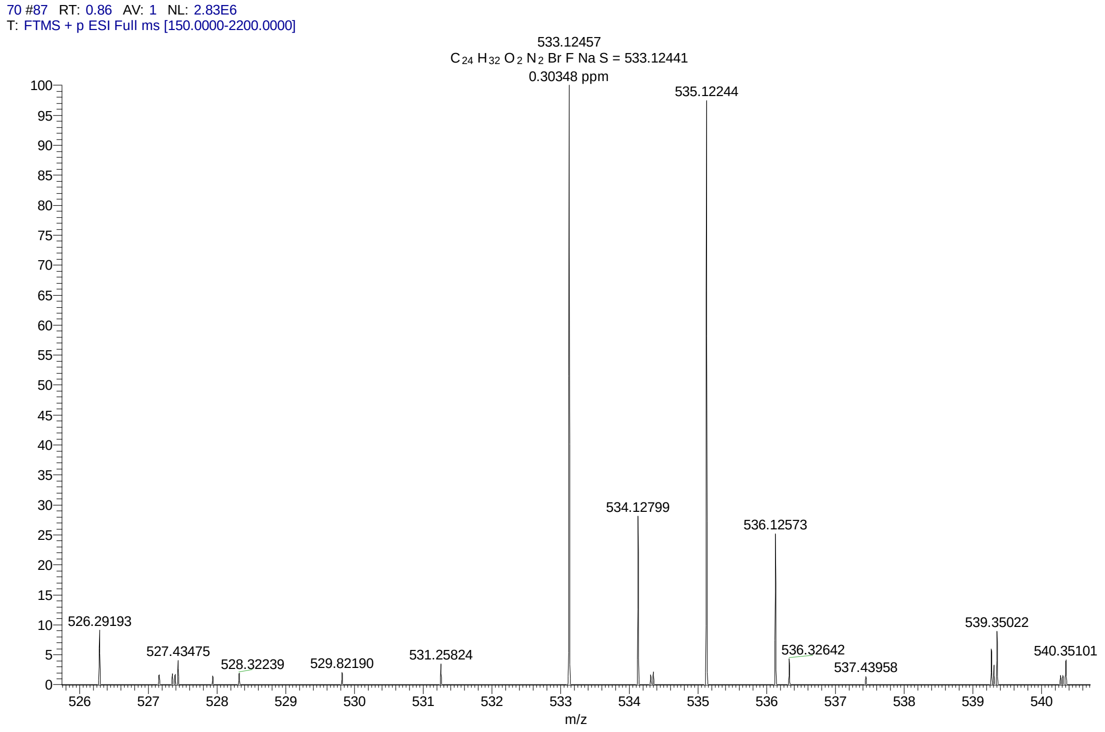

Figure S69 **H9**

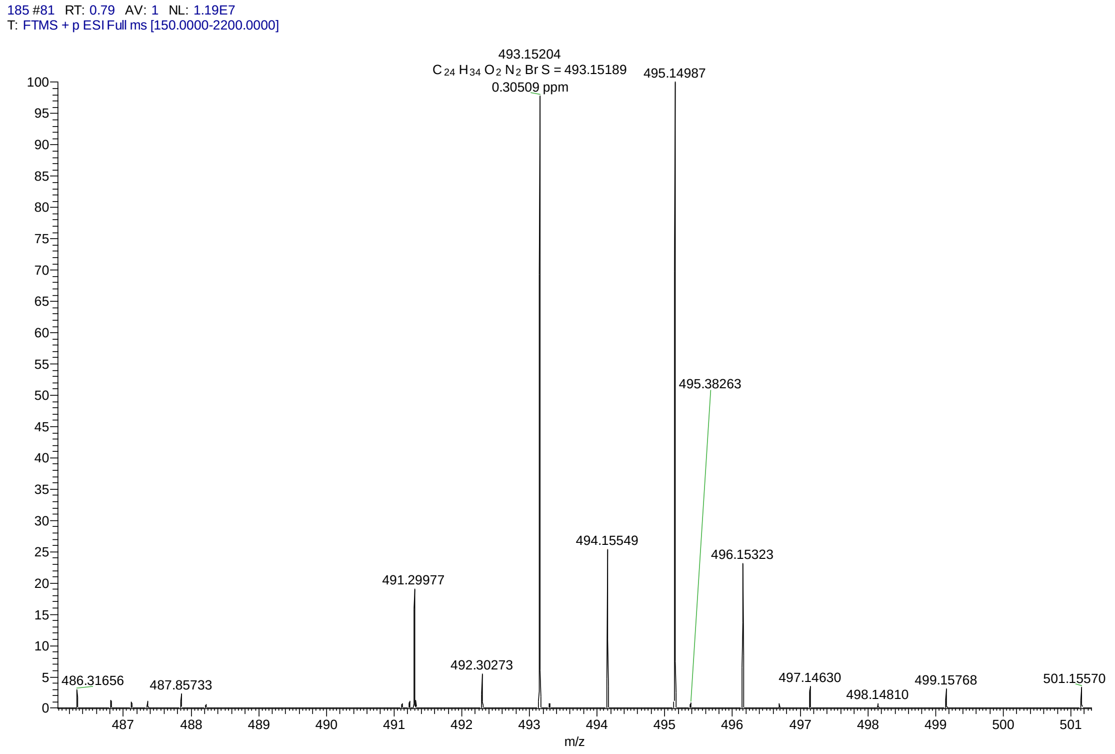

Figure S70 **H10**

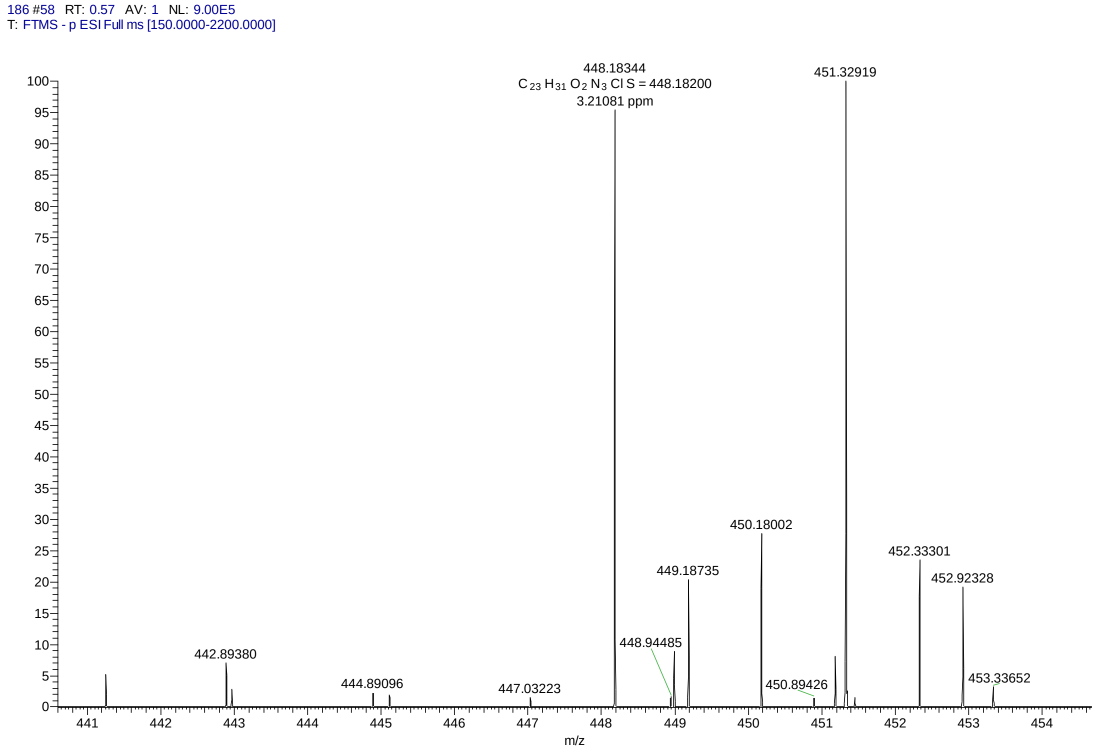

Figure S71 **H11**

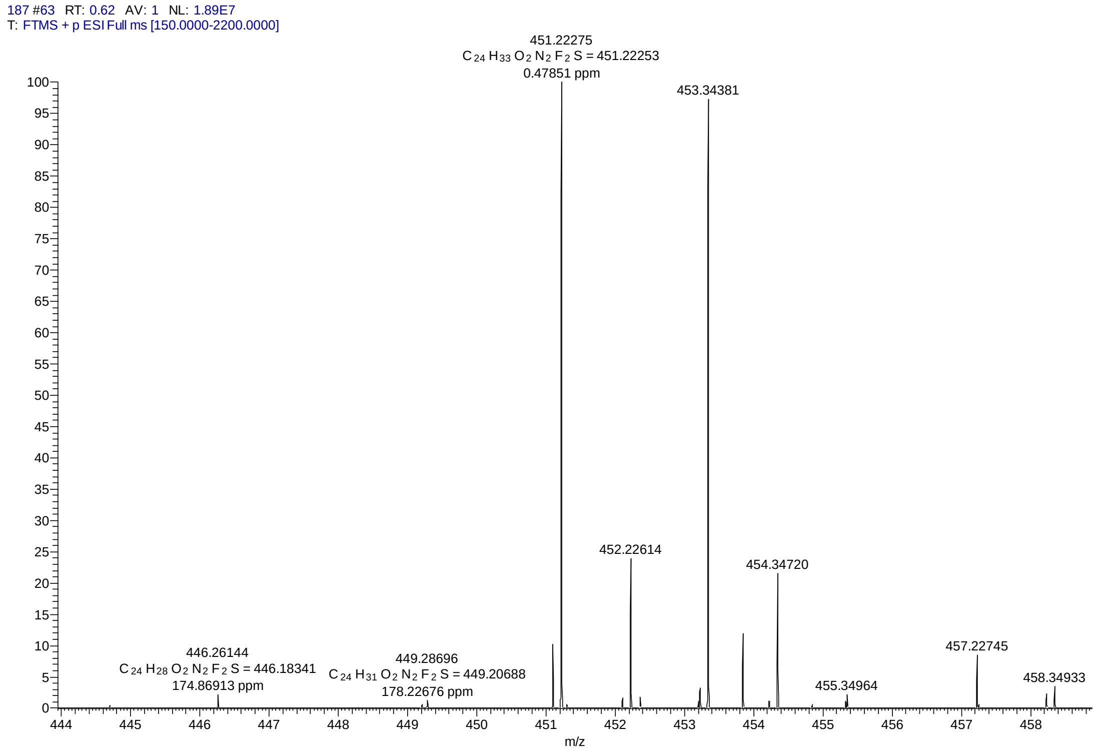

Figure S72 **H12**

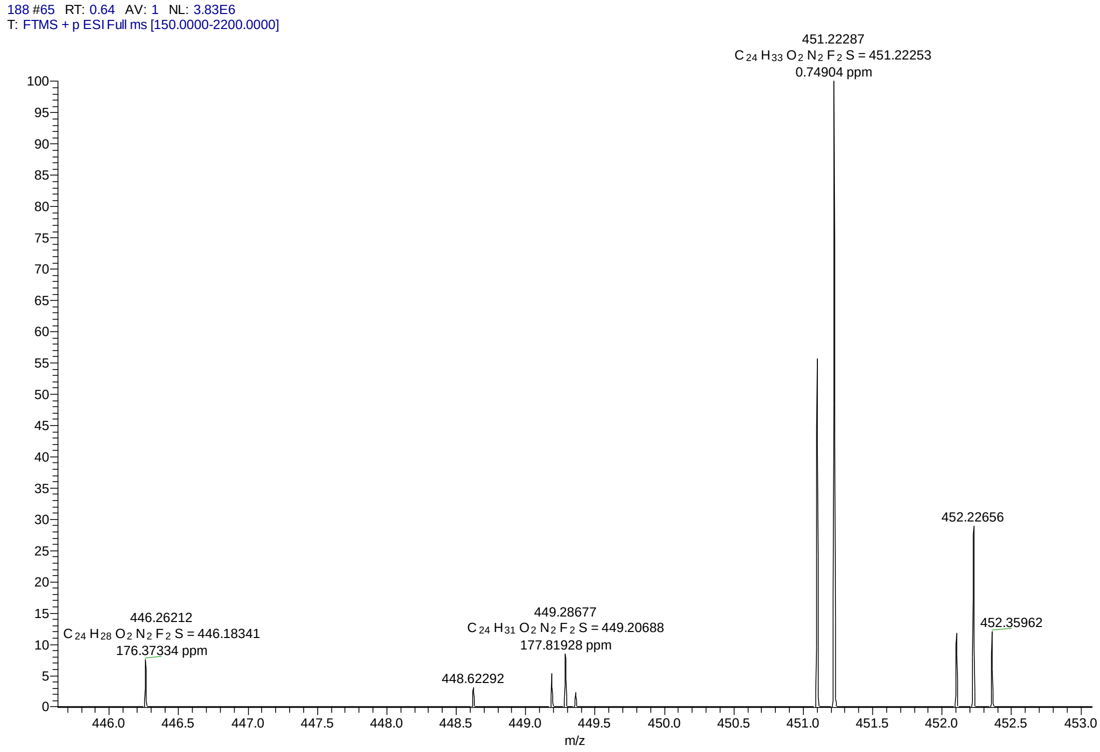

Figure S73 **H13**

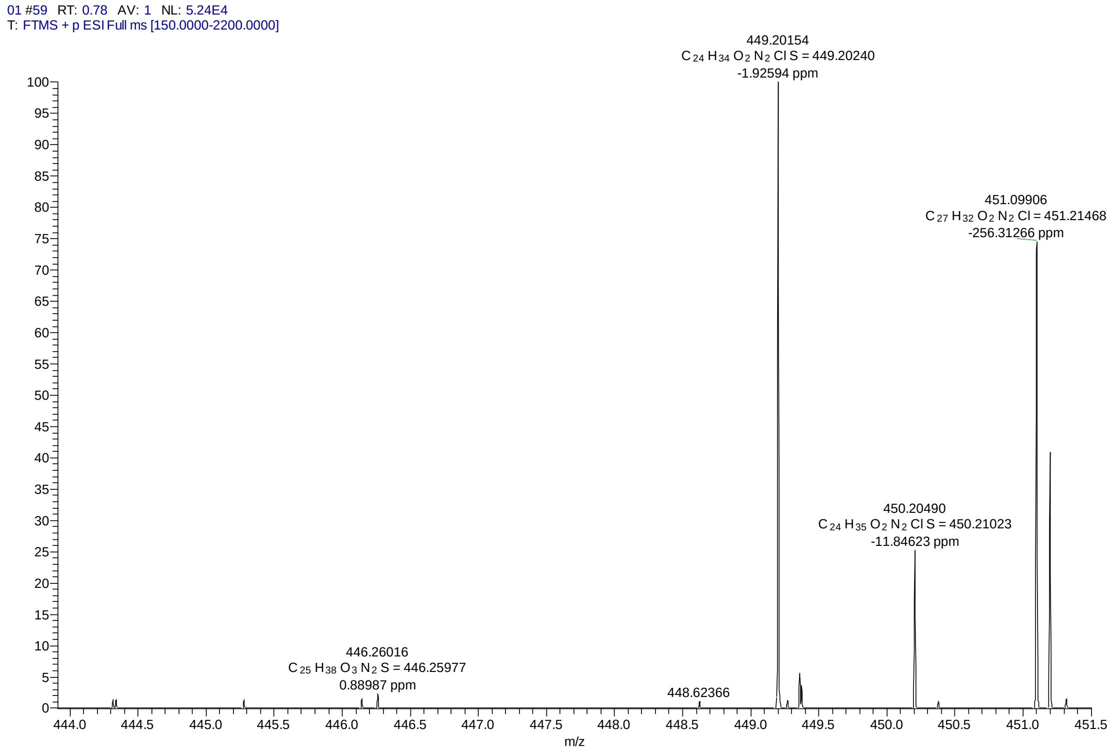

Figure S74 **H14**

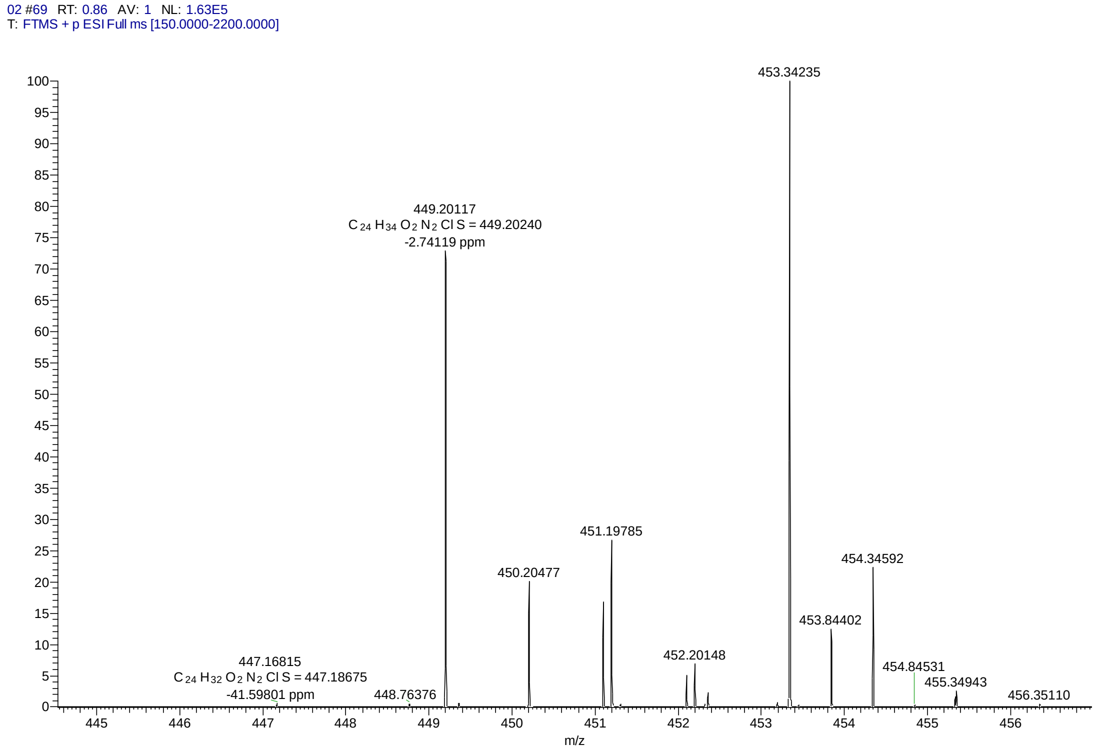

Figure S75 **H15**

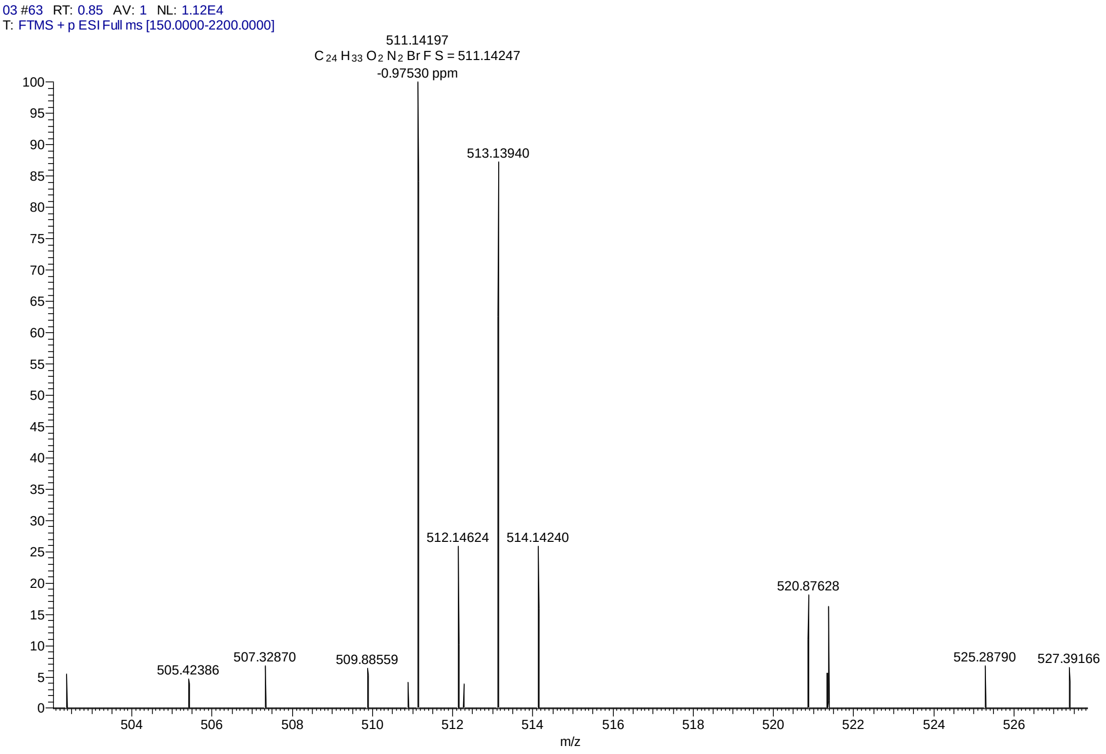

Figure S76 **H16**

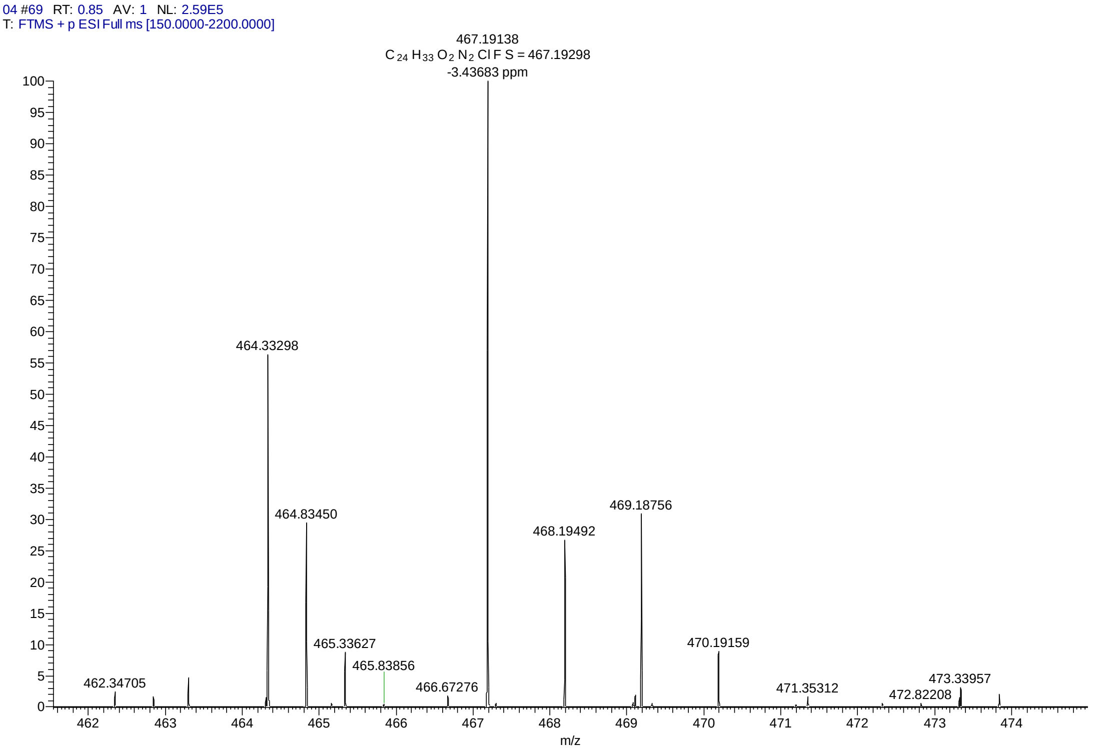

Figure S77 **H17**

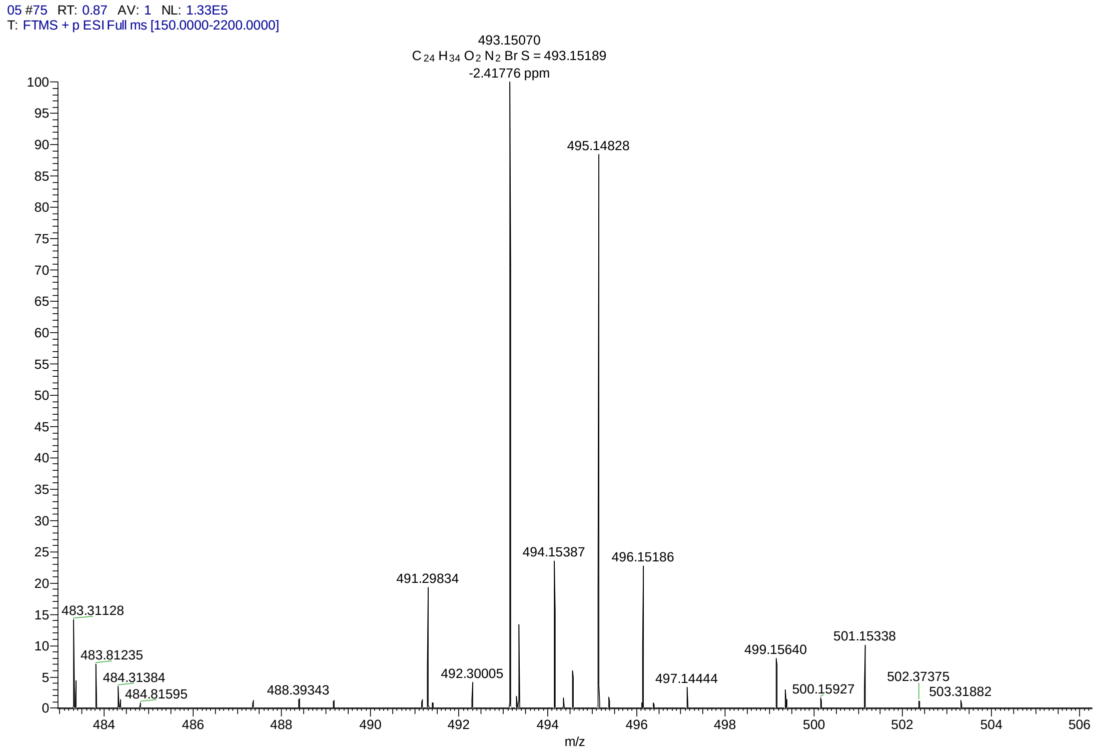

Figure S78 **H18**

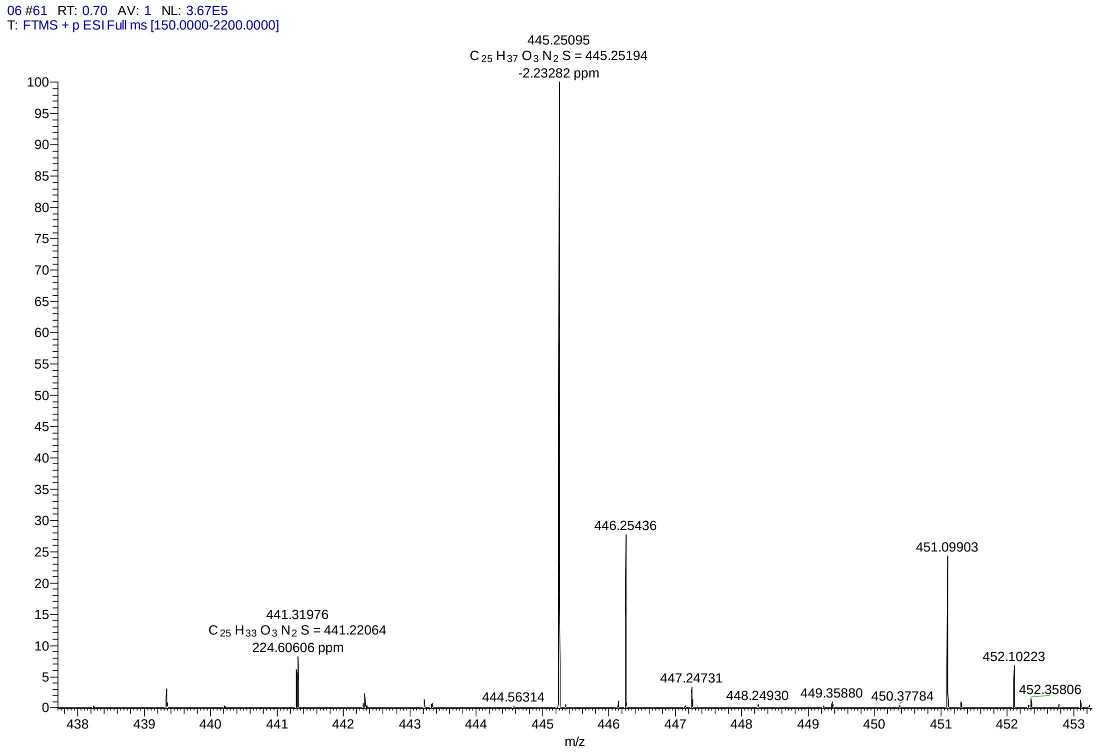

Figure S79 **H19**

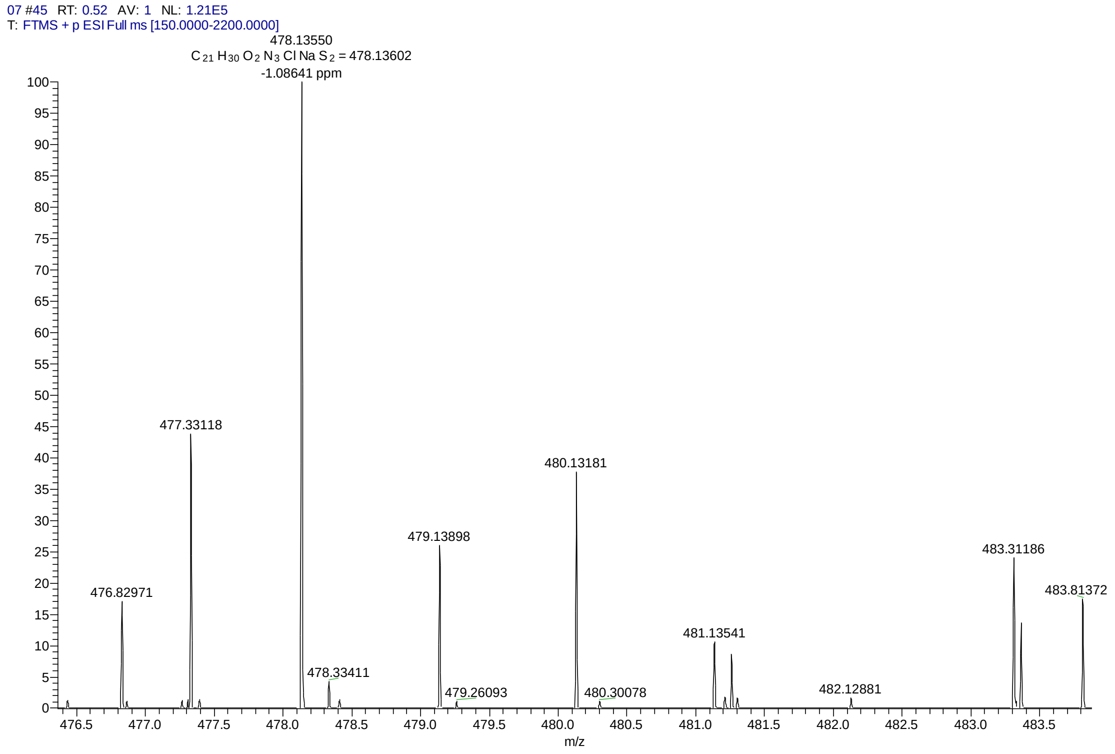

Figure S80 **H20**

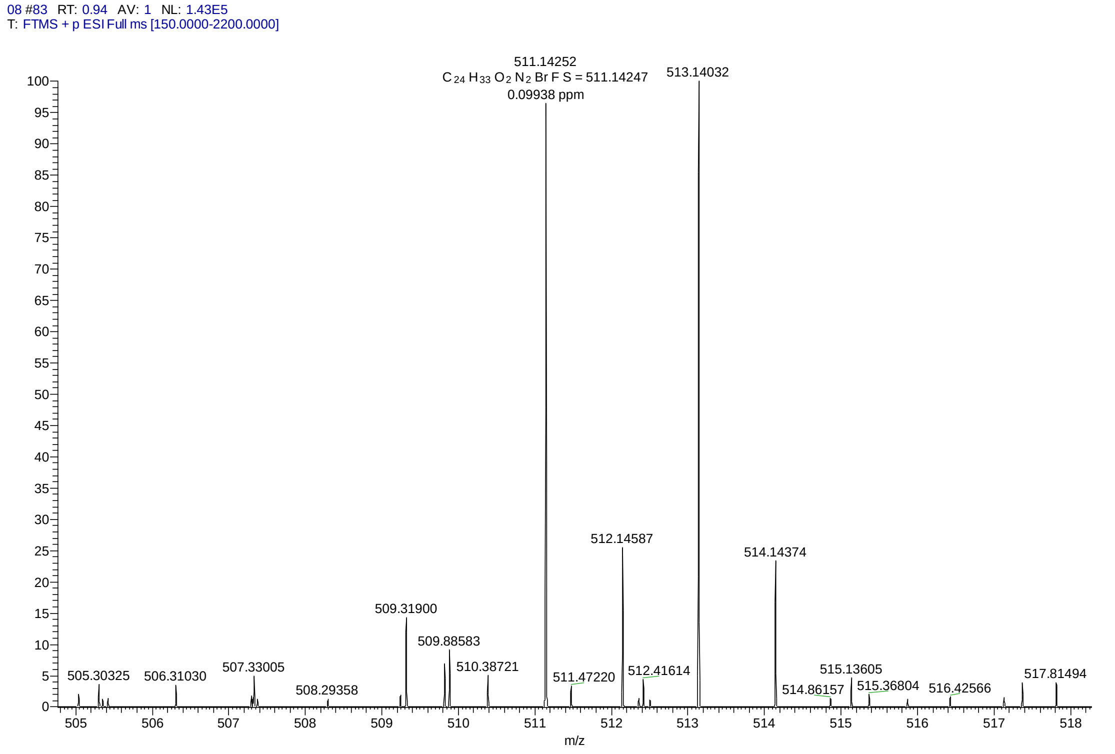

Figure S81 **H21**

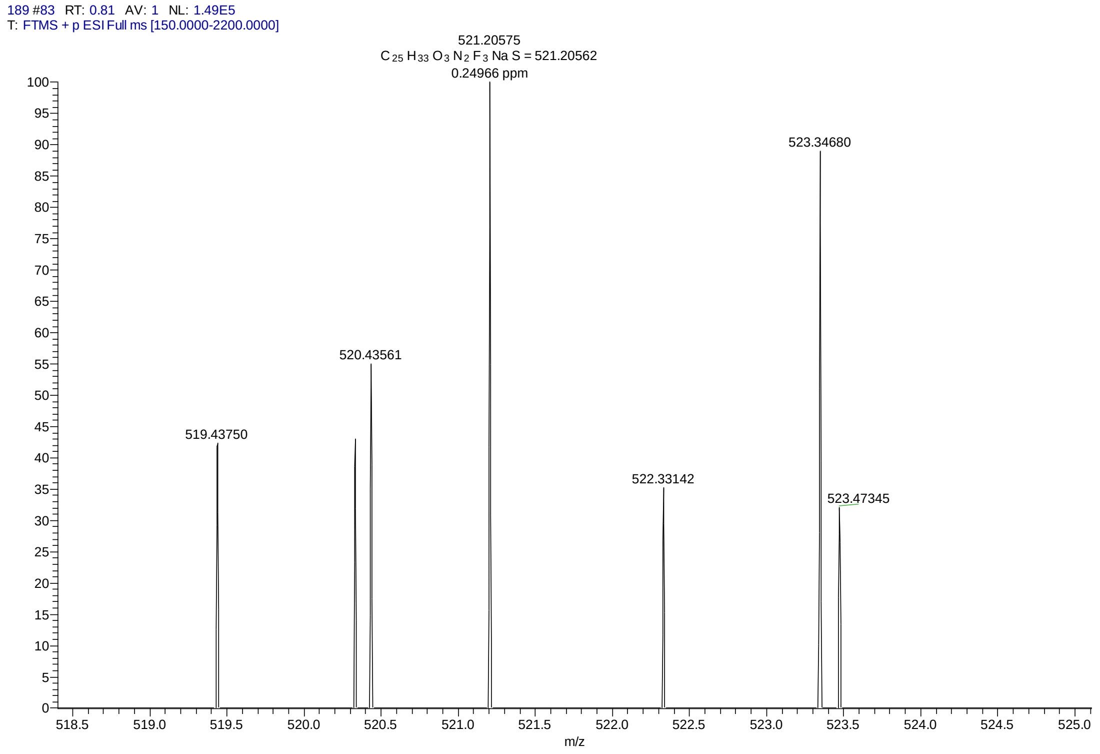

Figure S82 **H22**

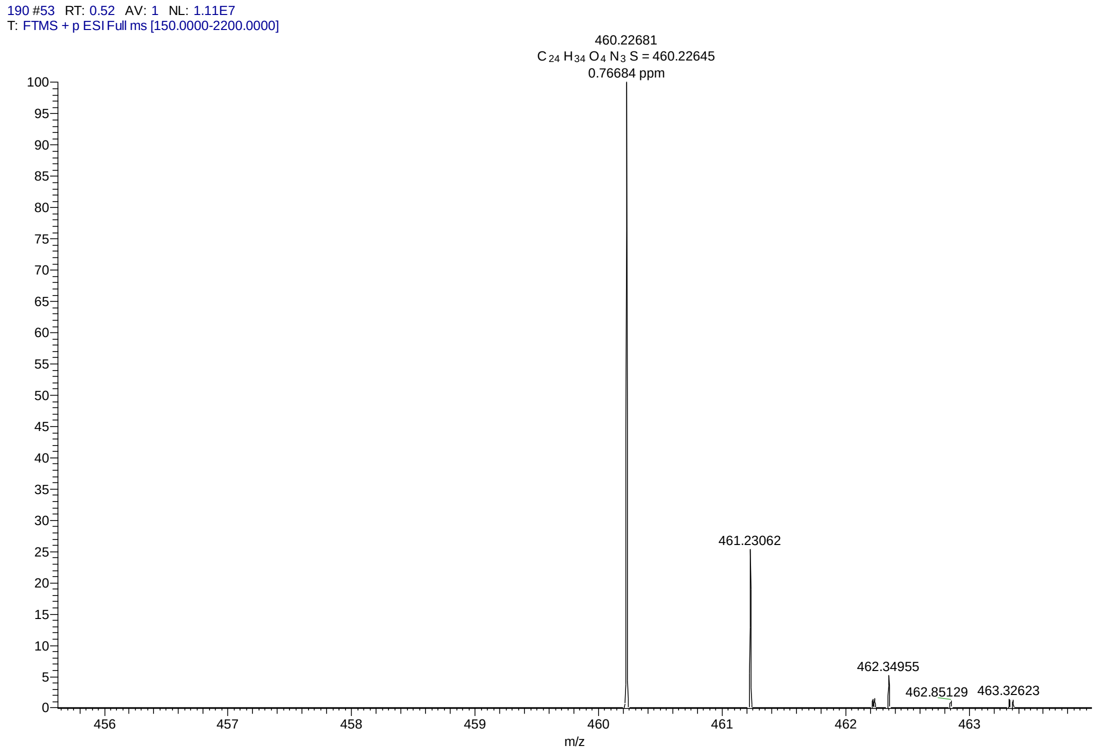

Figure S83 **H23**

4. Reference

[1] Zhang, R. F., Guo, S. X., Deng, P., Wang, Y., Dai, A. L., and Wu, J. (2021). Novel Ferulic Amide Ac6c Derivatives: Design, Synthesis, and Their Antipest Activity. *J. Agric. Food Chem*. 69, 10082-10092. doi: 10.1021/acs.jafc.1c03892.

[2] Zhang, R. F., Deng, P., Dai, A. L., Guo, S. X., Wang, Y., Wei, P. P., and Wu, J. (2021). Design, Synthesis, and Biological Activity of Novel Ferulic Amide Ac5c Derivatives. *ACS Omega*. 6, 27561-27567. doi: 10.1021/acsomega.1c04644.

[3] Luo, D. X., Guo, S. X., He, F. Chen, S. H., Dai, A. L., R. F. Zhang, R. F., and Wu, J. (2020). Design, Synthesis, and Bioactivity of α-Ketoamide Derivatives Bearing a Vanillin Skeleton for Crop Diseases. *J. Agric. Food Chem*., 68, 7226-7234. doi:10.1021/acs.jafc.0c00724.

[4] Wang, Y. Y., Xu, F. Z., Luo, D. X., Guo, S. X., He, F., Dai, A. L., Song, B. A., and Wu, J. (2019). Synthesis of anthranilic diamide derivatives containing moieties of trifluoromethylpyridine and hydrazone as potential anti-viral agents for plants. *J. Agric. Food Chem.* 67, 13344-13352. doi:10.1021/acs.jafc.9b05441.

[5] Ren, X. L., Li, X. Y., Yin, L. M., Jiang, D. H., and Hu, D. Y. (2020). Design, Synthesis, Antiviral Bioactivity, and Mechanism of the Ferulic Acid Ester-Containing Sulfonamide Moiety. *ACS Omega*. 5, 19721-19726. doi:10.1021/acsomega.0c02421.
